# A phenome-wide association and factorial Mendelian randomization study on the repurposing of uric acid-lowering drugs for cardiovascular outcomes

**DOI:** 10.1007/s10654-024-01138-0

**Published:** 2024-07-11

**Authors:** Lijuan Wang, Ines Mesa-Eguiagaray, Harry Campbell, James F Wilson, Veronique Vitart, Xue Li, Evropi Theodoratou

**Affiliations:** 1 Centre for Global Health, Usher Institute, The University of Edinburgh, Edinburgh, UK; 2 MRC Human Genetics Unit, Institute of Genetics and Cancer, The University of Edinburgh, Edinburgh, UK; 3 School of Public Health and the Second Affiliated Hospital, Zhejiang University School of Medicine, Hangzhou, Zhejiang China; 4 Cancer Research UK Edinburgh Centre, MRC Institute of Genetics and Cancer, The University of Edinburgh, Edinburgh, UK

**Keywords:** Uric acid, Cardiovascular disease, Phenome-wide association study, Drug repurposing, Factorial Mendelian randomization

## Abstract

Uric acid has been linked to various disease outcomes. However, it remains unclear whether uric acid-lowering therapy could be repurposed as a treatment for conditions other than gout. We first performed both observational phenome-wide association study (Obs-PheWAS) and polygenic risk score PheWAS (PRS-PheWAS) to identify associations of uric acid levels with a wide range of disease outcomes. Then, trajectory analysis was conducted to explore temporal progression patterns of the observed disease outcomes. Finally, we investigated whether uric acid-lowering drugs could be repurposed using a factorial Mendelian randomization (MR) study design. A total of 41 overlapping phenotypes associated with uric acid levels were identified by both Obs- and PRS- PheWASs, primarily cardiometabolic diseases. The trajectory analysis illustrated how elevated uric acid levels contribute to cardiometabolic diseases, and finally death. Meanwhile, we found that uric acid-lowering drugs exerted a protective role in reducing the risk of coronary atherosclerosis (OR = 0.96, 95%CI: 0.93, 1.00, *P* = 0.049), congestive heart failure (OR = 0.64, 95%CI: 0.42, 0.99, *P* = 0.043), occlusion of cerebral arteries (OR = 0.93, 95%CI: 0.87, 1.00, *P* = 0.044) and peripheral vascular disease (OR = 0.60, 95%CI: 0.38, 0.94, *P* = 0.025). Furthermore, the combination of uric acid-lowering therapy (e.g. xanthine oxidase inhibitors) with antihypertensive treatment (e.g. calcium channel blockers) exerted additive effects and was associated with a 6%, 8%, 8%, 10% reduction in risk of coronary atherosclerosis, heart failure, occlusion of cerebral arteries and peripheral vascular disease, respectively. Our findings support a role of elevated uric acid levels in advancing cardiovascular dysfunction and identify potential repurposing opportunities for uric acid-lowering drugs in cardiovascular treatment.

**Supplementary Information:**

The online version contains supplementary material available at 10.1007/s10654-024-01138-0.

## Introduction

Uric acid is synthesized mainly in the liver and is the end product of purine metabolism [1]. The kidney is a major site foruric acid excretion regulation, whereby serum urate is freely filtered in glomeruli and approximately 90% of filtered urate is reabsorbed in the proximal tubules [2]. Uric acid has a role in many biological processes such as peroxynitrite scavenging, anti-oxidation mechanisms, tissue healing and induction of protective antibody responses [3]. However, elevated uric acid levels, known as hyperuricemia (≥ 7.0 mg/dL in men or ≥ 6.0 mg/dL in women), has been identified to be associated with multiple disease outcomes including gout, cardiovascular disease (CVD), renal disease, metabolic syndrome and type 2 diabetes [4]. While hypouricemia caused by reduced uric acid level (≤ 2.0 mg/dL) has been correlated with neurodegenerative diseases [5]. 

This study employed a phenome-wide association study (PheWAS) strategy to examine the associations between uric acid levels and thousands of phenotypes in a high throughput manner. PheWAS has been proposed as a hypothesis-searching method to systematically examine the relationship between an exposure and a broad range of outcomes [6]. Traditionally, observational PheWAS (Obs-PheWAS) can be used to explore the associations of uric acid levels on various phenotypes using the measurement value at the time of recruitment. In addition, given the established role of genetic factors in the regulation of uric acid levels, [7, 8] as indicated by previous genome-wide association studies (GWASs) of serum urate levels, [9–12] the utilization of polygenic risk score-based PheWAS (PRS-PheWAS) becomes crucial for detecting the causal impact of uric acid on health outcomes. Thus, both Obs- and PRS- PheWASs were conducted in the current study, with the aim of harnessing the strengths from different perspectives and, as a result, identifying consistent phenotypic associations with uric acid.

Furthermore, a comprehensive investigation of disease progression patterns following elevated uric acid is also of importance, which can assist the identification of key pathways linking the biomarker with subsequent somatic and mental health problems and thus propose interventions to prevent corresponding health declines. Disease trajectory analysis is an ideal approach to achieve this purpose, which explores the networks of disease progression over time [13]. By investigating the temporal order of a set of associated diseases, this method provides a foundation for examining causal relationships and sequential patterns of multiple morbidities.

Meanwhile, for PheWAS-identified diseases associated with high uric acid levels, whether the repurposing of uric acid-lowering therapy can be considered as a viable treatment strategy warrants thorough investigation. With the merit of Mendelian randomization (MR), an epidemiological method utilizing genetic data as instruments to reinforce causal inference between an exposure and outcome, [14] we conducted a drug repurposing analysis by leveraging genetic variants associated with uric acid-lowering drug targets and evaluating their potential clinical effectiveness in the context of disease. In addition, a factorial MR design has recently been developed to mimic randomized clinical trials and evaluate the joint effect of two interventions in the treatment of medical conditions [15]. Using this design, we also evaluated the effectiveness of combination therapies involving uric acid-lowering agents alongside established treatments for the observed conditions.

## Methods

### Study design

**Fig. 1:**
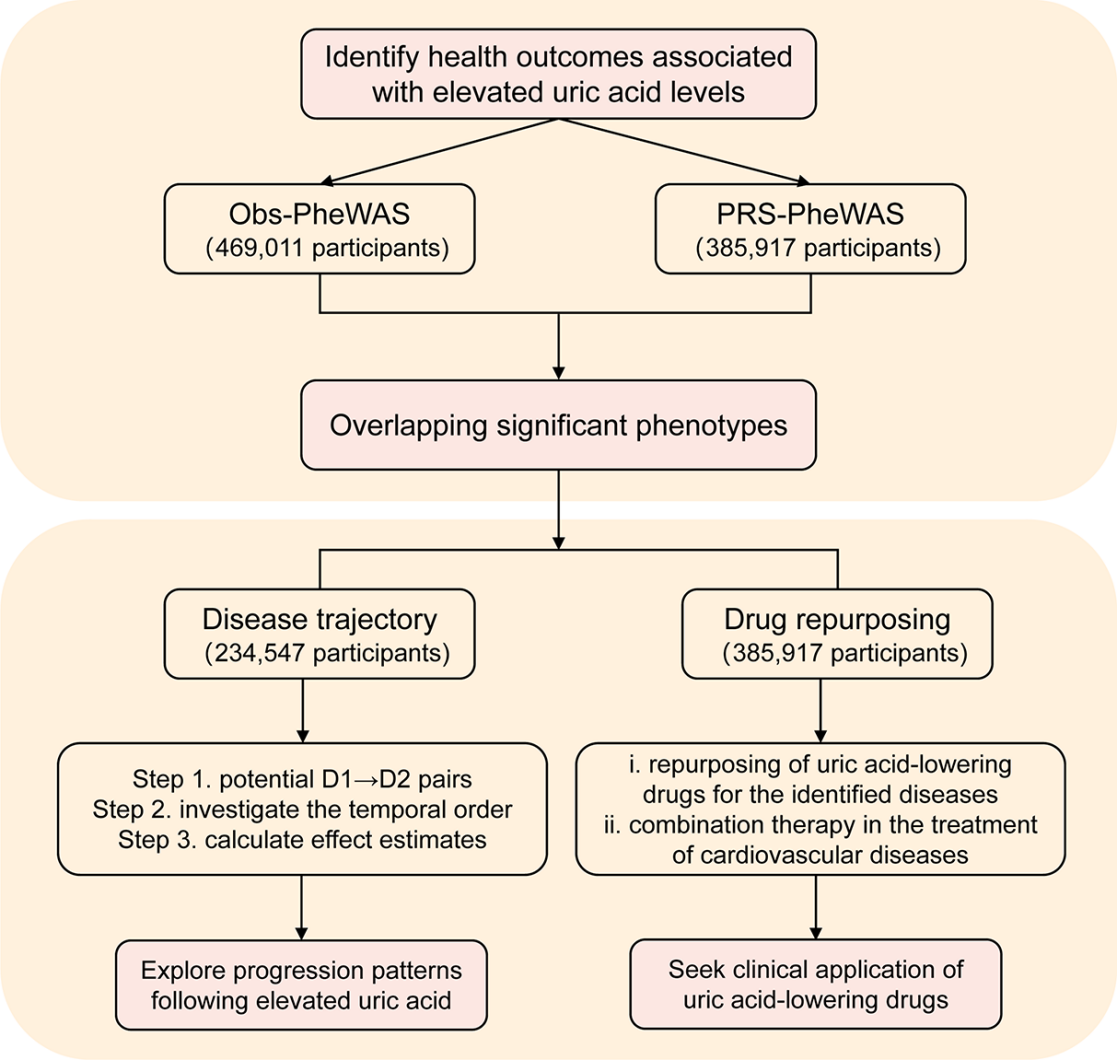
Flowchart of the study design

First, we conducted both Obs-PheWAS and PRS-PheWAS to examine the links between uric acid levels and a broad spectrum of disease outcomes. Then, for those disease outcomes identified as significant through both approaches, we conducted trajectory analysis to investigate patterns of disease progression following elevated uric acid levels. Meanwhile, we conducted drug repurposing analysis to assess potential therapeutic benefits of uric acid-lowering medications for the associated cardiovascular diseases. More details of the study design are presented in Fig. 1.

### UK biobank

The UK Biobank is a population-based prospective cohort study, which recruited over 500,000 adult participants aged between 40 and 69 years in 2006–2010. The study combined extensive measurements of baseline data and genotype data with linked health records [16]. The genotype array and quality control (QC) of the genetic data have been described elsewhere [17]. In order to minimize the influence of diverse population structure, we excluded participants with high heterozygosity or with high missing rate, sex mismatch or putative aneuploidy in sex chromosome, non-European ancestry (to minimize population structure bias), or high relatedness. After filtering ineligible samples, the current study was based on 385,917 UK Biobank participants. More details regarding sample quality control can be found in Supplementary Fig. 1.

### Phenome framework and PheWAS analysis

We included records from three types of national medical registries (i.e., in-patient hospital episode, cancer registry, and death registry records) until March 31, 2023 to create the phenome framework. The ontology of the phenome was defined based on the International Classification of Diseases (ICD) codes in the EHRs. Records date back to 1997 for England, 1998 for Wales and 1981 for Scotland. All of the current UK Biobank linked English and most Welsh hospital data are coded in ICD10. However, because the collection of Scottish data collection began in 1981, the Scottish data collected prior to 1997 are coded in ICD-9, and small number of Welsh records are coded with ICD-9. Thus, we included both ICD-9 and ICD-10 codes in our PheWAS analysis. Individual ICD codes could not be directly used to define the phenome, as they represent specific sub-phenotypes of a similar set of outcomes, instead of independent phenotypes. To account for the correlations between ICD codes, we defined the phenome framework using the PheCODE schema that combines one or more related ICD codes into distinct outcome groups [18]. For a given phenotype, the case group included patients recorded as having the specific phecode that most closely related to the aetiology of the disease, and the control group was defined based on the absence of the specific phecode. In addition, participants that have similar or potentially overlapping disease states were also excluded from the control group (e.g., excluding type 1 diabetes from being in the control group when analyzing the phenotype of type 2 diabetes) [19]. To maintain statistical power, we excluded outcomes with less than 200 cases [20]. 

In Obs-PheWAS, serum uric acid was measured by uricase PAP analysis on a Beckman Coulter AU5800. We used measurement data from the initial assessment visit (involving ~ 500,000 participants) as the exposure, and included only incident cases diagnosed after six months of uric acid assessment in the analysis. A multivariable logistic regression model was utilized to investigate the odds ratios (ORs) and corresponding confidence intervals (CIs) of the linear associations between uric acid levels and the risk of diseases outcomes with adjustment for age, sex, BMI, Townsend deprivation index, smoking, physical activity and alcohol drinking. We selected common variables known to impact a wide range of diseases to reduce type I error (false positives) while maintaining statistical power, rather than focusing on disease-specific confounders that are unrelated to the majority of disease outcomes and may introduce bias into association estimates.

In PRS-PheWAS, we first generated a genetic proxy for uric acid levels based on the genetic variants from the most recent and largest urate GWAS conducted in 288,649 individuals of European ancestry [11]. We selected variants that were associated with the biomarker at genome-wide significance (*P* < 5 × 10^− 8^) and clumped them using a linkage disequilibrium (LD) threshold of r^2^ < 0.01 according to the European reference panel of the 1000 Genomes project. As a result, a total of 123 independent SNPs were identified as instruments (Supplementary Table 1). Then, a weighted polygenic risk score (PRS) for uric acid was calculated using individual-level data in the UK Biobank weighted by each variant’s association with the change in urate levels in milligrams per deciliter. Finally, a multivariable logistic regression was used to explore the linear associations between the score and phecodes with adjustment for age, sex, assessment center, and the first 10 genetic principal components (PCs). The ORs and corresponding 95% CIs of outcomes were scaled to one-standard deviation (SD) increase in genetically predicted uric acid levels.

For both Obs- and PRS- PheWASs, sex-stratified analyses were performed to test heterogeneity of effects among men and women populations. The Benjamini-Hochberg method was applied to account for multiple testing and associations with a FDR < 0.05 were considered significant. The PheWAS analysis was implemented by using the “PheWAS” package (R version 4.2.1) [19]. 

### Disease trajectory analysis

To generate a more comprehensive and longitudinal perspective on disease progression over time, we extracted significant phecodes (i.e., the overlapping significant disease outcomes identified from Obs- and PRS- PheWASs) from both national medical registry and primary care records and then combined them together to generate a new phenome framework for disease trajectory analysis. As a result, the final case group for a given phenotype comprises patients from both national medical registry and primary care records, rather than the intersection of the two. Briefly, primary care EHRs were extracted from four data sources based on three EHR vendors (two in England from Vision and TPP SystmOne, and two in Scotland and Wales combining data from EMIS and Vision), covering approximately 45% of the UK Biobank participants. Different from the ICD coding system used by hospital and registry records, primary care data was coded with the Read coding system (version 2 or 3). We used a comprehensive four-step process to map read codes to phecodes, more details regarding primary care information and the mapping process can be found in Supplementary Methods. Then, the sequential disease progression patterns were explored among individuals with higher uric acid levels (above the median value). The method of disease-trajectory analysis included three interrelated steps. First, we restricted analysis to the first onset of medical conditions after recruitment and identified all possible disease 1 (D1) and disease 2 (D2) pairs if the co-occurrence of the two diseases was experienced by at least 1% of these individuals. Second, a binomial test was conducted for each disease pair to investigate whether significantly more individuals had D2 diagnosed after D1 than vice versa (e.g., FDR corrected *P* < 0.05). Third, for each disease pair D1→D2 with potential temporal order, conditional logistic regression was used to assess the odds ratios (OR) of having D2 after diagnosed with D1 with adjustment for age, sex, body mass index (BMI), Townsend deprivation index, smoking, physical activity and alcohol drinking. D1→D2 pairs considered significant (e.g., OR > 1.0 and FDR corrected *P* < 0.05) were used to construct a disease-trajectory network. We also added overall mortality as an outcome in the analysis to detect disease pairs leading to death (e.g., D1→D2→death). For each disease pair, the sample size included in the analysis was different as we excluded individuals with a prior history of the two outcomes before the follow-up period began. All the statistical analyses were conducted using R software (version 4.2.1).

### Drug repurposing analysis

We restricted our study to the overlapping significant outcomes identified by both observational and PRS-based PheWASs. Then, we conducted repurposing analysis to evaluate the effects of commonly used first-line uric acid-lowering drugs (xanthine oxidase inhibitors and uricosurics) as well as an under-investigated agent (purine nucleoside phosphorylase inhibitors) on a set of uric acid-related outcomes. We first obtained drug targets from the DrugBank database [21]. Then, genetic variants (i) located within 500 kb around the target genes; (ii) associated with serum urate levels at a P-value less than 0.05; and (iii) in low LD relationship with each other (r^2^ < 0.2) were selected as instruments to proxy uric acid-lowering effects. We set a less strict selection standard according to a prior publication, [22] aiming to include more instruments and increase the explained variance. For each drug, a weighted PRS was calculated and a multivariable logistic regression analysis was applied to estimate the impact of genetically predicted uric acid-lowering treatment on the risk of related disease outcomes, with adjustment for age, sex, assessment center and the first 10 PCs.

In order to examine the joint effect of combination therapy that integrates uric acid-lowering drugs with antihypertensive drugs in the treatment of cardiovascular diseases, a 2 × 2 factorial MR analysis was also performed. The population was first allocated into two subgroups based on the median genetic score of uric acid. Individuals of the two subgroups were further classified into two groups, respectively, accordingly to the median genetic score of blood pressure. Therefore, four groups were formed after this process namely the reference group with both genetically-predicted higher uric acid levels and blood pressure, the group with lower uric acid levels and higher blood pressure, the group with higher uric acid levels and lower blood pressure, and the group with both lower uric acid levels and blood pressure. We then estimated the risk of cardiovascular outcomes between the groups and the reference group using the multivariable logistic regression, with adjustment for age, sex, assessment center and the first 10 PCs. The tests were two-sided and performed using R software version 4.2.1.

## Results

A total of 385,917 unrelated European individuals were included in the current study, containing 177,690 (46.0%) men and 208,227 (54.0%) women. The mean age of the population was 56.7 ± 8.0 years (SD) and the mean levels of urate were 5.2 ± 1.4 milligrams per deciliter. More details regarding socio-demographic information, lifestyle factors and biomarker levels, are presented in Table 1.

**Table 1:** Baseline characteristics of participants in the UK Biobank

Baseline characteristic	All participants (*n* = 385,917)
Sex, *n* (%)	
Female	208,227 (54.0)
Male	177,690 (46.0)
**Age (years), mean (SD)**	56.7 (8.0)
**BMI (kg/m** ^**2**^ **), mean (SD)**	27.4 (4.8)
**Townsend deprivation index, mean (SD)**	-1.5 (3.0)
**Smoking status, n (%)**	
Current	40,039 (10.4)
Former	136,651 (35.4)
Never	207,296 (53.7)
Unknown	1,931 (0.5)
**Alcohol drinker status, n (%)**	
Current	359,366 (93.1)
Former	13,353 (3.5)
Never	12,246 (3.2)
Unknown	952 (0.2)
**Physical activity, n (%)**	
Light (0–2)	130,774 (33.9)
Medium [3–5]	146,642 (38.0)
Heavy (6–7)	89,607 (23.2)
Unknown	18,894 (4.9)
**Blood pressure (mmHg), mean (SD)**	
SBP	139.8 (19.7)
DBP	82.2 (10.7)
**Biomarker concentrations (mg/dL), mean (SD)**	
Urate	5.2 (1.4)
Total cholesterol	220.8 (44.1)
LDL cholesterol	138.0 (33.6)
HDL cholesterol	56.2 (14.8)
Triglycerides	154.9 (90.6)

BMI, body mass index; DBP, diastolic blood pressure; SBP, systolic blood pressure; SD, standard deviation

### Phenome-wide associations

**Fig. 2:**
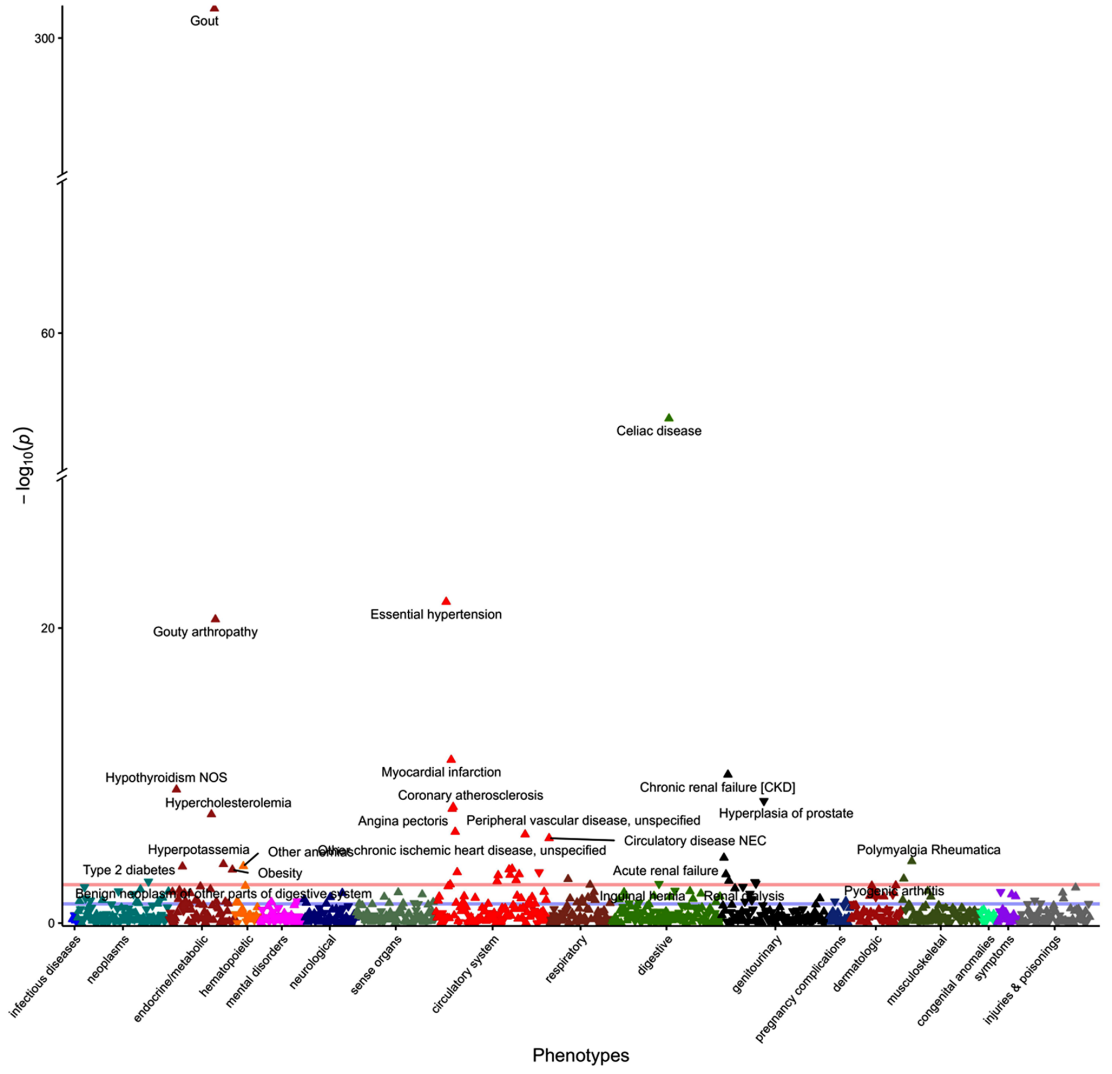
Overlapping significant phenotypes identified by both observational- and PRS-based PheWASs

In total 1,055 health and mortality-related [all-cause mortality and disease-specific mortality (e.g. circulatory disease, endocrine/metabolic disease, respiratory disease, digestive disease, genitourinary disease, neoplasm, mental disorder, neurological disease, hematopoietic disease, dermatologic disease and musculoskeletal disease) outcomes were included in the PheWAS. In Obs-PheWAS, uric acid levels were significantly associated with 397 phenotypes after FDR correction (2). In PRS-PheWAS, genetically predicted uric acid levels were related to the risk of 53 medical conditions (Supplementary Table 3). A total of 41 overlapping disease outcomes with consistent effect directions were identified by both methods (Fig. 2, Supplementary Table 4), including 17 circulatory diseases (e.g. essential hypertension), 7 endocrine/metabolic diseases (e.g. gout), 7 genitourinary diseases (e.g. chronic renal failure), 2 musculoskeletal diseases (e.g. polymyalgia rheumatica), 2 digestive diseases (e.g. celiac disease), 2 infectious diseases (e.g. tuberculosis), 1 respiratory disease (pneumonia), 1 hematopoietic disease (anemia) and 1 neoplasm (benign neoplasm of digestive system). Furthermore, higher uric acid levels were significantly related to increased risk in circulatory-specific mortality. For the observed linear associations, we also explored the co-existence of non-linear relationships by using a restricted cubic spline (RCS) function [23] with five knots located at the 5th, 25th, 50th, 75th, and 95th percentiles of genetically predicted uric acid levels. The results revealed that the associations of uric acid levels with gout, celiac disease and benign neoplasm of digestive system displayed not only a basic overall linear trend, but also a more complcated non-linear relationship. Detailed information about the effect estimates can be found in Supplementary Table 4.

In addition, sex-stratified analysis was conducted to examine differences between male and female subgroups. The results showed that the majority of the effect estimates were consistent across sex with a heterogeneity *P* < 0.05, except for cerebral artery occlusion and pyogenic arthritis. The association between high uric acid levels and increased risk of cerebral artery occlusion remains significant exclusively among women (*N* = 2033, OR = 1.25, 95%CI: 1.12, 1.40, *P* = 4.24 × 10^− 5^) but not men (*N* = 3117, OR = 1.05, 95%CI: 0.96, 1.15, *P* = 0.259). While males with high uric acid levels are prone to develop pyogenic arthritis (*N* = 388, OR = 1.82, 95%CI: 1.43, 2.33, *P* = 1.55 × 10^− 6^) instead of females (*N* = 226, OR = 0.87, 95%CI: 0.63, 1.20, *P* = 0.385) (Supplementary Table 5). We also examined the effects of uric acid on the risk of associated kidney diseases both within general population and male and female subgroups, and found that high uric acid levels were significantly related to increased risk of kidney outcomes, particularly among participants with lower eGFR levels. However, no considerable heterogeneous effects were identified between the low-level and high-level eGFR groups, indicating the absence of interaction (Supplementary Table 6).

### Disease trajectories following elevated uric acid levels

**Fig. 3:**
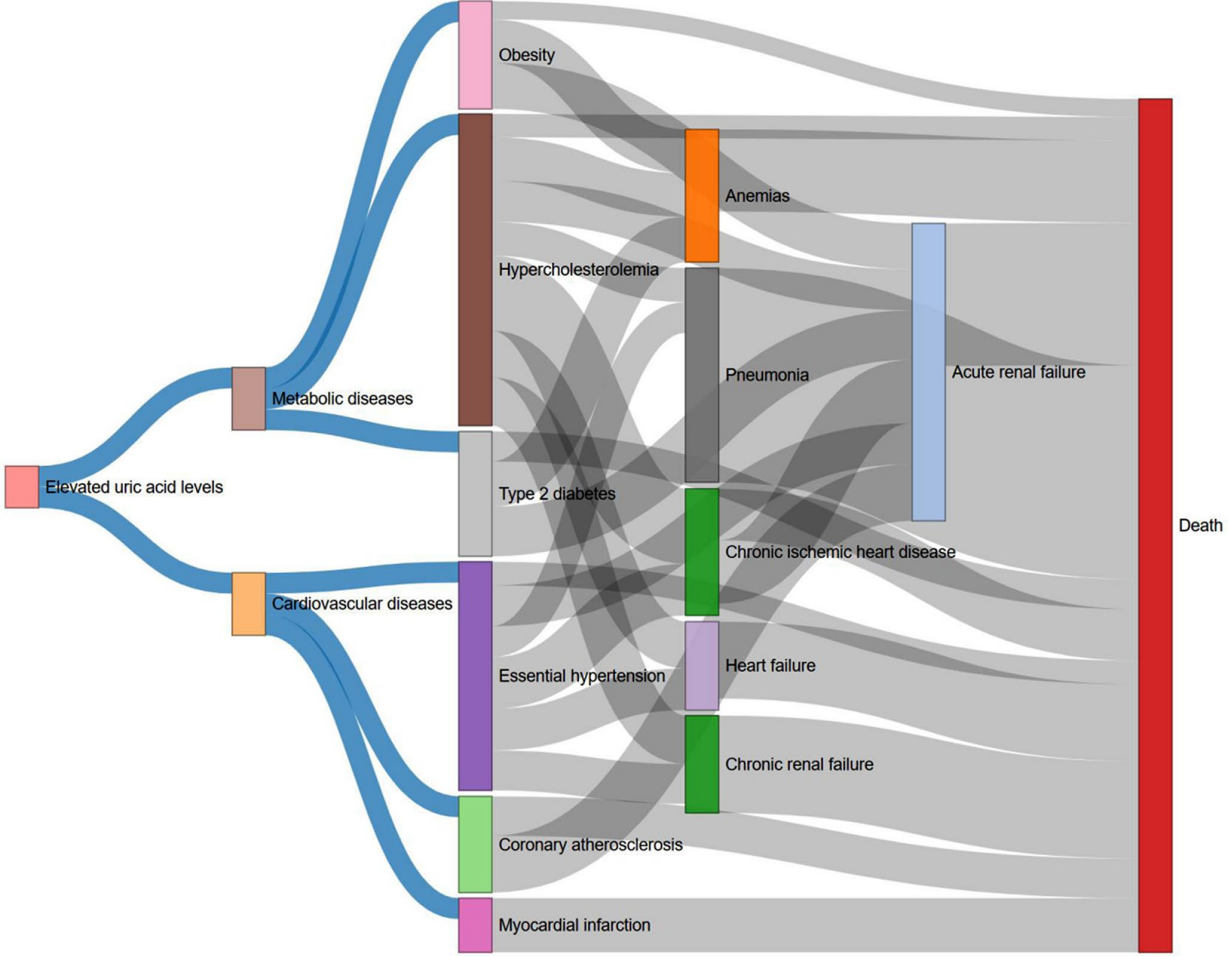
Disease progression patterns subsequent to elevated uric acid levels

Among a total of 595 possible disease pairs established for 35 unique health outcomes that were at an increased risk with elevated uric acid levels, 90 were retained based on the selection criteria in step 1. Then, step 2 identified 43 significant D1→D2 pairs with clear temporal orders. Step 3 estimated the effect size of developing D2 after diagnosed with D1 and all 43 pairs survived in the multiple correction test. We categorized these disease pairs according to the similarity of their underlying affected systems or their etiologies. As a result, one cluster mainly including diseases of cardiometabolic system was identified, where the disease tree thrived after the diagnoses of obesity, type 2 diabetes, hypercholesterolemia, [24] essential hypertension, coronary atherosclerosis and myocardial infarction, followed by anemia, pneumonia, heart failure, renal failure, and finally ended up on death (Fig. 3). Details regarding the number of disease pairs remaining at each step and the effect estimates are shown in Supplementary Fig. 2 and Supplementary Table 7.

### Repurposing of uric acid-lowering drugs

**Fig. 4:**
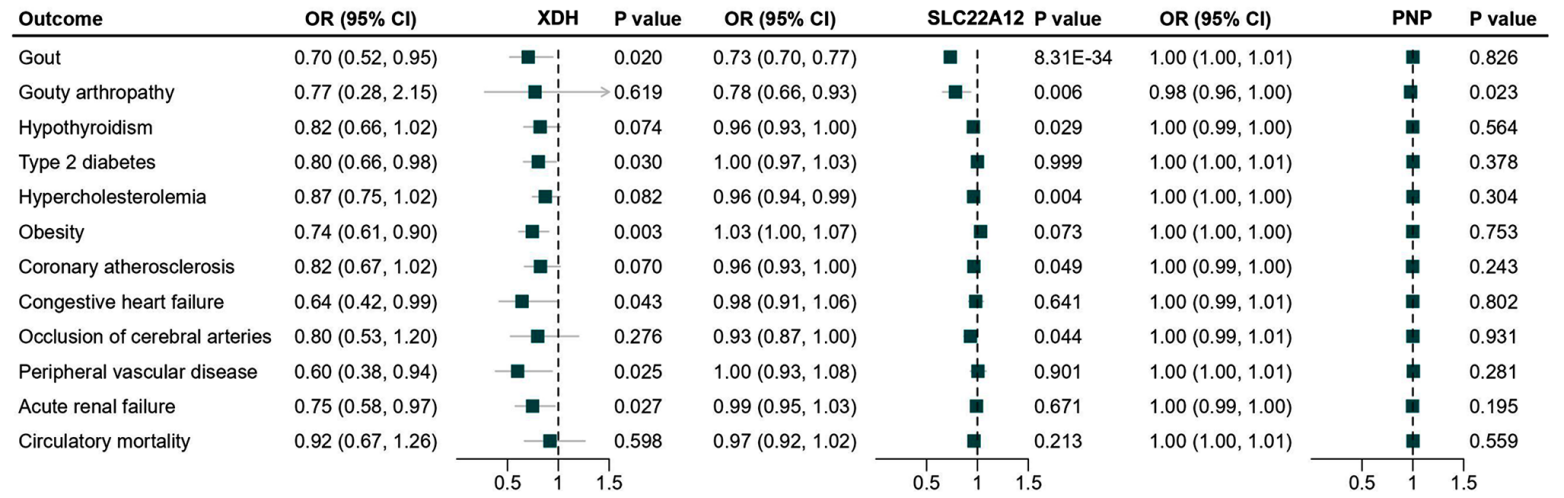
Effect of genetically predicted uric acid-lowering therapy on the risk of associated disease outcomes identified by PheWAS

The uric acid-lowering drugs as well as their target genes were shown in Supplementary Table 8. A total of 35, 87 and 50 genetic variants were selected as instruments to proxy the effects of xanthine oxidase inhibitors (XOIs), uricosuric drugs targeting gene *SLC22A12*, and purine nucleoside phosphorylase (PNP) inhibitors, respectively Supplementary Table 8). As expected, uric acid-lowering drug proxies were associated with a reduced risk of gout and/or gouty arthropathy, which serves as a positive control for the selected genetic instruments (Fig. 4). XOIs were uniquely inversely associated with type 2 diabetes (OR = 0.80, 95%CI: 0.66, 0.98, *P* = 0.030), obesity (OR = 0.74, 95%CI: 0.61, 0.90, *P* = 0.003), congestive heart failure (OR = 0.64, 95%CI: 0.42, 0.99, *P* = 0.043), peripheral vascular disease (OR = 0.60, 95%CI: 0.38, 0.94, *P* = 0.025), and acute renal failure (OR = 0.75, 95%CI: 0.58, 0.97, *P* = 0.027). Uricosuric drugs targeting the *SLC22A12* gene were associated with a reduced risk of tuberculosis (OR = 0.96, 95%CI: 0.93, 1.00, *P* = 0.032), hypothyroidism (OR = 0.96, 95%CI: 0.93, 1.00, *P* = 0.029), hypercholesterolemia (OR = 0.96, 95%CI: 0.94, 0.99, *P* = 0.004), coronary atherosclerosis (OR = 0.96, 95%CI: 0.93, 1.00, *P* = 0.047) and occlusion of cerebral arteries (OR = 0.93, 95%CI: 0.87, 1.00, *P* = 0.044). We did not identify any additional therapeutic effects for PNP inhibitors (Fig. 4). The effect estimates for all of the PheWAS-identified outcomes are displayed in Supplementary Table 10.

**Fig. 5:**
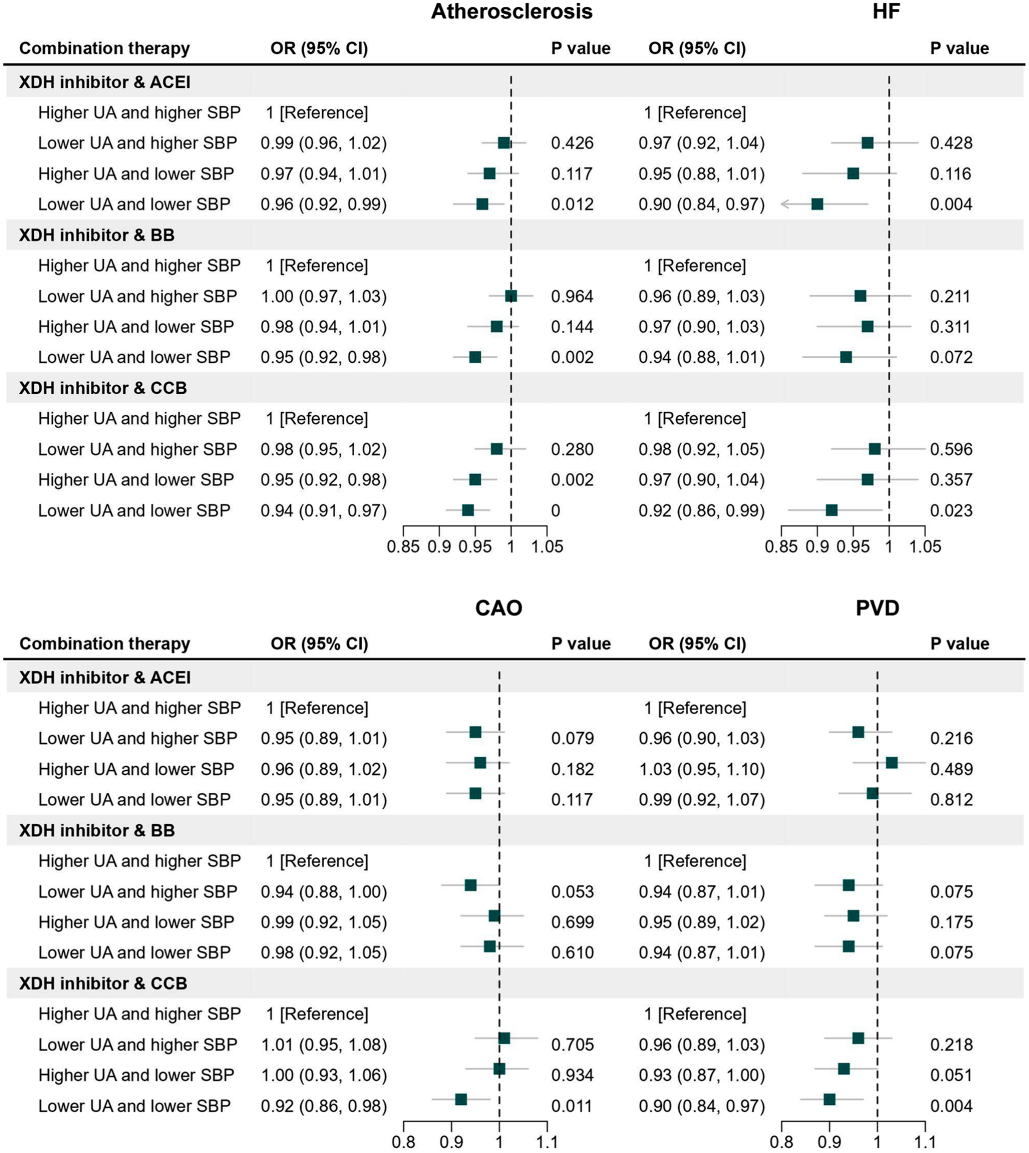
**Joint effect of combination therapy integrating XDH inhibitors and antihypertensive drugs on the risk of cardiovascular outcomes.** CAO, occlusion of cerebral arteries; HF, heart failure; PVD, peripheral vascular disease

To evaluate the joint effect of combination therapy aimed at lowering both uric acid levels and blood pressure on cardiovascular risk, we performed factorial MR analysis. The genetic instruments for antihypertensive drugs were extracted from previously publications [25, 26]. More details were presented in Supplementary Table 11. Compared to the reference group with genetically predicted higher levels of uric acid and higher blood pressure, the risk of coronary atherosclerosis, congestive heart failure, occlusion of cerebral arteries and peripheral vascular disease was lower in the group with genetically predicted lower levels of uric acid and blood pressure (Fig. 5). Especially in combination therapy integrating XOIs with calcium channel blockers (CCBs), genetically predicted lower levels of uric acid and blood pressure was associated with a 6% lower coronary atherosclerosis risk (OR = 0.94, 95%CI: 0.91, 0.97, *P* < 0.001), a 8% lower congestive heart failure risk (OR = 0.92, 95%CI: 0.86, 0.99, *P* = 0.023), a 8% lower occlusion of cerebral arteries risk (OR = 0.92, 95%CI: 0.86, 0.98, *P* = 0.011) and a 10% lower peripheral vascular disease risk (OR = 0.90, 95%CI: 0.84, 0.97, *P* = 0.004) (Supplementary Table 12).

## Discussion

In this study, we first identified totally 41 overlapping disease outcomes associated with uric acid levels using both Obs- and PRS- PheWAS approaches. Notably, the associations of uric acid with certain health outcomes such as hypothyroidism, hyperpotassemia, transient cerebral ischemia, infectious diseases (e.g., tuberculosis), inguinal hernia and benign neoplasm of the digestive system, revealed novel findings that have not been reported previously. Then, trajectory analysis revealed a primary cluster comprising predominantly cardiometabolic diseases following elevated uric acid levels. Last, drug repurposing analysis indicated that uric acid-lowering therapy may be repurposed for the treatment of cardiovascular diseases. Furthermore, we unveiled a joint effect of combination therapy that integrates uric acid-lowering and antihypertensive treatments in reducing CVD risk, suggesting that the development of compounds targeting these two markers holds significant promise for advancing CVD treatment.

Consistently, numerous observational studies have identified that elevated urate levels are associated with an increased risk of cardiovascular diseases including atrial fibrillation, [27] coronary artery disease, [28] myocardial infarction, [29] and heart failure [30]. In addition, Gill et al. provided further evidence that genetically predicted serum urate exerted a causal effect on enhancing the risk of coronary heart disease, peripheral artery disease and stroke [31]. A systematic review and meta-analysis of randomized controlled trials (RCTs) also showed a favorable effect of urate-lowering treatment on major adverse cardiovascular events (MACE) [31, 32]. All these epidemiological findings indicate the involvement of urate as a causal risk factor and a potential therapeutic target in CVD development. Aberrant serum urate may modify CVD risk through several interconnected biological mechanisms. Most importantly, uric acid can trigger inflammation and oxidative stress in vascular endothelial and smooth muscle cells, leading to endothelial dysfunction and thus increasing the risk of atherosclerosis and other cardiovascular conditions [33]. Additionally, its connection to chronic kidney disease further complicates the relationship, as renal function plays a role in urate regulation [34]. Consistently, participants with lower eGFR levels were more likely to exhibit higher uric acid levels and develop kidney diseases, however, we did not observe significant interaction between uric acid and eGFR in the identified associations, which may require further verification. Alternative theories suggest a potential link between high uric acid levels and insulin resistance, which has been recognized as a significant risk factor for cardiovascular disease [35]. However, it’s unclear whether elevated urate levels directly cause insulin resistance or both of them are a consequence of other underlying factors such as obesity and metabolic dysfunction, thus requiring more research to establish causality and to determine the extent of urate’s contribution to cardiovascular risk.

Current uric acid-lowering treatment can be divided into two main categories, reducing uric acid production with XOIs (e.g. allopurinol and febuxostat) and increasing uric acid excretion by using uricosurics that prevent reabsorption of uric acid in kidney (e.g. probenecid, sulfinpyrazone and benzbromarone). To date, the role of uric acid-lowering therapy in diseases other than hyperuricemia/gout has not been established. In seven RCTs where XOIs were compared with no-treatment or placebo, the results showed that XOIs could lower the risks of MACE and cardiovascular events (CVE) [32]. Furthermore, nine RCT studies questioned the cardiovascular safety of febuxostat, and found no differences in the risk of MACE and CVE when compared to allopurinol [32]. However, as for cardiovascular mortality, the CARES study performed a double-blind, non-inferiority RCT in 6190 patients with gout, and identified an increased risk of cardiovascular death in the febuxostat group without potential clinic follow-up [36]. While another recently published FAST study (*n* = 6128), an open-label, blinded-endpoint, non-inferiority RCT found no signals of increased death with febuxostat during the median follow-up period of 48 months [37]. But a prospective cohort study confirmed the beneficial effect of allopurinol in reducing cardiovascular mortality [38]. Consistent with previous findings, our study revealed protective effects of uric acid-lowering medications, such as XOIs, in reducing the risk of cardiovascular events. Moreover, we also investigated whether uric acid-lowering therapy had an impact on cardiovascular mortality but observed no significant association, necessitating further large-scale RCTs to validate our findings. Of note, we observed that the anti-cardiovascular effects of uricosuric agents are less effective compared to XOIs. One possible explanation is related to additional mechanisms of xanthine oxidase (XO) involved in cardiovascular development. Apart from the property of improving hyperuricemia and gout conditions that both XOIs and uricosuric agents possess, inhibiting XO can additionally decrease cardiovascular risk by reducing oxygen species production and counteracting a pro-inflammatory vascular state [39]. However, there is limited evidence supporting a direct role of uric acid transporters in the development of cardiovascular diseases.

In addition, we explored the effect of a combination therapy integrating uric acid-lowering and antihypertensive treatment for the prevention of cardiovascular diseases. As we know, the effect of antihypertensive treatment on the prevention of CVD events has been well characterized in trials [40]. However, only managing blood pressure may prove inadequate given that cardiovascular disease often involve multiple risk factors, such as high blood pressure, elevated cholesterol levels and aberrant uric acid levels [41–43]. As a result, combination therapy has become a crucial strategy in the management of cardiovascular disease, for its enhanced treatment efficacy and reduced complications and adverse events by simultaneously addressing multiple risk factors [44, 45]. We applied a factorial MR approach to simulate randomized clinical trials aimed at manipulating blood pressure and uric acid levels. The results indicated that the combination of uric acid-lowering therapy (e.g. XO inhibitors) and antihypertensive treatment (e.g. CCBs) had an additive effect in reducing the risk of cardiovascular disease. This provides evidence for conducting larger-scale clinical trials with real-world data to further investigate the combined effects in future CVD treatment.

This study has several strengths. Firstly, we performed observational PheWAS to examine short-term effects of uric acid levels on various phenotypes using uric acid measurements at the time of recruitment. Then, PRS-PheWAS provided a long-term perspective by assessing the cumulative genetic risk for elevated uric acid levels over an individual’s lifetime. Thus, the combination of results from both approaches serves as a form of validation and replication, not only enhancing statistical power and reducing bias but also facilitating a more thorough comprehension of causality. Secondly, for significant disease outcomes identified by PheWASs, we combined both national medical registry data (progress to a more advanced stage) and primary care records (early signs and symptoms of diseases) to investigate potential disease progression patterns, aiming to provide a more comprehensive and longitudinal understanding of individuals’ health trajectories. Thirdly, the factorial MR strategy offers a distinct advantage in evaluating causality between a specific drug target and the outcomes by using genetic variants as instruments. Our study also has a number of limitations. Firstly, the selected genetic instruments explained 6.06% of variance for uric acid levels, which may lead to inadequate statistical power in detecting phenotypic associations. Secondly, considering that the average age of the UK Biobank participants was 57 years at the time of ascertainment and some diseases such as neoplasms and neurological diseases tend to have a later onset, thus additional studies with a longer follow-up to identify more comorbidities are needed. Last but not least, the findings derived from genetic associations may not always generalize to diverse populations or different contexts, as variant allele frequency can vary across populations. Genetic instruments that are common across different populations or replication analyses in multiple ethnic groups are warranted to enhance the generalizability of the findings.

## Conclusions

In summary, our PheWASs revealed significant associations between uric acid levels and 41 disease outcomes, primarily cardiometabolic diseases. In addition, uric acid-lowering therapy confers protective benefits in reducing the risk of cardiovascular events. Furthermore, combining uric acid-lowering with antihypertensive drugs represents a promising avenue for future CVD treatment. Future clinical trials, whether focusing solely on uric acid levels or in combination with blood pressure management, are warranted to investigate the observed effectiveness among CVD patients.

## Electronic supplementary material

Below is the link to the electronic supplementary material.

Supplementary Material 1

Supplementary Material 2

## Declarations

### Data sharing statement

This research has been conducted using the UK Biobank Resource under Application Number 19655. Data are available from the UK Biobank (https://www.ukbiobank.ac.uk/) for researchers who meet the criteria and gain approvals to access the research database from the UK Biobank.

## References

[1] Lee SJ, Oh BK, Sung KC. Uric acid and cardiometabolic diseases. Clin Hypertens. 2020;26:13. 10.1186/s40885-020-00146-y. 32549999 10.1186/s40885-020-00146-y PMC7294650

[2] Maiuolo J, Oppedisano F, Gratteri S, Muscoli C, Mollace V. Regulation of uric acid metabolism and excretion. Int J Cardiol. 2016;213:8–14. 10.1016/j.ijcard.2015.08.109. 26316329 10.1016/j.ijcard.2015.08.109

[3] So A, Thorens B. Uric acid transport and disease. J Clin Invest. 2010;120(6):1791–9. 10.1172/JCI42344. 20516647 10.1172/JCI42344 PMC2877959

[4] Din SE, Salem UAA, Abdulazim MM. Uric acid in the pathogenesis of metabolic, renal, and cardiovascular diseases: a review. J Adv Res. 2017;8(5):537–48. 10.1016/j.jare.2016.11.004. 28748119 10.1016/j.jare.2016.11.004 PMC5512153

[5] Tana C, Ticinesi A, Prati B, Nouvenne A, Meschi T. Uric acid and cognitive function in older individuals. Nutrients. 2018;10(8). 10.3390/nu10080975. 10.3390/nu10080975 PMC6115794 30060474

[6] Bush WS, Oetjens MT, Crawford DC. Unravelling the human genome-phenome relationship using phenome-wide association studies. Nat Rev Genet. 2016;17(3):129–45. 10.1038/nrg.2015.36. 26875678 10.1038/nrg.2015.36

[7] Emmerson BT, Nagel SL, Duffy DL, Martin NG. Genetic control of the renal clearance of urate: a study of twins. Ann Rheum Dis. 1992;51(3):375–7. 10.1136/ard.51.3.375. 1575585 10.1136/ard.51.3.375 PMC1004665

[8] Wilk JB, Djousse L, Borecki I, et al. Segregation analysis of serum uric acid in the NHLBI Family Heart Study. Hum Genet. 2000;106(3):355–9. 10.1007/s004390000243. 10798367 10.1007/s004390000243

[9] Kolz M, Johnson T, Sanna S, et al. Meta-analysis of 28,141 individuals identifies common variants within five new loci that influence uric acid concentrations. PLoS Genet. 2009;5(6):e1000504. 10.1371/journal.pgen.1000504. 19503597 10.1371/journal.pgen.1000504 PMC2683940

[10] Kottgen A, Albrecht E, Teumer A, et al. Genome-wide association analyses identify 18 new loci associated with serum urate concentrations. Nat Genet. 2013;45(2):145–54. 10.1038/ng.2500. 23263486 10.1038/ng.2500 PMC3663712

[11] Tin A, Marten J, Halperin Kuhns VL, et al. Target genes, variants, tissues and transcriptional pathways influencing human serum urate levels. Nat Genet. 2019;51(10):1459–74. 10.1038/s41588-019-0504-x. 31578528 10.1038/s41588-019-0504-x PMC6858555

[12] Boocock J, Leask M, Okada Y, et al. Genomic dissection of 43 serum urate-associated loci provides multiple insights into molecular mechanisms of urate control. Hum Mol Genet. 2020;29(6):923–43. 10.1093/hmg/ddaa013. 31985003 10.1093/hmg/ddaa013

[13] Jensen AB, Moseley PL, Oprea TI, et al. Temporal disease trajectories condensed from population-wide registry data covering 6.2 million patients. Nat Commun. 2014;5:4022. 10.1038/ncomms5022. 24959948 10.1038/ncomms5022 PMC4090719

[14] Smith GD, Ebrahim S. Mendelian randomization’: can genetic epidemiology contribute to understanding environmental determinants of disease? Int J Epidemiol. 2003;32(1):1–22. 10.1093/ije/dyg070. 12689998 10.1093/ije/dyg070

[15] Ference BA, Majeed F, Penumetcha R, Flack JM, Brook RD. Effect of naturally random allocation to lower low-density lipoprotein cholesterol on the risk of coronary heart disease mediated by polymorphisms in NPC1L1, HMGCR, or both: a 2 x 2 factorial Mendelian randomization study. J Am Coll Cardiol. 2015;65(15):1552–61. 10.1016/j.jacc.2015.02.020. 25770315 10.1016/j.jacc.2015.02.020 PMC6101243

[16] Bycroft C, Freeman C, Petkova D, et al. The UK Biobank resource with deep phenotyping and genomic data. Nature. 2018;562(7726):203–9. 10.1038/s41586-018-0579-z. 30305743 10.1038/s41586-018-0579-z PMC6786975

[17] Canela-Xandri O, Rawlik K, Tenesa A. An atlas of genetic associations in UK Biobank. Nat Genet. 2018;50(11):1593–9. 10.1038/s41588-018-0248-z. 30349118 10.1038/s41588-018-0248-z PMC6707814

[18] Denny JC, Ritchie MD, Basford MA, et al. PheWAS: demonstrating the feasibility of a phenome-wide scan to discover gene-disease associations. Bioinformatics. 2010;26(9):1205–10. 10.1093/bioinformatics/btq126. 20335276 10.1093/bioinformatics/btq126 PMC2859132

[19] Carroll RJ, Bastarache L, Denny JC. R PheWAS: data analysis and plotting tools for phenome-wide association studies in the R environment. Bioinformatics. 2014;30(16):2375–6. 10.1093/bioinformatics/btu197. 24733291 10.1093/bioinformatics/btu197 PMC4133579

[20] Verma A, Bradford Y, Dudek S, et al. A simulation study investigating power estimates in phenome-wide association studies. BMC Bioinformatics. 2018;19(1):120. 10.1186/s12859-018-2135-0. 29618318 10.1186/s12859-018-2135-0 PMC5885318

[21] Wishart DS, Feunang YD, Guo AC, et al. DrugBank 5.0: a major update to the DrugBank database for 2018. Nucleic Acids Res. 2018;46(D1):D1074–82. 10.1093/nar/gkx1037. 29126136 10.1093/nar/gkx1037 PMC5753335

[22] Ference BA, Ray KK, Catapano AL, et al. Mendelian randomization study of ACLY and Cardiovascular Disease. N Engl J Med. 2019;380(11):1033–42. 10.1056/NEJMoa1806747. 30865797 10.1056/NEJMoa1806747 PMC7612927

[23] Desquilbet L, Mariotti F. Dose-response analyses using restricted cubic spline functions in public health research. Stat Med. 2010;29(9):1037–57. 10.1002/sim.3841. 20087875 10.1002/sim.3841

[24] Defesche JC, Gidding SS, Harada-Shiba M, Hegele RA, Santos RD, Wierzbicki AS. Familial hypercholesterolaemia. Nat Rev Dis Primers. 2017;3:17093. 10.1038/nrdp.2017.93. 29219151 10.1038/nrdp.2017.93

[25] Gill D, Georgakis MK, Koskeridis F, et al. Use of genetic variants related to antihypertensive drugs to inform on Efficacy and Side effects. Circulation. 2019;140(4):270–9. 10.1161/CIRCULATIONAHA.118.038814. 31234639 10.1161/CIRCULATIONAHA.118.038814 PMC6687408

[26] Evangelou E, Warren HR, Mosen-Ansorena D, et al. Publisher correction: genetic analysis of over 1 million people identifies 535 new loci associated with blood pressure traits. Nat Genet. 2018;50(12):1755. 10.1038/s41588-018-0297-3. 30429575 10.1038/s41588-018-0297-3

[27] Tamariz L, Hernandez F, Bush A, Palacio A, Hare JM. Association between serum uric acid and atrial fibrillation: a systematic review and meta-analysis. Heart Rhythm. 2014;11(7):1102–8. 10.1016/j.hrthm.2014.04.003. 24709288 10.1016/j.hrthm.2014.04.003

[28] Zuo T, Liu X, Jiang L, Mao S, Yin X, Guo L. Hyperuricemia and coronary heart disease mortality: a meta-analysis of prospective cohort studies. BMC Cardiovasc Disord. 2016;16(1):207. 10.1186/s12872-016-0379-z. 27793095 10.1186/s12872-016-0379-z PMC5084405

[29] Kojima S, Sakamoto T, Ishihara M, et al. Prognostic usefulness of serum uric acid after acute myocardial infarction (the Japanese Acute Coronary Syndrome Study). Am J Cardiol. 2005;96(4):489–95. 10.1016/j.amjcard.2005.04.007. 16098298 10.1016/j.amjcard.2005.04.007

[30] Huang H, Huang B, Li Y, et al. Uric acid and risk of heart failure: a systematic review and meta-analysis. Eur J Heart Fail. 2014;16(1):15–24. 10.1093/eurjhf/hft132. 23933579 10.1093/eurjhf/hft132

[31] Gill D, Cameron AC, Burgess S, et al. Urate, blood pressure, and Cardiovascular Disease: evidence from mendelian randomization and Meta-analysis of clinical trials. Hypertension. 2021;77(2):383–92. 10.1161/HYPERTENSIONAHA.120.16547. 33356394 10.1161/HYPERTENSIONAHA.120.16547 PMC7803439

[32] Zhao L, Cao L, Zhao TY, et al. Cardiovascular events in hyperuricemia population and a cardiovascular benefit-risk assessment of urate-lowering therapies: a systematic review and meta-analysis. Chin Med J (Engl). 2020;133(8):982–93. 10.1097/CM9.0000000000000682. 32106120 10.1097/CM9.0000000000000682 PMC7176444

[33] Yu W, Cheng JD. Uric Acid and Cardiovascular Disease: an update from molecular mechanism to clinical perspective. Front Pharmacol. 2020;11:582680. 10.3389/fphar.2020.582680. 33304270 10.3389/fphar.2020.582680 PMC7701250

[34] Borghi C, Agabiti-Rosei E, Johnson RJ, et al. Hyperuricaemia and gout in cardiovascular, metabolic and kidney disease. Eur J Intern Med. 2020;80:1–11. 10.1016/j.ejim.2020.07.006. 32739239 10.1016/j.ejim.2020.07.006

[35] Lu J, He Y, Cui L, et al. Hyperuricemia predisposes to the Onset of Diabetes via promoting pancreatic beta-cell death in uricase-deficient male mice. Diabetes. 2020;69(6):1149–63. 10.2337/db19-0704. 32312870 10.2337/db19-0704 PMC7243290

[36] White WB, Saag KG, Becker MA, et al. Cardiovascular Safety of Febuxostat or Allopurinol in patients with gout. N Engl J Med. 2018;378(13):1200–10. 10.1056/NEJMoa1710895. 29527974 10.1056/NEJMoa1710895

[37] Mackenzie IS, Ford I, Nuki G, et al. Long-term cardiovascular safety of febuxostat compared with allopurinol in patients with gout (FAST): a multicentre, prospective, randomised, open-label, non-inferiority trial. Lancet. 2020;396(10264):1745–57. 10.1016/S0140-6736(20)32234-0. 33181081 10.1016/S0140-6736(20)32234-0

[38] Perez Ruiz F, Richette P, Stack AG, Karra Gurunath R, Garcia de Yebenes MJ, Carmona L. Failure to reach uric acid target of < 0.36 mmol/L in hyperuricaemia of gout is associated with elevated total and cardiovascular mortality. RMD Open. 2019;5(2):e001015. 10.1136/rmdopen-2019-001015. 31673414 10.1136/rmdopen-2019-001015 PMC6803010

[39] Tanaka A, Node K. Xanthine oxidase inhibition for cardiovascular disease prevention. Lancet. 2022;400(10359):1172–3. 10.1016/S0140-6736(22)01778-0. 36215992 10.1016/S0140-6736(22)01778-0

[40] Thompson AM, Hu T, Eshelbrenner CL, Reynolds K, He J, Bazzano LA. Antihypertensive treatment and secondary prevention of cardiovascular disease events among persons without hypertension: a meta-analysis. JAMA. 2011;305(9):913–22. 10.1001/jama.2011.250. 21364140 10.1001/jama.2011.250 PMC4313888

[41] Vasan RS, Larson MG, Leip EP, et al. Impact of high-normal blood pressure on the risk of cardiovascular disease. N Engl J Med. 2001;345(18):1291–7. 10.1056/NEJMoa003417. 11794147 10.1056/NEJMoa003417

[42] Michos ED, McEvoy JW, Blumenthal RS. Lipid management for the Prevention of Atherosclerotic Cardiovascular Disease. N Engl J Med. 2019;381(16):1557–67. 10.1056/NEJMra1806939. 31618541 10.1056/NEJMra1806939

[43] Li X, Meng X, Timofeeva M, et al. Serum uric acid levels and multiple health outcomes: umbrella review of evidence from observational studies, randomised controlled trials, and mendelian randomisation studies. BMJ. 2017;357:j2376. 10.1136/bmj.j2376. 28592419 10.1136/bmj.j2376 PMC5461476

[44] Working Group on the Summit on Combination Therapy for CVD, Yusuf S, Attaran A, et al. Combination pharmacotherapy to prevent cardiovascular disease: present status and challenges. Eur Heart J. 2014;35(6):353–64. 10.1093/eurheartj/eht407. 24288261 10.1093/eurheartj/eht407

[45] Huffman MD, Xavier D, Perel P. Uses of polypills for cardiovascular disease and evidence to date. Lancet. 2017;389(10073):1055–65. 10.1016/S0140-6736(17)30553-6. 28290995 10.1016/S0140-6736(17)30553-6

