# Supplementary material for: A phenome-wide association and factorial Mendelian randomization study on the repurposing of uric acid-lowering drugs for cardiovascular outcomes
Source: Eur J Epidemiol. 2024 Jul 11;39(8):869–80. doi: 10.1007/s10654-024-01138-0 (PMC11410910; doi:10.1007/s10654-024-01138-0)
Supplement: Supplementary file 1 — Supplementary Material 1 [file 10654_2024_1138_MOESM1_ESM.docx]

**Supplementary Materials**

**Supplementary Figures**


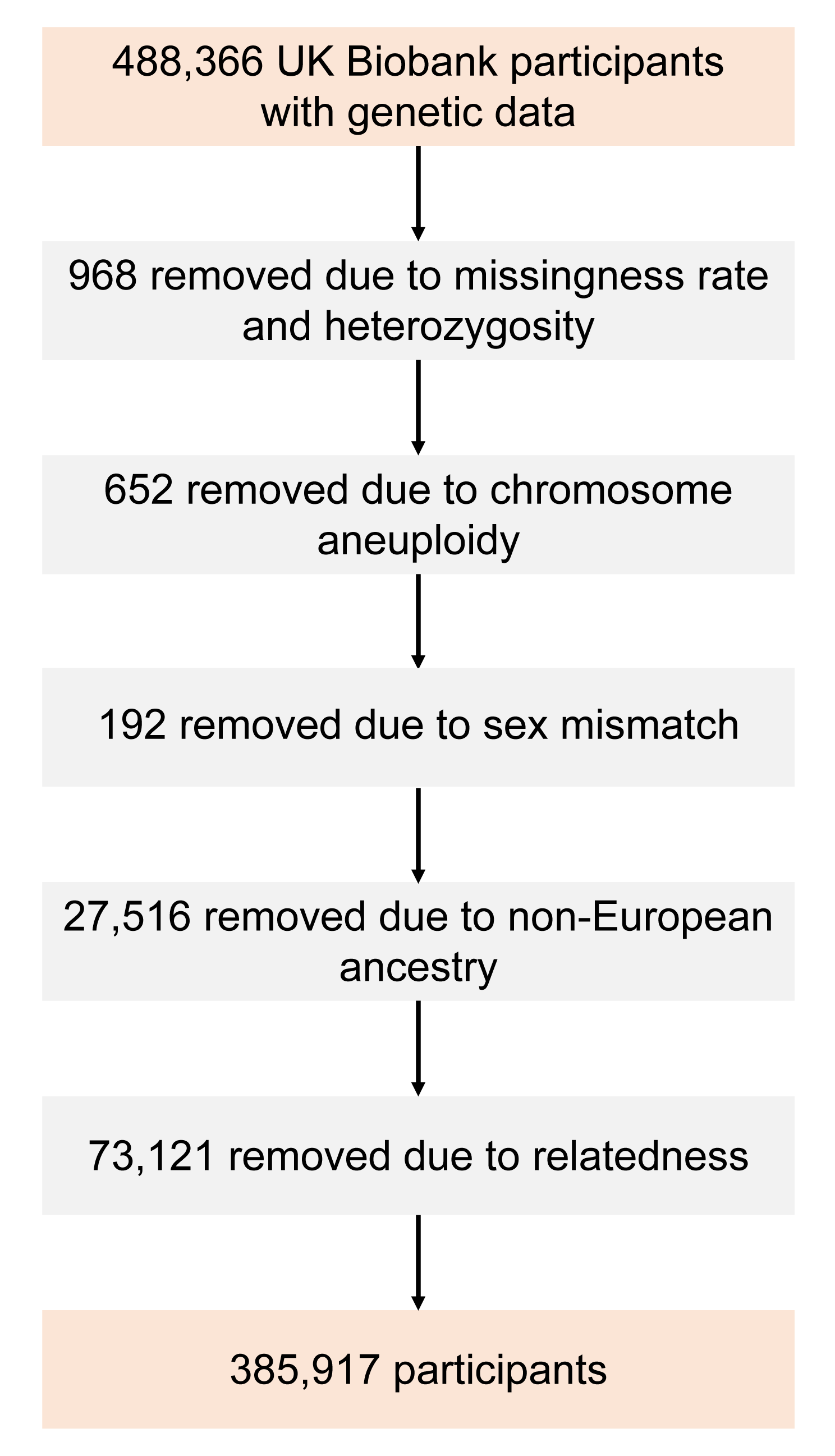


**Supplementary Figure 1. Flowchart of sample quality control in the UK Biobank.**


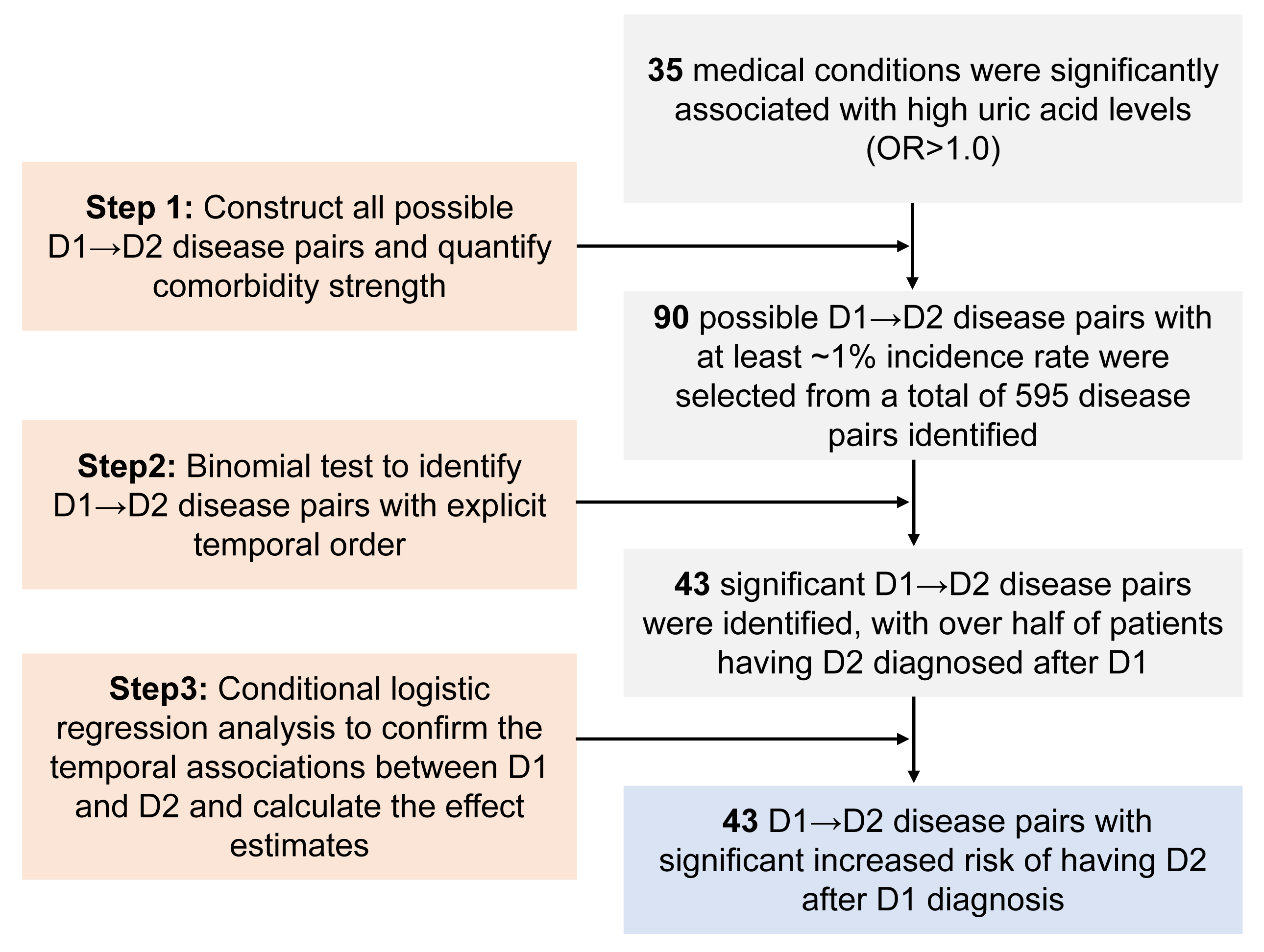


**Supplementary Figure 2. Flowchart of disease trajectory analysis.**

**Supplementary Tables**

**Supplementary Table 1. Genetic instruments used to proxy serum uric acid levels.**

| **SNP** | **Chr** | **Position (b37)** | **EA** | **NEA** | **EAF** | **Beta** | **SE** | **P value** | **N** | **R2 (%)** | **Gene** |
| --- | --- | --- | --- | --- | --- | --- | --- | --- | --- | --- | --- |
| rs2480712 | 1 | 2156999 | C | G | 0.66 | 0.024 | 0.004 | 6.25E-09 | 288649 | 0.012 | *SKI* |
| rs4646068 | 1 | 15828704 | T | C | 0.69 | 0.024 | 0.004 | 7.71E-09 | 288649 | 0.012 | *CASP9* |
| rs79598313 | 1 | 27284913 | T | C | 0.03 | 0.100 | 0.013 | 9.22E-15 | 288649 | 0.020 | *KDF1* |
| rs141990161 | 1 | 119943525 | T | C | 0.98 | 0.133 | 0.023 | 1.59E-08 | 288649 | 0.012 | *HAO2* |
| rs10910845 | 1 | 145723120 | A | C | 0.47 | 0.058 | 0.004 | 1.50E-51 | 288649 | 0.073 | *NBPF20* |
| rs11204701 | 1 | 150662179 | A | T | 0.78 | -0.036 | 0.005 | 1.04E-14 | 288649 | 0.018 | *GOLPH3L* |
| rs2070803 | 1 | 155157715 | A | G | 0.58 | 0.053 | 0.004 | 4.09E-41 | 288649 | 0.061 | *TRIM46* |
| rs12134456 | 1 | 155722506 | C | G | 0.63 | -0.043 | 0.005 | 7.61E-19 | 288649 | 0.026 | *GON4L* |
| rs2760215 | 1 | 163675883 | T | C | 0.50 | -0.025 | 0.004 | 5.81E-11 | 288649 | 0.014 | *LOC100422212* |
| rs12037861 | 1 | 221038177 | A | T | 0.70 | 0.023 | 0.004 | 3.39E-08 | 288649 | 0.011 | *HLX-AS1* |
| rs2867112 | 2 | 651349 | T | G | 0.83 | 0.035 | 0.005 | 9.84E-12 | 288649 | 0.017 | *TMEM18* |
| rs72782806 | 2 | 15788511 | A | G | 0.26 | 0.025 | 0.004 | 8.12E-09 | 288649 | 0.014 | *DDX1* |
| rs72804854 | 2 | 27159850 | A | G | 0.96 | -0.056 | 0.010 | 4.97E-08 | 288649 | 0.011 | *DPYSL5* |
| rs1260326 | 2 | 27730940 | T | C | 0.40 | 0.070 | 0.004 | 4.61E-69 | 288649 | 0.106 | *GCKR* |
| rs62140395 | 2 | 28244926 | C | G | 0.12 | 0.052 | 0.007 | 3.17E-15 | 288649 | 0.019 | *BABAM2* |
| rs12472381 | 2 | 59321225 | A | G | 0.39 | 0.022 | 0.004 | 1.80E-08 | 288649 | 0.010 | *LINC01122* |
| rs12987661 | 2 | 69813458 | T | C | 0.87 | 0.041 | 0.006 | 1.44E-12 | 288649 | 0.016 | *AAK1* |
| rs759219 | 2 | 71163225 | T | C | 0.44 | -0.022 | 0.004 | 7.93E-09 | 288649 | 0.010 | *ATP6V1B1* |
| rs17050272 | 2 | 121306440 | A | G | 0.42 | 0.032 | 0.004 | 1.57E-15 | 288649 | 0.022 | *LINC01101* |
| rs11683692 | 2 | 145509615 | T | C | 0.94 | -0.048 | 0.008 | 1.32E-08 | 288649 | 0.012 | *TEX41* |
| rs1234413 | 2 | 148844369 | T | C | 0.44 | -0.022 | 0.004 | 7.08E-09 | 288649 | 0.010 | *MBD5* |
| rs9287911 | 2 | 170037294 | A | T | 0.25 | 0.038 | 0.004 | 1.13E-17 | 288649 | 0.031 | *LRP2* |
| rs187355703 | 2 | 176993583 | C | G | 0.97 | -0.086 | 0.013 | 2.70E-11 | 288649 | 0.015 | *HOXD8* |
| rs1047891 | 2 | 211540507 | A | C | 0.31 | -0.024 | 0.004 | 2.09E-08 | 288649 | 0.012 | *CPS1* |
| rs9288447 | 2 | 213083638 | T | C | 0.55 | -0.023 | 0.004 | 3.27E-09 | 288649 | 0.011 | *ERBB4* |
| rs699465 | 3 | 52310442 | A | G | 0.14 | 0.045 | 0.006 | 6.45E-15 | 288649 | 0.019 | *WDR82* |
| rs2581817 | 3 | 53071797 | C | G | 0.42 | 0.048 | 0.004 | 4.87E-35 | 288649 | 0.050 | *SFMBT1* |
| rs11128111 | 3 | 69145632 | T | C | 0.48 | -0.021 | 0.004 | 4.64E-08 | 288649 | 0.010 | *ARL6IP5* |
| rs7640441 | 3 | 125118082 | A | C | 0.25 | -0.028 | 0.005 | 1.26E-09 | 288649 | 0.011 | *ZNF148* |
| rs11718633 | 3 | 126012421 | T | C | 0.20 | -0.028 | 0.005 | 7.05E-09 | 288649 | 0.011 | *KLF15* |
| rs80120242 | 3 | 132235344 | A | T | 0.95 | -0.062 | 0.010 | 1.87E-09 | 288649 | 0.013 | *DNAJC13* |
| rs62294340 | 3 | 169155476 | A | G | 0.36 | -0.022 | 0.004 | 5.00E-08 | 288649 | 0.010 | *MECOM* |
| rs2030869 | 4 | 9419763 | A | T | 0.98 | 0.215 | 0.027 | 3.64E-15 | 288649 | 0.022 | *DEFB131A* |
| rs4447862 | 4 | 9931645 | C | G | 0.73 | 0.330 | 0.004 | 0.00E+00 | 288649 | 2.304 | *SLC2A9* |
| rs2108878 | 4 | 10527342 | T | C | 0.70 | 0.107 | 0.004 | 7.54E-143 | 288649 | 0.247 | *CLNK* |
| rs98270 | 4 | 48019323 | A | G | 0.36 | 0.022 | 0.004 | 4.21E-08 | 288649 | 0.010 | *NIPAL1* |
| rs10857147 | 4 | 81181072 | A | T | 0.71 | 0.024 | 0.004 | 2.21E-08 | 288649 | 0.012 | *FGF5* |
| rs116183010 | 4 | 88468158 | A | G | 0.02 | 0.093 | 0.014 | 2.14E-11 | 288649 | 0.015 | *SPARCL1* |
| rs74904971 | 4 | 89050026 | A | C | 0.11 | 0.254 | 0.006 | 0.00E+00 | 288649 | 0.617 | *ABCG2* |
| rs2704571 | 4 | 89960285 | A | G | 0.32 | 0.023 | 0.004 | 1.35E-08 | 288649 | 0.011 | *FAM13A* |
| rs12644329 | 4 | 143634746 | A | G | 0.62 | -0.023 | 0.004 | 8.86E-09 | 288649 | 0.011 | *INPP4B* |
| rs1440411 | 4 | 144158285 | T | C | 0.57 | -0.028 | 0.004 | 1.08E-12 | 288649 | 0.017 | *USP38* |
| rs455213 | 5 | 34660235 | T | C | 0.54 | -0.027 | 0.004 | 6.05E-12 | 288649 | 0.016 | *RAI14* |
| rs10942549 | 5 | 72426137 | C | G | 0.31 | -0.042 | 0.004 | 1.64E-22 | 288649 | 0.038 | *TMEM171* |
| rs76004499 | 5 | 176705865 | C | G | 0.97 | -0.074 | 0.013 | 3.26E-08 | 288649 | 0.011 | *NSD1* |
| rs12530084 | 6 | 7214676 | T | C | 0.22 | 0.066 | 0.005 | 9.55E-48 | 288649 | 0.060 | *RREB1* |
| rs138739848 | 6 | 25250492 | T | C | 0.05 | 0.062 | 0.009 | 1.30E-11 | 288649 | 0.016 | *LOC101928663* |
| rs1359232 | 6 | 25809716 | A | C | 0.47 | -0.091 | 0.004 | 4.12E-126 | 288649 | 0.179 | *SLC17A1* |
| rs35942569 | 6 | 26339131 | A | G | 0.92 | -0.067 | 0.007 | 3.51E-20 | 288649 | 0.032 | *BTN3A2* |
| rs66975207 | 6 | 26942146 | A | C | 0.92 | -0.057 | 0.007 | 6.23E-15 | 288649 | 0.023 | *LINC00240* |
| rs35848276 | 6 | 27521096 | T | C | 0.09 | 0.053 | 0.007 | 8.92E-14 | 288649 | 0.020 | *ZNF184* |
| rs68188794 | 6 | 28080777 | T | C | 0.92 | -0.059 | 0.008 | 2.72E-14 | 288649 | 0.019 | *ZSCAN16-AS1* |
| rs4713177 | 6 | 28673998 | A | G | 0.11 | 0.051 | 0.007 | 3.99E-13 | 288649 | 0.018 | *LINC00533* |
| rs429479 | 6 | 29372323 | A | G | 0.90 | -0.048 | 0.007 | 2.23E-12 | 288649 | 0.016 | *OR12D2* |
| rs742493 | 6 | 40998167 | T | C | 0.88 | 0.039 | 0.006 | 9.79E-11 | 288649 | 0.015 | *UNC5CL* |
| rs1574430 | 6 | 43269029 | A | C | 0.41 | 0.029 | 0.004 | 2.77E-14 | 288649 | 0.018 | *SLC22A7* |
| rs10223666 | 6 | 43805502 | C | G | 0.70 | 0.046 | 0.004 | 6.62E-28 | 288649 | 0.046 | *VEGFA* |
| rs4897160 | 6 | 126223944 | A | G | 0.48 | 0.030 | 0.004 | 1.96E-14 | 288649 | 0.019 | *NCOA7* |
| rs62435145 | 7 | 1286567 | T | G | 0.69 | 0.042 | 0.005 | 2.36E-16 | 288649 | 0.024 | *UNCX* |
| rs13226650 | 7 | 73017005 | A | G | 0.81 | 0.049 | 0.005 | 1.34E-23 | 288649 | 0.033 | *MLXIPL* |
| rs11551890 | 7 | 97845713 | A | G | 0.51 | 0.023 | 0.004 | 2.40E-08 | 288649 | 0.011 | *TECPR1* |
| rs10480300 | 7 | 151406005 | T | C | 0.28 | 0.030 | 0.004 | 4.26E-12 | 288649 | 0.019 | *PRKAG2* |
| rs34861762 | 8 | 23748420 | T | C | 0.42 | 0.034 | 0.004 | 3.50E-19 | 288649 | 0.025 | *STC1* |
| rs2466077 | 8 | 32432753 | T | G | 0.53 | -0.022 | 0.004 | 1.78E-08 | 288649 | 0.010 | *NRG1* |
| rs2943539 | 8 | 76479839 | T | C | 0.48 | 0.041 | 0.004 | 6.42E-28 | 288649 | 0.036 | *HNF4G* |
| rs62517932 | 8 | 77031593 | A | G | 0.08 | 0.045 | 0.007 | 3.80E-10 | 288649 | 0.014 | *LINC01111* |
| rs10956924 | 8 | 95678312 | T | C | 0.28 | -0.024 | 0.004 | 1.79E-08 | 288649 | 0.012 | *ESRP1* |
| rs10971420 | 9 | 33125000 | T | C | 0.69 | 0.031 | 0.004 | 4.14E-14 | 288649 | 0.021 | *B4GALT1* |
| rs56106601 | 9 | 130770484 | A | C | 0.95 | 0.061 | 0.009 | 2.67E-11 | 288649 | 0.016 | *FAM102A* |
| rs74440730 | 10 | 16920892 | A | C | 0.89 | -0.037 | 0.006 | 2.22E-09 | 288649 | 0.013 | *CUBN* |
| rs10994860 | 10 | 52645424 | T | C | 0.18 | 0.064 | 0.005 | 1.31E-36 | 288649 | 0.057 | *A1CF* |
| rs1649078 | 10 | 60293320 | A | C | 0.48 | -0.039 | 0.004 | 4.08E-19 | 288649 | 0.033 | *BICC1* |
| rs1171617 | 10 | 61467182 | T | G | 0.77 | 0.079 | 0.005 | 1.81E-66 | 288649 | 0.086 | *SLC16A9* |
| rs9420446 | 10 | 88880689 | T | C | 0.14 | -0.038 | 0.006 | 1.13E-11 | 288649 | 0.014 | *FAM35A* |
| rs35198068 | 10 | 114754784 | T | C | 0.71 | 0.025 | 0.004 | 5.85E-09 | 288649 | 0.014 | *TCF7L2* |
| rs35506085 | 11 | 2165576 | A | G | 0.19 | -0.029 | 0.005 | 1.50E-08 | 288649 | 0.012 | *IGF2* |
| rs3925584 | 11 | 30760335 | T | C | 0.55 | 0.030 | 0.004 | 1.66E-15 | 288649 | 0.019 | *DCDC1* |
| rs71456318 | 11 | 64332862 | A | C | 0.48 | 0.079 | 0.004 | 4.41E-92 | 288649 | 0.135 | *SLC22A11* |
| rs10896028 | 11 | 65432187 | A | T | 0.65 | -0.048 | 0.004 | 4.10E-33 | 288649 | 0.050 | *RELA* |
| rs3862387 | 11 | 65939921 | T | G | 0.87 | -0.033 | 0.006 | 3.74E-09 | 288649 | 0.010 | *PACS1* |
| rs10892354 | 11 | 119238381 | T | C | 0.38 | 0.030 | 0.004 | 2.60E-13 | 288649 | 0.019 | *USP2* |
| rs7303595 | 12 | 15359063 | A | T | 0.34 | 0.025 | 0.004 | 7.05E-10 | 288649 | 0.014 | *RERG* |
| rs7315236 | 12 | 52251933 | T | C | 0.36 | 0.029 | 0.004 | 1.91E-13 | 288649 | 0.018 | *LOC105369971* |
| rs12313306 | 12 | 57751854 | T | C | 0.25 | -0.076 | 0.004 | 6.74E-65 | 288649 | 0.125 | *R3HDM2* |
| rs12368865 | 12 | 58422642 | A | G | 0.91 | 0.046 | 0.007 | 1.32E-11 | 288649 | 0.015 | *LINC02403* |
| rs10774625 | 12 | 111910219 | A | G | 0.48 | 0.032 | 0.004 | 5.54E-17 | 288649 | 0.022 | *ATXN2* |
| rs17696736 | 12 | 112486818 | A | G | 0.57 | -0.028 | 0.004 | 1.76E-12 | 288649 | 0.017 | *NAA25* |
| rs1800574 | 12 | 121416864 | T | C | 0.03 | -0.081 | 0.012 | 2.84E-12 | 288649 | 0.016 | *HNF1A* |
| rs28530689 | 12 | 122500748 | A | C | 0.51 | 0.032 | 0.004 | 1.27E-16 | 288649 | 0.022 | *LOC100506691* |
| rs12423664 | 12 | 133069894 | A | G | 0.15 | 0.042 | 0.006 | 1.75E-13 | 288649 | 0.017 | *FBRSL1* |
| rs7986094 | 13 | 31029931 | A | C | 0.30 | -0.024 | 0.004 | 1.74E-08 | 288649 | 0.012 | *HMGB1* |
| rs626277 | 13 | 72347696 | A | C | 0.59 | 0.026 | 0.004 | 2.69E-11 | 288649 | 0.015 | *DACH1* |
| rs861536 | 14 | 104167564 | A | G | 0.62 | 0.024 | 0.004 | 2.16E-09 | 288649 | 0.012 | *KLC1* |
| rs1478604 | 15 | 39873321 | T | C | 0.71 | -0.026 | 0.004 | 4.49E-10 | 288649 | 0.015 | *THBS1* |
| rs2929508 | 15 | 72246964 | A | T | 0.26 | -0.029 | 0.005 | 3.65E-09 | 288649 | 0.012 | *MYO9A* |
| rs8040109 | 15 | 73334225 | A | C | 0.71 | 0.025 | 0.004 | 5.85E-09 | 288649 | 0.014 | *NEO1* |
| rs2472297 | 15 | 75027880 | T | C | 0.25 | -0.028 | 0.005 | 1.50E-08 | 288649 | 0.011 | *CYP1A1* |
| rs10851885 | 15 | 76304503 | A | G | 0.76 | -0.054 | 0.005 | 4.16E-32 | 288649 | 0.040 | *NRG4* |
| rs157768 | 15 | 76833779 | T | C | 0.09 | -0.040 | 0.007 | 7.96E-09 | 288649 | 0.011 | *SCAPER* |
| rs55781567 | 15 | 78857986 | C | G | 0.65 | 0.023 | 0.004 | 1.10E-08 | 288649 | 0.011 | *CHRNA5* |
| rs12908437 | 15 | 99287375 | T | C | 0.38 | 0.046 | 0.004 | 1.56E-30 | 288649 | 0.046 | *IGF1R* |
| rs4997081 | 16 | 20365234 | C | G | 0.20 | -0.030 | 0.005 | 4.18E-10 | 288649 | 0.012 | *UMOD* |
| rs8050136 | 16 | 53816275 | A | C | 0.40 | 0.025 | 0.004 | 2.34E-10 | 288649 | 0.014 | *FTO* |
| rs62052820 | 16 | 69575238 | A | G | 0.21 | 0.041 | 0.005 | 2.81E-18 | 288649 | 0.023 | *MIR1538* |
| rs4788815 | 16 | 71634811 | A | T | 0.36 | -0.026 | 0.004 | 7.44E-11 | 288649 | 0.015 | *TAT* |
| rs9302635 | 16 | 72144174 | T | C | 0.81 | -0.028 | 0.005 | 8.07E-09 | 288649 | 0.011 | *DHX38* |
| rs57652769 | 16 | 79753976 | T | C | 0.31 | -0.036 | 0.004 | 8.56E-18 | 288649 | 0.028 | *MAFTRR* |
| rs11644696 | 16 | 81572093 | A | G | 0.48 | 0.022 | 0.004 | 1.41E-08 | 288649 | 0.010 | *CMIP* |
| rs2453580 | 17 | 19438321 | T | C | 0.60 | 0.025 | 0.004 | 7.01E-10 | 288649 | 0.014 | *SLC47A1* |
| rs3794748 | 17 | 53365172 | A | G | 0.41 | 0.038 | 0.004 | 1.38E-21 | 288649 | 0.031 | *HLF* |
| rs9895661 | 17 | 59456589 | T | C | 0.82 | 0.050 | 0.005 | 7.23E-23 | 288649 | 0.035 | *BCAS3* |
| rs11663816 | 18 | 57876227 | T | C | 0.73 | -0.030 | 0.004 | 1.40E-12 | 288649 | 0.019 | *MC4R* |
| rs57070985 | 19 | 4969053 | A | G | 0.65 | 0.029 | 0.004 | 2.04E-12 | 288649 | 0.018 | *KDM4B* |
| rs10405423 | 19 | 7211311 | A | C | 0.66 | 0.039 | 0.004 | 1.07E-20 | 288649 | 0.033 | *INSR* |
| rs4808762 | 19 | 18326222 | T | C | 0.72 | -0.024 | 0.004 | 1.36E-08 | 288649 | 0.012 | *PDE4C* |
| rs2868194 | 19 | 33350060 | T | C | 0.41 | -0.027 | 0.004 | 8.90E-12 | 288649 | 0.016 | *SLC7A9* |
| rs35396326 | 19 | 45357003 | C | G | 0.70 | 0.025 | 0.004 | 2.30E-08 | 288649 | 0.014 | *NECTIN2* |
| rs62128132 | 19 | 50217955 | T | C | 0.97 | -0.118 | 0.015 | 1.32E-15 | 288649 | 0.021 | *CPT1C* |
| rs7267595 | 20 | 10643850 | A | C | 0.51 | 0.023 | 0.004 | 3.15E-09 | 288649 | 0.011 | *JAG1* |
| rs6119510 | 20 | 33287782 | T | G | 0.60 | -0.023 | 0.004 | 3.20E-09 | 288649 | 0.011 | *TP53INP2* |
| rs1800961 | 20 | 43042364 | T | C | 0.03 | -0.076 | 0.012 | 1.63E-10 | 288649 | 0.014 | *HNF4A* |
| rs219781 | 21 | 37832621 | T | G | 0.25 | -0.025 | 0.004 | 1.56E-08 | 288649 | 0.014 | *CLDN14* |
| rs12485100 | 22 | 44325516 | T | G | 0.17 | -0.033 | 0.005 | 2.44E-10 | 288649 | 0.015 | *PNPLA3* |

**Supplementary Table 2. Significant phenotypic associations identified by observational PheWAS.**

| **Phenotype** | **Description** | **Group** | **Cases** | **Participants** | **OR (95% CI)** | **P value** | **FDR** |
| --- | --- | --- | --- | --- | --- | --- | --- |
| 274.1 | Gout | endocrine/metabolic | 9778 | 501378 | 1.13 (1.13-1.13) | 0 | 0 |
| 585.3 | Chronic renal failure [CKD] | genitourinary | 11545 | 474357 | 1.07 (1.07-1.07) | 0 | 0 |
| 585.1 | Acute renal failure | genitourinary | 24844 | 487656 | 1.03 (1.03-1.03) | 8.93E-214 | 3.14E-211 |
| 585.31 | Renal dialysis | genitourinary | 1037 | 463849 | 1.13 (1.12-1.14) | 5.47E-208 | 1.44E-205 |
| 401.1 | Essential hypertension | circulatory system | 116816 | 501028 | 1.02 (1.02-1.02) | 2.11E-203 | 4.45E-201 |
| 401.22 | Hypertensive chronic kidney disease | circulatory system | 1373 | 385585 | 1.11 (1.10-1.11) | 1.88E-197 | 3.31E-195 |
| 274.11 | Gouty arthropathy | endocrine/metabolic | 319 | 491919 | 1.18 (1.17-1.19) | 1.42E-160 | 2.14E-158 |
| 580.14 | Chronic glomerulonephritis, NOS | genitourinary | 986 | 463798 | 1.11 (1.10-1.12) | 3.71E-154 | 4.89E-152 |
| 587 | Kidney replaced by transpant | genitourinary | 388 | 463200 | 1.16 (1.14-1.17) | 3.21E-127 | 3.76E-125 |
| 285.2 | Anemia of chronic disease | hematopoietic | 813 | 460683 | 1.10 (1.09-1.11) | 1.45E-105 | 1.53E-103 |
| 317 | Alcohol-related disorders | mental disorders | 8831 | 463849 | 1.03 (1.03-1.04) | 1.79E-99 | 1.72E-97 |
| 428.2 | Heart failure NOS | circulatory system | 14885 | 497164 | 1.02 (1.02-1.03) | 7.14E-87 | 5.79E-85 |
| 585.2 | Renal failure NOS | genitourinary | 2050 | 464862 | 1.06 (1.06-1.07) | 1.21E-86 | 9.12E-85 |
| 276.13 | Hyperpotassemia | endocrine/metabolic | 4281 | 474382 | 1.04 (1.04-1.05) | 8.76E-81 | 5.78E-79 |
| 252.1 | Hyperparathyroidism | endocrine/metabolic | 2102 | 495279 | 1.06 (1.05-1.07) | 2.36E-80 | 1.46E-78 |
| 272.11 | Hypercholesterolemia | endocrine/metabolic | 58929 | 495199 | 1.01 (1.01-1.01) | 2.39E-69 | 1.40E-67 |
| 428.1 | Congestive heart failure (CHF) NOS | circulatory system | 8136 | 490415 | 1.03 (1.03-1.03) | 2.40E-68 | 1.33E-66 |
| 250.2 | Type 2 diabetes | endocrine/metabolic | 35923 | 496277 | 1.01 (1.01-1.02) | 3.63E-65 | 1.91E-63 |
| 571.5 | Other chronic nonalcoholic liver disease | digestive | 7126 | 484554 | 1.03 (1.03-1.03) | 3.34E-59 | 1.68E-57 |
| 580.2 | Nephrotic syndrome without mention of glomerulonephritis | genitourinary | 1153 | 463965 | 1.07 (1.06-1.07) | 5.20E-54 | 2.49E-52 |
| 411.8 | Other chronic ischemic heart disease, unspecified | circulatory system | 29554 | 471928 | 1.01 (1.01-1.02) | 6.66E-52 | 3.05E-50 |
| 411.2 | Myocardial infarction | circulatory system | 24152 | 466526 | 1.02 (1.01-1.02) | 5.46E-50 | 2.40E-48 |
| 306 | Other mental disorder | mental disorders | 74908 | 481396 | 1.01 (1.01-1.01) | 6.75E-50 | 2.85E-48 |
| 586.11 | Small kidney | genitourinary | 537 | 463349 | 1.09 (1.08-1.10) | 3.10E-49 | 1.26E-47 |
| 276.6 | Fluid overload | endocrine/metabolic | 3649 | 473750 | 1.04 (1.03-1.04) | 8.77E-49 | 3.43E-47 |
| 411.4 | Coronary atherosclerosis | circulatory system | 33501 | 475875 | 1.01 (1.01-1.01) | 1.09E-48 | 4.11E-47 |
| 562.1 | Diverticulosis | digestive | 59632 | 445200 | 1.01 (1.01-1.01) | 3.20E-48 | 1.16E-46 |
| 751.21 | Cystic kidney disease | congenital anomalies | 545 | 499974 | 1.08 (1.07-1.10) | 1.02E-47 | 3.59E-46 |
| 427.2 | Atrial fibrillation and flutter | circulatory system | 14749 | 464150 | 1.02 (1.02-1.02) | 4.51E-47 | 1.53E-45 |
| 416 | Cardiomegaly | circulatory system | 10470 | 493679 | 1.02 (1.02-1.02) | 5.62E-43 | 1.85E-41 |
| 250.22 | Type 2 diabetes with renal manifestations | endocrine/metabolic | 727 | 461081 | 1.07 (1.06-1.08) | 2.10E-42 | 6.71E-41 |
| 276.41 | Acidosis | endocrine/metabolic | 4667 | 474768 | 1.03 (1.03-1.03) | 3.48E-42 | 1.08E-40 |
| 250.13 | Type 1 diabetes with ophthalmic manifestations | endocrine/metabolic | 664 | 461018 | 0.92 (0.90-0.93) | 6.96E-39 | 2.10E-37 |
| 459.9 | Circulatory disease NEC | circulatory system | 36851 | 486149 | 1.01 (1.01-1.01) | 9.29E-39 | 2.72E-37 |
| 394.2 | Mitral valve disease | circulatory system | 10018 | 490513 | 1.02 (1.02-1.02) | 1.60E-38 | 4.56E-37 |
| 854 | Complications of cardiac/vascular device, implant, and graft | injuries & poisonings | 4738 | 477780 | 1.03 (1.02-1.03) | 7.15E-38 | 1.99E-36 |
| 250.1 | Type 1 diabetes | endocrine/metabolic | 2731 | 463085 | 0.96 (0.96-0.97) | 2.31E-37 | 6.25E-36 |
| 586 | Other disorders of the kidney and ureters | genitourinary | 2450 | 465262 | 1.04 (1.03-1.05) | 1.38E-36 | 3.64E-35 |
| 317.11 | Alcoholic liver damage | mental disorders | 1667 | 456685 | 1.04 (1.04-1.05) | 1.75E-36 | 4.50E-35 |
| 414 | Other forms of chronic heart disease | circulatory system | 9312 | 451686 | 1.02 (1.02-1.02) | 1.93E-35 | 4.85E-34 |
| 395.1 | Nonrheumatic mitral valve disorders | circulatory system | 9755 | 490250 | 1.02 (1.02-1.02) | 6.39E-35 | 1.57E-33 |
| 426.91 | Cardiac pacemaker in situ | circulatory system | 6906 | 456307 | 1.02 (1.02-1.03) | 1.85E-33 | 4.34E-32 |
| 580.32 | Nephritis and nephropathy with pathological lesion | genitourinary | 430 | 463242 | 1.08 (1.07-1.10) | 3.35E-32 | 7.68E-31 |
| 394.7 | Disease of tricuspid valve | circulatory system | 5825 | 486320 | 1.02 (1.02-1.03) | 9.96E-32 | 2.24E-30 |
| 276.5 | Hypovolemia | endocrine/metabolic | 11950 | 482051 | 1.02 (1.01-1.02) | 4.35E-29 | 9.56E-28 |
| 507 | Pleurisy; pleural effusion | respiratory | 18666 | 484265 | 1.01 (1.01-1.02) | 8.68E-29 | 1.87E-27 |
| 586.2 | Cyst of kidney, acquired | genitourinary | 5010 | 467822 | 1.02 (1.02-1.03) | 4.93E-27 | 1.04E-25 |
| 480 | Pneumonia | respiratory | 14745 | 483730 | 1.01 (1.01-1.02) | 9.12E-27 | 1.89E-25 |
| 317.1 | Alcoholism | mental disorders | 17047 | 472065 | 1.01 (1.01-1.02) | 1.07E-25 | 2.17E-24 |
| 411.3 | Angina pectoris | circulatory system | 20507 | 462881 | 1.01 (1.01-1.01) | 7.48E-25 | 1.49E-23 |
| 250.7 | Diabetic retinopathy | endocrine/metabolic | 3889 | 471444 | 0.97 (0.97-0.98) | 1.24E-24 | 2.42E-23 |
| 571.51 | Cirrhosis of liver without mention of alcohol | digestive | 2142 | 479570 | 1.03 (1.03-1.04) | 3.19E-24 | 6.12E-23 |
| 425.1 | Primary/intrinsic cardiomyopathies | circulatory system | 2592 | 496884 | 1.03 (1.02-1.04) | 8.11E-24 | 1.53E-22 |
| 427.42 | Cardiac arrest | circulatory system | 3042 | 452443 | 1.03 (1.02-1.03) | 4.73E-23 | 8.75E-22 |
| 227.2 | Benign neoplasm of parathyroid gland | neoplasms | 747 | 500470 | 1.05 (1.04-1.07) | 5.68E-23 | 1.03E-21 |
| 743.11 | Osteoporosis NOS | musculoskeletal | 18006 | 500210 | 0.99 (0.98-0.99) | 2.28E-22 | 4.08E-21 |
| 426.32 | Left bundle branch block | circulatory system | 5727 | 455128 | 1.02 (1.02-1.02) | 4.52E-22 | 7.95E-21 |
| 509.1 | Respiratory failure | respiratory | 8925 | 474524 | 1.02 (1.01-1.02) | 5.19E-22 | 8.98E-21 |
| 275.5 | Disorders of calcium/phosphorus metabolism | endocrine/metabolic | 4764 | 497674 | 1.02 (1.02-1.03) | 2.10E-21 | 3.57E-20 |
| 480.11 | Pneumococcal pneumonia | respiratory | 17616 | 486601 | 1.01 (1.01-1.01) | 3.15E-21 | 5.28E-20 |
| 38 | Septicemia | infectious diseases | 13870 | 484399 | 1.01 (1.01-1.02) | 3.42E-21 | 5.64E-20 |
| 557.1 | Celiac disease | digestive | 2303 | 387871 | 1.03 (1.03-1.04) | 1.06E-20 | 1.72E-19 |
| 272.1 | Hyperlipidemia | endocrine/metabolic | 11451 | 447721 | 1.01 (1.01-1.02) | 9.37E-20 | 1.50E-18 |
| 208 | Benign neoplasm of colon | neoplasms | 43801 | 497524 | 1.01 (1.01-1.01) | 1.63E-19 | 2.57E-18 |
| 415.21 | Primary pulmonary hypertension | circulatory system | 578 | 483787 | 1.05 (1.04-1.07) | 2.13E-19 | 3.30E-18 |
| 394 | Rheumatic disease of the heart valves | circulatory system | 4815 | 485310 | 1.02 (1.02-1.02) | 3.07E-19 | 4.63E-18 |
| 681.3 | Cellulitis and abscess of arm/hand | dermatologic | 9278 | 494703 | 1.01 (1.01-1.02) | 7.96E-19 | 1.15E-17 |
| 681.5 | Cellulitis and abscess of leg, except foot | dermatologic | 9278 | 494703 | 1.01 (1.01-1.02) | 7.96E-19 | 1.15E-17 |
| 681.6 | Cellulitis and abscess of foot, toe | dermatologic | 9278 | 494703 | 1.01 (1.01-1.02) | 7.96E-19 | 1.15E-17 |
| 509.2 | Respiratory insufficiency | respiratory | 7909 | 473508 | 1.02 (1.01-1.02) | 1.14E-18 | 1.63E-17 |
| 790.6 | Other abnormal blood chemistry | symptoms | 15488 | 501691 | 1.01 (1.01-1.01) | 1.27E-18 | 1.79E-17 |
| 994.2 | Sepsis | injuries & poisonings | 13467 | 502490 | 1.01 (1.01-1.01) | 1.29E-18 | 1.79E-17 |
| 345 | Epilepsy, recurrent seizures, convulsions | neurological | 4350 | 478044 | 0.98 (0.97-0.98) | 1.51E-18 | 2.07E-17 |
| 503 | Pulmonary congestion and hypostasis | respiratory | 1104 | 466703 | 1.04 (1.03-1.05) | 3.68E-18 | 4.98E-17 |
| 440.1 | Atherosclerosis of renal artery | circulatory system | 204 | 482095 | 1.08 (1.06-1.10) | 6.85E-18 | 9.15E-17 |
| 512.7 | Shortness of breath | respiratory | 13831 | 495988 | 1.01 (1.01-1.01) | 7.14E-18 | 9.42E-17 |
| 580.12 | Non-proliferative glomerulonephritis | genitourinary | 235 | 463047 | 1.08 (1.06-1.10) | 7.75E-18 | 1.01E-16 |
| 250.41 | Impaired fasting glucose | endocrine/metabolic | 2706 | 463060 | 1.03 (1.02-1.03) | 2.02E-17 | 2.60E-16 |
| 579.2 | Splenomegaly | digestive | 1133 | 469363 | 1.04 (1.03-1.05) | 3.13E-17 | 3.98E-16 |
| 697 | Sarcoidosis | dermatologic | 1069 | 490946 | 1.04 (1.03-1.05) | 4.99E-17 | 6.27E-16 |
| 332 | Parkinson's disease | neurological | 3836 | 477530 | 0.98 (0.97-0.98) | 2.17E-16 | 2.69E-15 |
| 345.12 | Partial epilepsy | neurological | 442 | 474136 | 0.93 (0.92-0.95) | 2.27E-16 | 2.78E-15 |
| 395.6 | Heart valve replaced | circulatory system | 3604 | 484099 | 1.02 (1.02-1.03) | 2.87E-16 | 3.48E-15 |
| 269 | Proteinuria | endocrine/metabolic | 473 | 500529 | 1.05 (1.04-1.07) | 4.60E-16 | 5.51E-15 |
| 571.81 | Portal hypertension | digestive | 1392 | 478820 | 1.03 (1.02-1.04) | 5.00E-16 | 5.93E-15 |
| 443.9 | Peripheral vascular disease, unspecified | circulatory system | 5593 | 487484 | 1.02 (1.01-1.02) | 3.94E-15 | 4.55E-14 |
| 508 | Pulmonary collapse; interstitial and compensatory emphysema | respiratory | 9497 | 475096 | 1.01 (1.01-1.02) | 3.97E-15 | 4.55E-14 |
| 250.23 | Type 2 diabetes with ophthalmic manifestations | endocrine/metabolic | 4590 | 464944 | 0.98 (0.98-0.99) | 5.10E-15 | 5.79E-14 |
| 600 | Hyperplasia of prostate | genitourinary | 22847 | 488487 | 0.99 (0.99-0.99) | 5.77E-15 | 6.48E-14 |
| 458.9 | Hypotension NOS | circulatory system | 13155 | 462453 | 1.01 (1.01-1.01) | 1.16E-14 | 1.29E-13 |
| 278.1 | Obesity | endocrine/metabolic | 37044 | 502394 | 1.01 (1.01-1.01) | 2.57E-14 | 2.82E-13 |
| 189 | Cancer of urinary organs (incl. kidney and bladder) | neoplasms | 4811 | 499526 | 1.02 (1.01-1.02) | 3.44E-14 | 3.74E-13 |
| 573.7 | Abnormal results of function study of liver | digestive | 8862 | 486290 | 1.01 (1.01-1.02) | 9.56E-14 | 1.03E-12 |
| 252.2 | Hypoparathyroidism | endocrine/metabolic | 513 | 493690 | 1.05 (1.04-1.06) | 1.41E-13 | 1.50E-12 |
| 250.11 | Type 1 diabetes with ketoacidosis | endocrine/metabolic | 320 | 460674 | 0.93 (0.91-0.95) | 3.23E-13 | 3.41E-12 |
| 519.8 | Other diseases of respiratory system, NEC | respiratory | 19858 | 501644 | 1.01 (1.01-1.01) | 3.89E-13 | 4.06E-12 |
| 433.21 | Cerebral artery occlusion, with cerebral infarction | circulatory system | 5849 | 482908 | 1.01 (1.01-1.02) | 1.64E-12 | 1.70E-11 |
| 275.3 | Disorders of magnesium metabolism | endocrine/metabolic | 3573 | 496483 | 1.02 (1.01-1.02) | 1.99E-12 | 2.04E-11 |
| 244.4 | Hypothyroidism NOS | endocrine/metabolic | 23461 | 495955 | 1.01 (1.01-1.01) | 3.62E-12 | 3.67E-11 |
| 964.1 | Anticoagulants causing adverse effects | injuries & poisonings | 760 | 453432 | 1.04 (1.03-1.05) | 1.00E-11 | 1.00E-10 |
| 433.2 | Occlusion of cerebral arteries | circulatory system | 7579 | 484638 | 1.01 (1.01-1.02) | 1.02E-11 | 1.02E-10 |
| 427.12 | Paroxysmal ventricular tachycardia | circulatory system | 1938 | 451339 | 1.02 (1.02-1.03) | 1.27E-11 | 1.25E-10 |
| 586.3 | Vascular disorders of kidney/hypertrophy | genitourinary | 277 | 463089 | 1.06 (1.04-1.08) | 1.58E-11 | 1.54E-10 |
| 726.3 | Bursitis | musculoskeletal | 962 | 460460 | 1.03 (1.02-1.04) | 2.50E-11 | 2.42E-10 |
| 433.1 | Occlusion and stenosis of precerebral arteries | circulatory system | 2578 | 479637 | 1.02 (1.01-1.03) | 3.64E-11 | 3.49E-10 |
| 394.3 | Aortic valve disease | circulatory system | 4175 | 484670 | 1.02 (1.01-1.02) | 5.51E-11 | 5.24E-10 |
| 427.3 | Other specified cardiac dysrhythmias | circulatory system | 8699 | 458100 | 1.01 (1.01-1.01) | 8.39E-11 | 7.90E-10 |
| 41.4 | E. coli | infectious diseases | 7865 | 478394 | 1.01 (1.01-1.02) | 1.01E-10 | 9.35E-10 |
| 573.3 | Hepatomegaly | digestive | 620 | 478048 | 1.04 (1.03-1.05) | 1.36E-10 | 1.24E-09 |
| 420.2 | Pericarditis | circulatory system | 2874 | 497166 | 1.02 (1.01-1.02) | 1.64E-10 | 1.48E-09 |
| 174.11 | Malignant neoplasm of female breast | neoplasms | 17063 | 494930 | 1.01 (1.01-1.01) | 1.79E-10 | 1.60E-09 |
| 70.3 | Viral hepatitis C | infectious diseases | 269 | 494431 | 1.05 (1.04-1.07) | 3.43E-10 | 3.04E-09 |
| 589 | Abnormal results of function study of kidney | genitourinary | 431 | 463243 | 1.05 (1.03-1.06) | 3.48E-10 | 3.06E-09 |
| 741.4 | Joint effusions | musculoskeletal | 2751 | 495906 | 1.02 (1.01-1.02) | 4.13E-10 | 3.60E-09 |
| 275.1 | Disorders of iron metabolism | hematopoietic | 1124 | 494034 | 1.03 (1.02-1.04) | 4.68E-10 | 4.05E-09 |
| 41 | Bacterial infection NOS | infectious diseases | 8927 | 479456 | 1.01 (1.01-1.01) | 5.37E-10 | 4.61E-09 |
| 590 | Pyelonephritis | genitourinary | 2861 | 465922 | 1.02 (1.01-1.02) | 7.25E-10 | 6.17E-09 |
| 442.11 | Abdominal aortic aneurysm | circulatory system | 2619 | 484510 | 1.02 (1.01-1.02) | 2.07E-09 | 1.75E-08 |
| 496.21 | Obstructive chronic bronchitis | respiratory | 8106 | 455019 | 1.01 (1.01-1.01) | 2.10E-09 | 1.76E-08 |
| 571.8 | Liver abscess and sequelae of chronic liver disease | digestive | 1710 | 479138 | 1.02 (1.01-1.03) | 2.27E-09 | 1.89E-08 |
| 287.3 | Thrombocytopenia | hematopoietic | 3777 | 500158 | 1.01 (1.01-1.02) | 2.54E-09 | 2.09E-08 |
| 567 | Peritonitis and retroperitoneal infections | digestive | 1747 | 468474 | 1.02 (1.01-1.03) | 2.74E-09 | 2.24E-08 |
| 440.9 | Atherosclerosis of aorta | circulatory system | 1477 | 483368 | 1.02 (1.02-1.03) | 2.85E-09 | 2.31E-08 |
| 572 | Ascites (non malignant) | digestive | 3898 | 481326 | 1.01 (1.01-1.02) | 3.05E-09 | 2.46E-08 |
| 711.1 | Pyogenic arthritis | musculoskeletal | 584 | 414708 | 1.04 (1.02-1.05) | 3.27E-09 | 2.61E-08 |
| 480.2 | Viral pneumonia | respiratory | 3526 | 472511 | 1.02 (1.01-1.02) | 3.77E-09 | 2.99E-08 |
| 411.9 | Other acute and subacute forms of ischemic heart disease | circulatory system | 3740 | 446114 | 1.01 (1.01-1.02) | 4.87E-09 | 3.83E-08 |
| 447 | Other disorders of arteries and arterioles | circulatory system | 1097 | 482988 | 1.03 (1.02-1.04) | 1.04E-08 | 8.13E-08 |
| 578.2 | Blood in stool | digestive | 3950 | 472180 | 1.01 (1.01-1.02) | 1.05E-08 | 8.15E-08 |
| 401.21 | Hypertensive heart disease | circulatory system | 945 | 385157 | 1.03 (1.02-1.04) | 1.17E-08 | 9.01E-08 |
| 418 | Nonspecific chest pain | circulatory system | 30254 | 496797 | 1.01 (1.00-1.01) | 1.23E-08 | 9.40E-08 |
| 974 | Poisoning by water, mineral, and uric acid metabolism drugs | injuries & poisonings | 331 | 453003 | 1.04 (1.03-1.06) | 1.80E-08 | 1.37E-07 |
| 535.6 | Duodenitis | digestive | 9152 | 465248 | 1.01 (1.01-1.01) | 1.83E-08 | 1.38E-07 |
| 250.21 | Type 2 diabetes with ketoacidosis | endocrine/metabolic | 483 | 460837 | 0.96 (0.95-0.97) | 2.31E-08 | 1.73E-07 |
| 477 | Epistaxis or throat hemorrhage | respiratory | 3770 | 484661 | 1.01 (1.01-1.02) | 2.40E-08 | 1.78E-07 |
| 276.14 | Hypopotassemia | endocrine/metabolic | 8231 | 478332 | 1.01 (1.01-1.01) | 2.59E-08 | 1.91E-07 |
| 426.24 | Atrioventricular block, complete | circulatory system | 2015 | 451416 | 1.02 (1.01-1.03) | 3.77E-08 | 2.76E-07 |
| 747.13 | Congenital anomalies of great vessels | congenital anomalies | 5487 | 501024 | 1.01 (1.01-1.01) | 5.13E-08 | 3.73E-07 |
| 596 | Other disorders of bladder | genitourinary | 11648 | 493331 | 0.99 (0.99-0.99) | 5.43E-08 | 3.92E-07 |
| 272.13 | Mixed hyperlipidemia | endocrine/metabolic | 384 | 436654 | 1.04 (1.03-1.06) | 6.20E-08 | 4.45E-07 |
| 411.1 | Unstable angina (intermediate coronary syndrome) | circulatory system | 4325 | 446699 | 1.01 (1.01-1.02) | 7.31E-08 | 5.21E-07 |
| 1019 | Other ill-defined and unknown causes of morbidity and mortality | NULL | 14981 | 502490 | 1.01 (1.00-1.01) | 7.39E-08 | 5.23E-07 |
| 290.12 | Dementia with cerebral degenerations | mental disorders | 520 | 474358 | 0.96 (0.95-0.98) | 7.61E-08 | 5.35E-07 |
| 426.31 | Right bundle branch block | circulatory system | 5817 | 455218 | 1.01 (1.01-1.01) | 8.19E-08 | 5.72E-07 |
| 281.13 | Folate-deficiency anemia | hematopoietic | 823 | 460693 | 1.03 (1.02-1.04) | 9.91E-08 | 6.88E-07 |
| 290.1 | Dementias | mental disorders | 6861 | 480699 | 0.99 (0.99-0.99) | 1.00E-07 | 6.90E-07 |
| 962.3 | Hormones and synthetic substitutes causing adverse effects in therapeutic use | injuries & poisonings | 770 | 453442 | 1.03 (1.02-1.04) | 1.20E-07 | 8.22E-07 |
| 550.1 | Inguinal hernia | digestive | 16285 | 458146 | 0.99 (0.99-1.00) | 1.33E-07 | 9.05E-07 |
| 707 | Chronic ulcer of skin | dermatologic | 3780 | 502297 | 1.01 (1.01-1.02) | 1.52E-07 | 1.03E-06 |
| 41.1 | Staphylococcus infections | infectious diseases | 5120 | 475649 | 1.01 (1.01-1.02) | 1.78E-07 | 1.20E-06 |
| 70.9 | Hepatitis NOS | infectious diseases | 1089 | 495251 | 1.02 (1.01-1.03) | 2.11E-07 | 1.41E-06 |
| 558 | Noninfectious gastroenteritis | digestive | 12569 | 398137 | 1.01 (1.00-1.01) | 2.38E-07 | 1.58E-06 |
| 415 | Pulmonary heart disease | circulatory system | 8631 | 491840 | 1.01 (1.01-1.01) | 2.60E-07 | 1.71E-06 |
| 189.11 | Malignant neoplasm of kidney, except pelvis | neoplasms | 2219 | 496934 | 1.02 (1.01-1.02) | 3.23E-07 | 2.12E-06 |
| 502 | Postinflammatory pulmonary fibrosis | respiratory | 3460 | 469059 | 1.01 (1.01-1.02) | 3.58E-07 | 2.33E-06 |
| 345.11 | Generalized convulsive epilepsy | neurological | 708 | 474402 | 0.97 (0.96-0.98) | 5.16E-07 | 3.34E-06 |
| 425.12 | Other hypertrophic cardiomyopathy | circulatory system | 475 | 494767 | 1.03 (1.02-1.05) | 5.40E-07 | 3.47E-06 |
| 300.1 | Anxiety disorder | mental disorders | 22372 | 428860 | 0.99 (0.99-1.00) | 5.48E-07 | 3.50E-06 |
| 596.5 | Functional disorders of bladder | genitourinary | 1231 | 482914 | 0.98 (0.97-0.99) | 5.90E-07 | 3.75E-06 |
| 496.1 | Emphysema | respiratory | 5151 | 452064 | 1.01 (1.01-1.02) | 6.15E-07 | 3.89E-06 |
| 717 | Polymyalgia Rheumatica | musculoskeletal | 4194 | 502490 | 1.01 (1.01-1.02) | 7.63E-07 | 4.79E-06 |
| 426.21 | First degree AV block | circulatory system | 4544 | 453945 | 1.01 (1.01-1.02) | 7.99E-07 | 4.99E-06 |
| 38.1 | Gram negative septicemia | infectious diseases | 3361 | 473890 | 1.01 (1.01-1.02) | 8.75E-07 | 5.43E-06 |
| 751.22 | Other specified congenital anomalies of kidney | congenital anomalies | 645 | 500074 | 1.03 (1.02-1.04) | 8.83E-07 | 5.45E-06 |
| 563 | Constipation | digestive | 28115 | 502490 | 1.00 (0.99-1.00) | 1.21E-06 | 7.42E-06 |
| 696.4 | Psoriasis | dermatologic | 3766 | 485143 | 1.01 (1.01-1.02) | 2.20E-06 | 1.34E-05 |
| 200 | Myeloproliferative disease | neoplasms | 1919 | 494635 | 1.02 (1.01-1.02) | 2.27E-06 | 1.37E-05 |
| 285 | Other anemias | hematopoietic | 24809 | 484679 | 1.00 (1.00-1.01) | 2.33E-06 | 1.40E-05 |
| 681 | Superficial cellulitis and abscess | dermatologic | 766 | 486191 | 1.03 (1.01-1.04) | 2.66E-06 | 1.59E-05 |
| 335 | Multiple sclerosis | neurological | 1075 | 474769 | 0.97 (0.96-0.99) | 2.78E-06 | 1.65E-05 |
| 420.3 | Endocarditis | circulatory system | 1915 | 496207 | 1.02 (1.01-1.02) | 2.95E-06 | 1.74E-05 |
| 340 | Migraine | neurological | 6204 | 491970 | 0.99 (0.99-0.99) | 3.24E-06 | 1.90E-05 |
| 737.3 | Kyphoscoliosis and scoliosis | musculoskeletal | 3352 | 485605 | 0.99 (0.98-0.99) | 3.29E-06 | 1.92E-05 |
| 211 | Benign neoplasm of other parts of digestive system | neoplasms | 12738 | 484773 | 0.99 (0.99-1.00) | 3.52E-06 | 2.04E-05 |
| 442.1 | Aortic aneurysm | circulatory system | 2258 | 484149 | 1.01 (1.01-1.02) | 3.69E-06 | 2.13E-05 |
| 599.4 | Urinary incontinence | genitourinary | 11222 | 474997 | 0.99 (0.99-1.00) | 3.71E-06 | 2.13E-05 |
| 175 | Acquired absence of breast | neoplasms | 4789 | 482106 | 1.01 (1.01-1.02) | 3.76E-06 | 2.14E-05 |
| 946 | Anaphylactic shock NOS | injuries & poisonings | 715 | 493155 | 1.03 (1.01-1.04) | 4.06E-06 | 2.30E-05 |
| 803.2 | Fracture of radius and ulna | injuries & poisonings | 6549 | 478368 | 0.99 (0.99-0.99) | 4.26E-06 | 2.40E-05 |
| 425.11 | Hypertrophic obstructive cardiomyopathy | circulatory system | 306 | 494598 | 1.04 (1.02-1.06) | 4.76E-06 | 2.67E-05 |
| 696.41 | Psoriasis vulgaris | dermatologic | 4056 | 485433 | 1.01 (1.01-1.02) | 4.92E-06 | 2.75E-05 |
| 427.41 | Ventricular fibrillation and flutter | circulatory system | 850 | 450251 | 1.02 (1.01-1.03) | 5.53E-06 | 3.07E-05 |
| 782.3 | Edema | symptoms | 7066 | 502069 | 1.01 (1.00-1.01) | 6.54E-06 | 3.61E-05 |
| 433.8 | Late effects of cerebrovascular disease | circulatory system | 3350 | 480409 | 1.01 (1.01-1.02) | 7.27E-06 | 3.99E-05 |
| 274.21 | Chondrocalcinosis | endocrine/metabolic | 885 | 492485 | 1.02 (1.01-1.03) | 7.73E-06 | 4.23E-05 |
| 960.2 | Allergy/adverse effect of penicillin | injuries & poisonings | 26661 | 479333 | 1.00 (1.00-1.01) | 7.83E-06 | 4.26E-05 |
| 280.1 | Iron deficiency anemias, unspecified or not due to blood loss | hematopoietic | 20386 | 480256 | 0.99 (0.99-1.00) | 8.67E-06 | 4.69E-05 |
| 182 | Malignant neoplasm of uterus | neoplasms | 2077 | 476382 | 1.02 (1.01-1.02) | 9.29E-06 | 5.00E-05 |
| 285.1 | Acute posthemorrhagic anemia | hematopoietic | 226 | 460096 | 1.04 (1.02-1.06) | 1.50E-05 | 8.03E-05 |
| 359.2 | Myopathy | neurological | 626 | 497706 | 1.03 (1.01-1.04) | 1.51E-05 | 8.05E-05 |
| 496 | Chronic airway obstruction | respiratory | 19413 | 466326 | 1.01 (1.00-1.01) | 1.71E-05 | 9.07E-05 |
| 771.1 | Swelling of limb | symptoms | 9225 | 501723 | 1.01 (1.00-1.01) | 1.84E-05 | 9.71E-05 |
| 516.1 | Hemoptysis | respiratory | 2729 | 502305 | 1.01 (1.01-1.02) | 1.90E-05 | 9.97E-05 |
| 367.1 | Myopia | sense organs | 3690 | 492117 | 1.01 (1.01-1.02) | 2.01E-05 | 1.05E-04 |
| 790 | Nonspecific findings on examination of blood | symptoms | 901 | 487104 | 1.02 (1.01-1.03) | 2.11E-05 | 1.10E-04 |
| 480.1 | Bacterial pneumonia | respiratory | 2649 | 471634 | 1.01 (1.01-1.02) | 2.33E-05 | 1.20E-04 |
| 200.1 | Polycythemia vera | neoplasms | 430 | 486051 | 1.03 (1.02-1.05) | 2.44E-05 | 1.26E-04 |
| 451.2 | Phlebitis and thrombophlebitis of lower extremities | circulatory system | 5943 | 473747 | 1.01 (1.00-1.01) | 2.62E-05 | 1.34E-04 |
| 276.1 | Electrolyte imbalance | endocrine/metabolic | 780 | 470881 | 1.02 (1.01-1.03) | 2.70E-05 | 1.38E-04 |
| 271.3 | Intestinal disaccharidase deficiencies and disaccharide malabsorption | endocrine/metabolic | 443 | 502452 | 1.03 (1.02-1.05) | 2.79E-05 | 1.42E-04 |
| 318 | Tobacco use disorder | mental disorders | 27653 | 482671 | 1.00 (1.00-1.01) | 2.94E-05 | 1.48E-04 |
| 599.6 | Oliguria and anuria | genitourinary | 360 | 464135 | 1.03 (1.02-1.05) | 2.99E-05 | 1.50E-04 |
| 452 | Other venous embolism and thrombosis | circulatory system | 1369 | 469173 | 1.02 (1.01-1.03) | 3.35E-05 | 1.68E-04 |
| 395.2 | Nonrheumatic aortic valve disorders | circulatory system | 1068 | 481563 | 1.02 (1.01-1.03) | 3.72E-05 | 1.85E-04 |
| 8.52 | Intestinal infection due to C. difficile | infectious diseases | 1359 | 498421 | 1.02 (1.01-1.03) | 3.79E-05 | 1.87E-04 |
| 857 | Mechanical complication of unspecified genitourinary device, implant, and graft | injuries & poisonings | 3485 | 476527 | 0.99 (0.98-0.99) | 3.80E-05 | 1.87E-04 |
| 292.2 | Mild cognitive impairment | mental disorders | 1239 | 475077 | 0.98 (0.97-0.99) | 3.96E-05 | 1.94E-04 |
| 1005 | Other symptoms | NULL | 4432 | 502490 | 1.01 (1.00-1.01) | 4.86E-05 | 2.37E-04 |
| 729 | Other disorders of soft tissues | musculoskeletal | 9793 | 469291 | 1.01 (1.00-1.01) | 5.12E-05 | 2.48E-04 |
| 504 | Other alveolar and parietoalveolar pneumonopathy | respiratory | 1466 | 467065 | 1.02 (1.01-1.02) | 5.12E-05 | 2.48E-04 |
| 447.1 | Stricture of artery | circulatory system | 1526 | 483417 | 1.02 (1.01-1.02) | 5.22E-05 | 2.51E-04 |
| 733 | Other disorders of bone and cartilage | musculoskeletal | 6909 | 480882 | 0.99 (0.99-1.00) | 5.45E-05 | 2.61E-04 |
| 599 | Other symptoms/disorders or the urinary system | genitourinary | 11507 | 475282 | 0.99 (0.99-1.00) | 6.31E-05 | 3.01E-04 |
| 695.9 | Unspecified erythematous condition | dermatologic | 1204 | 491081 | 1.02 (1.01-1.03) | 6.85E-05 | 3.26E-04 |
| 818 | Intracranial hemorrhage (injury) | injuries & poisonings | 2114 | 499447 | 1.01 (1.01-1.02) | 6.95E-05 | 3.29E-04 |
| 444.1 | Arterial embolism and thrombosis of lower extremity artery | circulatory system | 969 | 482860 | 1.02 (1.01-1.03) | 7.04E-05 | 3.32E-04 |
| 947 | Urticaria | dermatologic | 1005 | 493445 | 1.02 (1.01-1.03) | 7.37E-05 | 3.46E-04 |
| 440.2 | Atherosclerosis of the extremities | circulatory system | 1766 | 483657 | 1.01 (1.01-1.02) | 7.98E-05 | 3.73E-04 |
| 426.23 | Second degree AV block | circulatory system | 1518 | 450919 | 1.02 (1.01-1.02) | 8.04E-05 | 3.74E-04 |
| 242 | Thyrotoxicosis with or without goiter | endocrine/metabolic | 2726 | 475220 | 1.01 (1.01-1.02) | 8.59E-05 | 3.97E-04 |
| 745 | Pain in joint | musculoskeletal | 12822 | 502490 | 1.01 (1.00-1.01) | 8.90E-05 | 4.10E-04 |
| 509.8 | Dependence on respirator [Ventilator] or supplemental oxygen | respiratory | 1703 | 467302 | 1.01 (1.01-1.02) | 9.47E-05 | 4.34E-04 |
| 510 | Other diseases of lung | respiratory | 4079 | 502445 | 1.01 (1.00-1.01) | 9.88E-05 | 4.51E-04 |
| 496.3 | Bronchiectasis | respiratory | 5810 | 452723 | 1.01 (1.00-1.01) | 1.05E-04 | 4.76E-04 |
| 79 | Viral infection | infectious diseases | 3893 | 498055 | 1.01 (1.00-1.02) | 1.09E-04 | 4.92E-04 |
| 395.3 | Nonrheumatic tricuspid valve disorders | circulatory system | 680 | 481175 | 1.02 (1.01-1.03) | 1.10E-04 | 4.95E-04 |
| 426 | Cardiac conduction disorders | circulatory system | 423 | 449824 | 1.03 (1.01-1.04) | 1.15E-04 | 5.17E-04 |
| 342 | Hemiplegia | neurological | 3920 | 477614 | 1.01 (1.00-1.01) | 1.18E-04 | 5.29E-04 |
| 530.3 | Stricture and stenosis of esophagus | digestive | 3659 | 436030 | 1.01 (1.00-1.02) | 1.22E-04 | 5.44E-04 |
| 577 | Diseases of pancreas | digestive | 1935 | 500806 | 1.01 (1.01-1.02) | 1.24E-04 | 5.51E-04 |
| 573.5 | Jaundice (not of newborn) | digestive | 1446 | 478874 | 1.02 (1.01-1.02) | 1.27E-04 | 5.59E-04 |
| 531.3 | Duodenal ulcer | digestive | 3301 | 495747 | 1.01 (1.00-1.02) | 1.35E-04 | 5.95E-04 |
| 716.2 | Unspecified monoarthritis | musculoskeletal | 29738 | 443862 | 1.00 (1.00-1.01) | 1.38E-04 | 6.03E-04 |
| 418.1 | Precordial pain | circulatory system | 8011 | 474554 | 1.01 (1.00-1.01) | 1.74E-04 | 7.55E-04 |
| 532 | Dysphagia | digestive | 14153 | 446524 | 0.99 (0.99-1.00) | 1.80E-04 | 7.78E-04 |
| 481 | Influenza | respiratory | 1821 | 470806 | 1.01 (1.01-1.02) | 1.94E-04 | 8.35E-04 |
| 495 | Asthma | respiratory | 33045 | 479958 | 1.00 (1.00-1.01) | 1.95E-04 | 8.36E-04 |
| 598 | Abnormal findings on examination of urine | genitourinary | 3891 | 502490 | 0.99 (0.99-1.00) | 2.11E-04 | 9.00E-04 |
| 531.1 | Hemorrhage from gastrointestinal ulcer | digestive | 966 | 493412 | 1.02 (1.01-1.03) | 2.31E-04 | 9.84E-04 |
| 41.2 | Streptococcus infection | infectious diseases | 3478 | 474007 | 1.01 (1.00-1.01) | 2.35E-04 | 9.98E-04 |
| 626 | Disorders of menstruation and other abnormal bleeding from female genital tract | genitourinary | 837 | 489289 | 1.02 (1.01-1.03) | 2.37E-04 | 9.99E-04 |
| 290.11 | Alzheimer's disease | mental disorders | 4267 | 478105 | 0.99 (0.99-1.00) | 2.38E-04 | 9.99E-04 |
| 939 | Atopic/contact dermatitis due to other or unspecified | dermatologic | 6918 | 499358 | 1.01 (1.00-1.01) | 2.52E-04 | 1.05E-03 |
| 565.1 | Anal and rectal polyp | digestive | 12618 | 479345 | 1.01 (1.00-1.01) | 2.72E-04 | 1.13E-03 |
| 286.7 | Other and unspecified coagulation defects | hematopoietic | 567 | 496948 | 1.02 (1.01-1.04) | 2.84E-04 | 1.18E-03 |
| 289.4 | Lymphadenitis | hematopoietic | 5057 | 491915 | 1.01 (1.00-1.01) | 2.88E-04 | 1.18E-03 |
| 578.9 | Hemorrhage of gastrointestinal tract | digestive | 15271 | 483501 | 1.00 (1.00-1.01) | 2.92E-04 | 1.20E-03 |
| 433 | Cerebrovascular disease | circulatory system | 5046 | 482105 | 1.01 (1.00-1.01) | 3.05E-04 | 1.25E-03 |
| 427.9 | Palpitations | circulatory system | 6236 | 455637 | 1.01 (1.00-1.01) | 4.04E-04 | 1.64E-03 |
| 270.32 | Paraproteinemia | endocrine/metabolic | 1259 | 501315 | 1.02 (1.01-1.02) | 4.09E-04 | 1.65E-03 |
| 741.5 | Hemarthrosis | musculoskeletal | 223 | 493378 | 1.03 (1.01-1.05) | 5.44E-04 | 2.19E-03 |
| 577.2 | Chronic pancreatitis | digestive | 841 | 499712 | 1.02 (1.01-1.03) | 6.06E-04 | 2.42E-03 |
| 586.4 | Stricture/obstruction of ureter | genitourinary | 1761 | 464573 | 1.01 (1.01-1.02) | 6.06E-04 | 2.42E-03 |
| 574.3 | Cholecystitis without cholelithiasis | digestive | 3429 | 482074 | 1.01 (1.00-1.01) | 6.23E-04 | 2.48E-03 |
| 785 | Abdominal pain | symptoms | 43507 | 502490 | 1.00 (1.00-1.00) | 6.33E-04 | 2.51E-03 |
| 870.3 | Other open wound of head and face | injuries & poisonings | 6023 | 495453 | 1.01 (1.00-1.01) | 6.46E-04 | 2.55E-03 |
| 574.11 | Cholelithiasis with acute cholecystitis | digestive | 2747 | 481392 | 1.01 (1.00-1.02) | 6.57E-04 | 2.59E-03 |
| 550.4 | Umbilical hernia | digestive | 4985 | 446846 | 1.01 (1.00-1.01) | 6.66E-04 | 2.61E-03 |
| 433.31 | Transient cerebral ischemia | circulatory system | 3604 | 480663 | 1.01 (1.00-1.01) | 6.71E-04 | 2.62E-03 |
| 480.12 | Pseudomonal pneumonia | respiratory | 262 | 469247 | 1.03 (1.01-1.05) | 6.73E-04 | 2.62E-03 |
| 1009 | Injury, NOS | NULL | 10103 | 502490 | 1.01 (1.00-1.01) | 6.91E-04 | 2.68E-03 |
| 531.2 | Gastric ulcer | digestive | 6181 | 498627 | 1.01 (1.00-1.01) | 7.04E-04 | 2.72E-03 |
| 345.1 | Epilepsy | neurological | 704 | 474398 | 0.98 (0.97-0.99) | 7.13E-04 | 2.75E-03 |
| 459 | Other disorders of circulatory system | circulatory system | 306 | 449604 | 1.03 (1.01-1.05) | 7.16E-04 | 2.75E-03 |
| 444 | Arterial embolism and thrombosis | circulatory system | 772 | 482663 | 1.02 (1.01-1.03) | 7.70E-04 | 2.94E-03 |
| 594.2 | Calculus of lower urinary tract | genitourinary | 1463 | 492291 | 0.99 (0.98-0.99) | 8.31E-04 | 3.17E-03 |
| 621 | Endometrial hyperplasia | genitourinary | 766 | 495805 | 1.02 (1.01-1.03) | 8.62E-04 | 3.26E-03 |
| 250.14 | Type 1 diabetes with neurological manifestations | endocrine/metabolic | 260 | 460614 | 0.97 (0.95-0.99) | 8.63E-04 | 3.26E-03 |
| 202 | Cancer of other lymphoid, histiocytic tissue | neoplasms | 2533 | 495249 | 1.01 (1.00-1.02) | 8.74E-04 | 3.29E-03 |
| 289 | Other diseases of blood and blood-forming organs | hematopoietic | 832 | 487690 | 1.02 (1.01-1.03) | 8.97E-04 | 3.37E-03 |
| 560.1 | Paralytic ileus | digestive | 1658 | 387226 | 1.01 (1.01-1.02) | 9.12E-04 | 3.41E-03 |
| 960 | Poisoning by antibiotics | injuries & poisonings | 10220 | 462892 | 1.01 (1.00-1.01) | 9.18E-04 | 3.42E-03 |
| 480.5 | Bronchopneumonia and lung abscess | respiratory | 2319 | 471304 | 1.01 (1.00-1.02) | 9.31E-04 | 3.46E-03 |
| 198.2 | Secondary malignancy of respiratory organs | neoplasms | 6988 | 441349 | 1.01 (1.00-1.01) | 9.53E-04 | 3.53E-03 |
| 381.1 | Otitis media | sense organs | 848 | 499936 | 0.98 (0.97-0.99) | 9.79E-04 | 3.61E-03 |
| 687.1 | Rash and other nonspecific skin eruption | dermatologic | 4388 | 497339 | 1.01 (1.00-1.01) | 9.83E-04 | 3.61E-03 |
| 316 | Substance addiction and disorders | mental disorders | 649 | 455667 | 0.98 (0.97-0.99) | 9.91E-04 | 3.63E-03 |
| 564.9 | Personal history of diseases of digestive system | digestive | 33640 | 419208 | 1.00 (1.00-1.00) | 9.95E-04 | 3.63E-03 |
| 441.1 | Acute vascular insufficiency of intestine | circulatory system | 792 | 482683 | 1.02 (1.01-1.03) | 9.98E-04 | 3.63E-03 |
| 573 | Other disorders of liver | digestive | 5191 | 482619 | 1.01 (1.00-1.01) | 1.14E-03 | 4.12E-03 |
| 427.7 | Tachycardia NOS | circulatory system | 6392 | 455793 | 1.01 (1.00-1.01) | 1.16E-03 | 4.18E-03 |
| 791 | Gangrene | symptoms | 1735 | 502490 | 1.01 (1.00-1.02) | 1.17E-03 | 4.18E-03 |
| 289.8 | Polycythemia, secondary | hematopoietic | 719 | 486200 | 1.02 (1.01-1.03) | 1.17E-03 | 4.18E-03 |
| 626.14 | Irregular menstrual bleeding | genitourinary | 950 | 489402 | 0.98 (0.97-0.99) | 1.21E-03 | 4.34E-03 |
| 157 | Pancreatic cancer | neoplasms | 2138 | 471586 | 1.01 (1.00-1.02) | 1.23E-03 | 4.38E-03 |
| 284 | Aplastic anemia | hematopoietic | 1344 | 461214 | 1.01 (1.01-1.02) | 1.27E-03 | 4.50E-03 |
| 261.4 | Vitamin D deficiency | endocrine/metabolic | 6403 | 494978 | 1.01 (1.00-1.01) | 1.30E-03 | 4.59E-03 |
| 427.8 | Sinoatrial node dysfunction (Bradycardia) | circulatory system | 1322 | 450723 | 1.01 (1.01-1.02) | 1.37E-03 | 4.85E-03 |
| 440 | Atherosclerosis | circulatory system | 952 | 482843 | 1.02 (1.01-1.03) | 1.38E-03 | 4.85E-03 |
| 574.1 | Cholelithiasis | digestive | 11157 | 489802 | 1.00 (1.00-1.01) | 1.47E-03 | 5.15E-03 |
| 427.11 | Paroxysmal supraventricular tachycardia | circulatory system | 3752 | 453153 | 1.01 (1.00-1.01) | 1.53E-03 | 5.36E-03 |
| 559 | Ileostomy status | digestive | 2414 | 387982 | 1.01 (1.00-1.02) | 1.67E-03 | 5.82E-03 |
| 706.2 | Sebaceous cyst | dermatologic | 5629 | 501073 | 0.99 (0.99-1.00) | 1.80E-03 | 6.24E-03 |
| 747.11 | Cardiac shunt/ heart septal defect | congenital anomalies | 772 | 496309 | 1.02 (1.01-1.03) | 1.86E-03 | 6.43E-03 |
| 426.3 | Bundle branch block | circulatory system | 1124 | 450525 | 1.01 (1.01-1.02) | 1.97E-03 | 6.78E-03 |
| 253.7 | Other disorders of neurohypophysis | endocrine/metabolic | 697 | 493874 | 0.98 (0.97-0.99) | 2.01E-03 | 6.90E-03 |
| 575.1 | Cholangitis | digestive | 1709 | 480354 | 1.01 (1.00-1.02) | 2.01E-03 | 6.90E-03 |
| 287 | Purpura and other hemorrhagic conditions | hematopoietic | 362 | 496743 | 1.02 (1.01-1.04) | 2.18E-03 | 7.44E-03 |
| 596.1 | Bladder neck obstruction | genitourinary | 1783 | 483466 | 0.99 (0.98-1.00) | 2.19E-03 | 7.46E-03 |
| 574.2 | Calculus of bile duct | digestive | 3754 | 482399 | 1.01 (1.00-1.01) | 2.22E-03 | 7.52E-03 |
| 800.1 | Fracture of neck of femur | injuries & poisonings | 5252 | 477071 | 0.99 (0.99-1.00) | 2.23E-03 | 7.54E-03 |
| 783 | Fever of unknown origin | symptoms | 7868 | 502490 | 1.01 (1.00-1.01) | 2.24E-03 | 7.55E-03 |
| 272.9 | Unspecified disorder of lipoid metabolism | endocrine/metabolic | 1008 | 437278 | 1.01 (1.01-1.02) | 2.28E-03 | 7.67E-03 |
| 274.2 | Crystal arthropathies | endocrine/metabolic | 308 | 491908 | 1.03 (1.01-1.04) | 2.31E-03 | 7.73E-03 |
| 262 | Mineral deficiency NEC | endocrine/metabolic | 1322 | 489897 | 1.01 (1.00-1.02) | 2.31E-03 | 7.73E-03 |
| 70.2 | Viral hepatitis B | infectious diseases | 317 | 494479 | 1.03 (1.01-1.04) | 2.35E-03 | 7.81E-03 |
| 351 | Other peripheral nerve disorders | neurological | 12229 | 497732 | 1.00 (0.99-1.00) | 2.43E-03 | 8.05E-03 |
| 618.1 | Prolapse of vaginal walls | genitourinary | 6982 | 498571 | 0.99 (0.99-1.00) | 2.47E-03 | 8.17E-03 |
| 599.9 | Other abnormality of urination | genitourinary | 1500 | 465275 | 0.99 (0.98-1.00) | 2.51E-03 | 8.27E-03 |
| 395.4 | Nonrheumatic pulmonary valve disorders | circulatory system | 908 | 481403 | 1.02 (1.01-1.03) | 2.53E-03 | 8.31E-03 |
| 738.4 | Acquired spondylolisthesis | musculoskeletal | 3625 | 485878 | 0.99 (0.99-1.00) | 2.54E-03 | 8.31E-03 |
| 338.2 | Chronic pain | neurological | 918 | 501937 | 0.98 (0.97-0.99) | 2.57E-03 | 8.39E-03 |
| 698 | Pruritus and related conditions | dermatologic | 1241 | 502490 | 1.01 (1-1.02) | 2.60E-03 | 8.46E-03 |
| 255.21 | Glucocorticoid deficiency | endocrine/metabolic | 884 | 494061 | 1.02 (1.01-1.03) | 2.61E-03 | 8.48E-03 |
| 773 | Pain in limb | symptoms | 9893 | 502490 | 1 (1-1.01) | 2.62E-03 | 8.49E-03 |
| 721.1 | Spondylosis without myelopathy | musculoskeletal | 12860 | 483562 | 1 (0.99-1) | 2.92E-03 | 9.43E-03 |
| 557 | Intestinal malabsorption (non-celiac) | digestive | 599 | 386167 | 1.02 (1.01-1.03) | 3.06E-03 | 9.83E-03 |
| 512.8 | Cough | respiratory | 6953 | 489110 | 1.01 (1-1.01) | 3.34E-03 | 1.07E-02 |
| 454.1 | Varicose veins of lower extremity | circulatory system | 6145 | 473949 | 0.99 (0.99-1) | 3.49E-03 | 1.12E-02 |
| 555.1 | Regional enteritis | digestive | 2094 | 387662 | 1.01 (1-1.02) | 3.56E-03 | 1.13E-02 |
| 313.3 | Autism | mental disorders | 246 | 500570 | 0.97 (0.95-0.99) | 3.58E-03 | 1.14E-02 |
| 282.5 | Sickle cell anemia | hematopoietic | 450 | 460320 | 1.02 (1.01-1.04) | 3.83E-03 | 1.21E-02 |
| 334.2 | Anterior horn cell disease | neurological | 688 | 474382 | 0.98 (0.97-0.99) | 3.87E-03 | 1.22E-02 |
| 454.11 | Varicose veins of lower extremity, symptomtic | circulatory system | 1809 | 469613 | 1.01 (1-1.02) | 3.91E-03 | 1.23E-02 |
| 368.4 | Visual field defects | sense organs | 1060 | 495171 | 1.01 (1-1.02) | 4.30E-03 | 1.35E-02 |
| 574.12 | Cholelithiasis with other cholecystitis | digestive | 6852 | 485497 | 1.01 (1-1.01) | 4.44E-03 | 1.39E-02 |
| 117.4 | Aspergillosis | infectious diseases | 386 | 495064 | 1.02 (1.01-1.04) | 4.73E-03 | 1.48E-02 |
| 798 | Malaise and fatigue | symptoms | 8833 | 501483 | 1 (1-1.01) | 5.02E-03 | 1.56E-02 |
| 315.1 | Learning disorder | mental disorders | 322 | 500646 | 0.97 (0.96-0.99) | 5.03E-03 | 1.56E-02 |
| 569 | Other disorders of intestine | digestive | 7041 | 473768 | 1.01 (1-1.01) | 5.16E-03 | 1.60E-02 |
| 296.1 | Bipolar | mental disorders | 1643 | 408131 | 1.01 (1-1.02) | 5.24E-03 | 1.62E-02 |
| 227.3 | Benign neoplasm of pituitary gland and craniopharyngeal duct (pouch) | neoplasms | 496 | 500219 | 0.98 (0.97-0.99) | 5.29E-03 | 1.63E-02 |
| 38.2 | Gram positive septicemia | infectious diseases | 1216 | 471745 | 1.01 (1-1.02) | 5.55E-03 | 1.70E-02 |
| 618.2 | Uterine/Uterovaginal prolapse | genitourinary | 4577 | 496166 | 0.99 (0.99-1) | 5.56E-03 | 1.70E-02 |
| 705.8 | Hyperhidrosis | dermatologic | 1154 | 496598 | 1.01 (1-1.02) | 5.59E-03 | 1.70E-02 |
| 949 | Allergies, other | injuries & poisonings | 622 | 493062 | 1.02 (1.01-1.03) | 5.71E-03 | 1.74E-02 |
| 624.9 | stress incontinence, female | genitourinary | 3552 | 500344 | 0.99 (0.99-1) | 5.77E-03 | 1.75E-02 |
| 850 | Hemorrhage or hematoma complicating a procedure | injuries & poisonings | 7366 | 480408 | 1.01 (1-1.01) | 5.90E-03 | 1.78E-02 |
| 686 | Other local infections of skin and subcutaneous tissue | dermatologic | 2982 | 488407 | 1.01 (1-1.01) | 5.96E-03 | 1.80E-02 |
| 793.2 | Nonspecific abnormal findings on radiological and other examination of other intrathoracic organs (echocardiogram, etc) | symptoms | 1650 | 502490 | 1.01 (1-1.02) | 5.98E-03 | 1.80E-02 |
| 509.5 | Respiratory arrest | respiratory | 328 | 465927 | 1.02 (1.01-1.04) | 6.06E-03 | 1.82E-02 |
| 10 | Tuberculosis | infectious diseases | 283 | 470812 | 1.03 (1.01-1.04) | 6.10E-03 | 1.82E-02 |
| 198 | Secondary malignant neoplasm | neoplasms | 4064 | 438425 | 1.01 (1-1.01) | 6.12E-03 | 1.82E-02 |
| 145.2 | Cancer of tongue | neoplasms | 517 | 499998 | 1.02 (1.01-1.03) | 6.29E-03 | 1.87E-02 |
| 227.1 | Benign neoplasm of adrenal gland | neoplasms | 572 | 500295 | 1.02 (1-1.03) | 6.83E-03 | 2.02E-02 |
| 721 | Spondylosis and allied disorders | musculoskeletal | 8258 | 478960 | 1 (0.99-1) | 7.00E-03 | 2.07E-02 |
| 250.6 | Polyneuropathy in diabetes | endocrine/metabolic | 1353 | 461707 | 0.99 (0.98-1) | 7.18E-03 | 2.12E-02 |
| 696.42 | Psoriatic arthropathy | dermatologic | 1170 | 482547 | 1.01 (1-1.02) | 7.41E-03 | 2.18E-02 |
| 426.2 | Atrioventricular [AV] block | circulatory system | 458 | 449859 | 1.02 (1-1.03) | 7.84E-03 | 2.30E-02 |
| 595 | Hydronephrosis | genitourinary | 5259 | 496087 | 1.01 (1-1.01) | 7.96E-03 | 2.33E-02 |
| 446.9 | Arteritis NOS | circulatory system | 610 | 482501 | 1.02 (1-1.03) | 7.99E-03 | 2.33E-02 |
| 250.42 | Other abnormal glucose | endocrine/metabolic | 982 | 461336 | 1.01 (1-1.02) | 8.02E-03 | 2.33E-02 |
| 733.4 | Aseptic necrosis of bone | musculoskeletal | 735 | 474708 | 1.02 (1-1.03) | 8.10E-03 | 2.35E-02 |
| 256.4 | Polycystic ovaries | endocrine/metabolic | 248 | 493425 | 1.03 (1.01-1.05) | 8.74E-03 | 2.53E-02 |
| 575.2 | Obstruction of bile duct | digestive | 1887 | 480532 | 1.01 (1-1.02) | 8.78E-03 | 2.53E-02 |
| 591 | Urinary tract infection | genitourinary | 23909 | 486970 | 1 (1-1) | 9.11E-03 | 2.62E-02 |
| 149.4 | Cancer of larynx | neoplasms | 386 | 499867 | 1.02 (1-1.03) | 1.07E-02 | 3.07E-02 |
| 316.1 | Polyneuropathy due to drugs | mental disorders | 329 | 455347 | 1.02 (1.01-1.04) | 1.08E-02 | 3.09E-02 |
| 599.2 | Retention of urine | genitourinary | 17978 | 481753 | 1 (0.99-1) | 1.10E-02 | 3.13E-02 |
| 289.5 | Diseases of spleen | hematopoietic | 1207 | 488065 | 1.01 (1-1.02) | 1.12E-02 | 3.17E-02 |
| 352.1 | Trigeminal nerve disorders [CN5] | neurological | 907 | 486410 | 0.99 (0.98-1) | 1.13E-02 | 3.19E-02 |
| 1015 | Effects of other external causes | NULL | 4959 | 502490 | 1.01 (1-1.01) | 1.13E-02 | 3.19E-02 |
| 368 | Visual disturbances | sense organs | 2338 | 496449 | 1.01 (1-1.01) | 1.14E-02 | 3.22E-02 |
| 362.29 | Macular degeneration (senile) of retina NOS | sense organs | 10137 | 477692 | 1 (0.99-1) | 1.16E-02 | 3.27E-02 |
| 714 | Rheumatoid arthritis and other inflammatory polyarthropathies | musculoskeletal | 1367 | 486349 | 1.01 (1-1.02) | 1.17E-02 | 3.28E-02 |
| 597 | Other disorders of urethra and urinary tract | genitourinary | 945 | 482628 | 0.99 (0.98-1) | 1.18E-02 | 3.29E-02 |
| 361.1 | Retinal detachment with retinal defect | sense organs | 2185 | 472358 | 1.01 (1-1.02) | 1.18E-02 | 3.29E-02 |
| 735.21 | Hammer toe (acquired) | musculoskeletal | 2363 | 484616 | 0.99 (0.98-1) | 1.21E-02 | 3.36E-02 |
| 8.6 | Viral Enteritis | infectious diseases | 1767 | 498829 | 1.01 (1-1.02) | 1.21E-02 | 3.36E-02 |
| 198.4 | Secondary malignant neoplasm of liver | neoplasms | 7632 | 441993 | 1 (1-1.01) | 1.22E-02 | 3.38E-02 |
| 618.5 | Prolapse of vaginal vault after hysterectomy | genitourinary | 433 | 492022 | 0.98 (0.96-1) | 1.22E-02 | 3.38E-02 |
| 716.9 | Arthropathy NOS | musculoskeletal | 81241 | 495365 | 1 (1-1) | 1.25E-02 | 3.45E-02 |
| 561.1 | Diarrhea | digestive | 1363 | 502490 | 1.01 (1-1.02) | 1.26E-02 | 3.47E-02 |
| 1011 | Complications of surgical and medical procedures | NULL | 5557 | 502490 | 1.01 (1-1.01) | 1.28E-02 | 3.52E-02 |
| 282.8 | Other hemoglobinopathies | hematopoietic | 441 | 460311 | 1.02 (1-1.03) | 1.32E-02 | 3.60E-02 |
| 622.1 | Polyp of corpus uteri | genitourinary | 5420 | 500459 | 1.01 (1-1.01) | 1.36E-02 | 3.72E-02 |
| 735.3 | Hallux valgus (Bunion) | musculoskeletal | 5945 | 488198 | 0.99 (0.99-1) | 1.39E-02 | 3.77E-02 |
| 737.1 | Kyphosis (acquired) | musculoskeletal | 816 | 483069 | 0.99 (0.97-1) | 1.50E-02 | 4.07E-02 |
| 1002 | Symptoms concerning nutrition, metabolism, and development | NULL | 14288 | 502490 | 1 (1-1.01) | 1.52E-02 | 4.11E-02 |
| 704 | Diseases of hair and hair follicles | dermatologic | 3204 | 501123 | 0.99 (0.99-1) | 1.56E-02 | 4.20E-02 |
| 277 | Other disorders of metabolism | endocrine/metabolic | 4098 | 500960 | 1.01 (1-1.01) | 1.61E-02 | 4.32E-02 |
| 760 | Back pain | symptoms | 19696 | 502490 | 1 (0.99-1) | 1.62E-02 | 4.34E-02 |
| 501 | Pneumonitis due to inhalation of food or vomitus | respiratory | 3932 | 469531 | 1.01 (1-1.01) | 1.74E-02 | 4.65E-02 |
| 189.2 | Cancer of bladder | neoplasms | 2347 | 497062 | 1.01 (1-1.01) | 1.74E-02 | 4.66E-02 |
| 627.1 | Postmenopausal bleeding | genitourinary | 5564 | 494016 | 1.01 (1-1.01) | 1.75E-02 | 4.66E-02 |
| 433.3 | Cerebral ischemia | circulatory system | 6436 | 483495 | 1 (1-1.01) | 1.78E-02 | 4.73E-02 |
| - | Genitourinary mortality | - | 210 | 210 | 1.05 (1.05-1.06) | 5.80E-97 | 5.10E-95 |
| - | Circulatory mortality | - | 207 | 207 | 1.02 (1.02-1.03) | 1.23E-81 | 8.65E-80 |
| - | All cause mortality | - | 201 | 201 | 1.01 (1.01-1.01) | 7.45E-34 | 1.79E-32 |
| - | Respiratory mortality | - | 208 | 208 | 1.01 (1.01-1.02) | 2.25E-19 | 3.44E-18 |
| - | Neurological mortality | - | 206 | 206 | 0.98 (0.97-0.98) | 9.90E-16 | 1.16E-14 |
| - | Digestive mortality | - | 209 | 209 | 1.02 (1.01-1.02) | 8.73E-11 | 8.15E-10 |
| - | Dermatologic mortality | - | 211 | 211 | 1.04 (1.03-1.06) | 1.36E-10 | 1.24E-09 |
| - | Endocrine/metabolic mortality | - | 203 | 203 | 1.01 (1.01-1.01) | 2.21E-06 | 1.34E-05 |
| - | Hematopoietic mortality | - | 204 | 204 | 1.02 (1.01-1.04) | 1.38E-04 | 6.03E-04 |
| - | Musculoskeletal mortality | - | 212 | 212 | 1.02 (1.01-1.03) | 2.49E-04 | 1.04E-03 |
| - | Neoplasms mortality | - | 202 | 202 | 1 (1-1.01) | 3.26E-04 | 1.33E-03 |

**Supplementary Table 3. Significant phenotypic associations identified by PRS-based PheWAS.**

| **Phenotype** | **Description** | **Group** | **Cases** | **Participants** | **Model 1** | | | **Model 2** | | |
| --- | --- | --- | --- | --- | --- | --- | --- | --- | --- | --- |
|  |  |  |  |  | **OR (95% CI)** | **P value** | **FDR** | **OR (95% CI)** | **P value** | **FDR** |
| 274.1 | Gout | endocrine/metabolic | 8329 | 384723 | 3.35 (3.18, 3.54) | 0 | 0 | 3.31 (3.13, 3.51) | 0 | 0 |
| 557.1 | Celiac disease | digestive | 2715 | 280081 | 2.07 (1.88, 2.27) | 6.18E-53 | 3.30E-50 | 2.09 (1.89, 2.29) | 4.73E-51 | 2.49E-48 |
| 401.1 | Essential hypertension | circulatory system | 120577 | 385509 | 1.09 (1.07, 1.11) | 1.67E-22 | 5.94E-20 | 1.09 (1.07, 1.11) | 3.38E-21 | 1.19E-18 |
| 274.11 | Gouty arthropathy | endocrine/metabolic | 277 | 376671 | 4.05 (3.03, 5.40) | 2.58E-21 | 6.88E-19 | 4.00 (2.99, 5.37) | 1.81E-20 | 4.77E-18 |
| 411.2 | Myocardial infarction | circulatory system | 21028 | 355664 | 1.13 (1.09, 1.17) | 8.61E-12 | 1.84E-09 | 1.12 (1.08, 1.17) | 1.81E-10 | 3.82E-08 |
| 585.3 | Chronic renal failure [CKD] | genitourinary | 9051 | 362666 | 1.19 (1.13, 1.25) | 8.64E-11 | 1.54E-08 | 1.18 (1.12, 1.24) | 1.55E-09 | 2.33E-07 |
| 244.4 | Hypothyroidism NOS | endocrine/metabolic | 23427 | 380534 | 1.11 (1.07, 1.15) | 8.86E-10 | 1.35E-07 | 1.11 (1.07, 1.15) | 1.24E-09 | 2.18E-07 |
| 600 | Hyperplasia of prostate | genitourinary | 22261 | 371785 | 0.90 (0.87, 0.93) | 5.28E-09 | 7.04E-07 | 0.90 (0.87, 0.93) | 1.26E-08 | 1.66E-06 |
| 411.4 | Coronary atherosclerosis | circulatory system | 31658 | 366294 | 1.09 (1.06, 1.12) | 1.35E-08 | 1.60E-06 | 1.08 (1.05, 1.11) | 1.27E-06 | 1.22E-04 |
| 411.3 | Angina pectoris | circulatory system | 23174 | 357810 | 1.10 (1.07, 1.14) | 1.80E-08 | 1.92E-06 | 1.09 (1.05, 1.13) | 8.50E-07 | 9.95E-05 |
| 272.11 | Hypercholesterolemia | endocrine/metabolic | 56815 | 380877 | 1.07 (1.04, 1.09) | 4.27E-08 | 4.14E-06 | 1.06 (1.04, 1.08) | 1.14E-06 | 1.20E-04 |
| 411.8 | Other chronic ischemic heart disease, unspecified | circulatory system | 26547 | 361183 | 1.08 (1.05, 1.12) | 6.53E-07 | 5.81E-05 | 1.08 (1.04, 1.11) | 1.49E-05 | 1.21E-03 |
| 443.9 | Peripheral vascular disease, unspecified | circulatory system | 5356 | 372974 | 1.18 (1.11, 1.26) | 9.87E-07 | 8.10E-05 | 1.16 (1.09, 1.25) | 1.74E-05 | 1.31E-03 |
| 459.9 | Circulatory disease NEC | circulatory system | 33770 | 372986 | 1.07 (1.04, 1.10) | 1.76E-06 | 1.34E-04 | 1.07 (1.04, 1.10) | 1.61E-06 | 1.41E-04 |
| 185 | Cancer of prostate | neoplasms | 14176 | 363700 | 0.91 (0.87, 0.95) | 2.61E-05 | 1.74E-03 | 0.92 (0.88, 0.96) | 1.93E-04 | 8.87E-03 |
| 714.1 | Rheumatoid arthritis | musculoskeletal | 7601 | 377548 | 1.13 (1.07, 1.19) | 2.98E-05 | 1.87E-03 | 1.12 (1.06, 1.19) | 8.42E-05 | 4.93E-03 |
| 580.2 | Nephrotic syndrome without mention of glomerulonephritis | genitourinary | 987 | 354602 | 1.38 (1.19, 1.61) | 3.71E-05 | 2.20E-03 | 1.34 (1.14, 1.57) | 3.20E-04 | 1.16E-02 |
| 717 | Polymyalgia Rheumatica | musculoskeletal | 3558 | 385799 | 1.18 (1.09, 1.28) | 6.44E-05 | 3.62E-03 | 1.19 (1.09, 1.29) | 7.56E-05 | 4.69E-03 |
| 276.13 | Hyperpotassemia | endocrine/metabolic | 3365 | 363395 | 1.18 (1.09, 1.28) | 1.01E-04 | 5.39E-03 | 1.19 (1.09, 1.29) | 1.09E-04 | 5.57E-03 |
| 525 | Other diseases of the teeth and supporting structures | digestive | 3918 | 375337 | 1.17 (1.08, 1.26) | 1.10E-04 | 5.61E-03 | 1.17 (1.08, 1.27) | 1.11E-04 | 5.57E-03 |
| 285 | Other anemias | hematopoietic | 23261 | 371462 | 1.07 (1.03, 1.10) | 1.40E-04 | 6.77E-03 | 1.06 (1.03, 1.10) | 2.80E-04 | 1.16E-02 |
| 250.2 | Type 2 diabetes | endocrine/metabolic | 31332 | 382372 | 1.06 (1.03, 1.09) | 1.46E-04 | 6.79E-03 | 1.05 (1.02, 1.08) | 1.16E-03 | 2.99E-02 |
| 550.2 | Diaphragmatic hernia | digestive | 43176 | 360120 | 0.95 (0.93, 0.98) | 1.73E-04 | 7.70E-03 | 0.95 (0.93, 0.98) | 1.05E-04 | 5.57E-03 |
| 433.31 | Transient cerebral ischemia | circulatory system | 3771 | 367253 | 1.16 (1.07, 1.26) | 2.10E-04 | 8.95E-03 | 1.17 (1.08, 1.27) | 1.45E-04 | 6.95E-03 |
| 10 | Tuberculosis | infectious diseases | 248 | 356046 | 1.78 (1.31, 2.43) | 2.25E-04 | 9.13E-03 | 1.80 (1.31, 2.47) | 3.10E-04 | 1.16E-02 |
| 433.2 | Occlusion of cerebral arteries | circulatory system | 6732 | 370214 | 1.12 (1.05, 1.19) | 2.31E-04 | 9.13E-03 | 1.13 (1.07, 1.20) | 6.69E-05 | 4.41E-03 |
| 278.1 | Obesity | endocrine/metabolic | 29927 | 385612 | 1.06 (1.03, 1.09) | 2.40E-04 | 9.15E-03 | 1.05 (1.02, 1.09) | 5.56E-04 | 1.78E-02 |
| 454.1 | Varicose veins of lower extremity | circulatory system | 13141 | 352502 | 0.92 (0.89, 0.97) | 3.56E-04 | 1.28E-02 | 0.92 (0.88, 0.96) | 3.48E-04 | 1.22E-02 |
| 414 | Other forms of chronic heart disease | circulatory system | 7426 | 342062 | 1.11 (1.05, 1.18) | 3.59E-04 | 1.28E-02 | 1.10 (1.04, 1.17) | 1.11E-03 | 2.92E-02 |
| 369 | Infection of the eye | sense organs | 399 | 371205 | 1.54 (1.21, 1.96) | 4.81E-04 | 1.56E-02 | 1.55 (1.21, 2.00) | 5.74E-04 | 1.78E-02 |
| 441.1 | Acute vascular insufficiency of intestine | circulatory system | 701 | 368319 | 1.38 (1.15, 1.66) | 4.89E-04 | 1.56E-02 | 1.39 (1.15, 1.67) | 6.21E-04 | 1.82E-02 |
| 585.1 | Acute renal failure | genitourinary | 19453 | 373068 | 1.07 (1.03, 1.11) | 4.94E-04 | 1.56E-02 | 1.07 (1.03, 1.11) | 3.04E-04 | 1.16E-02 |
| 602 | Other disorders of prostate | genitourinary | 2073 | 351597 | 0.83 (0.74, 0.92) | 4.96E-04 | 1.56E-02 | 0.82 (0.74, 0.92) | 6.04E-04 | 1.82E-02 |
| 433.21 | Cerebral artery occlusion, with cerebral infarction | circulatory system | 5150 | 368632 | 1.13 (1.05, 1.21) | 5.40E-04 | 1.65E-02 | 1.14 (1.06, 1.22) | 2.77E-04 | 1.16E-02 |
| 428.2 | Heart failure NOS | circulatory system | 12430 | 381472 | 1.08 (1.03, 1.13) | 5.72E-04 | 1.70E-02 | 1.09 (1.04, 1.14) | 4.71E-04 | 1.60E-02 |
| 781 | Symptoms involving nervous and musculoskeletal systems | symptoms | 4215 | 385793 | 1.14 (1.06, 1.23) | 7.47E-04 | 2.16E-02 | 1.15 (1.06, 1.24) | 5.46E-04 | 1.78E-02 |
| 443.1 | Raynaud's syndrome | circulatory system | 2680 | 370298 | 1.17 (1.07, 1.29) | 7.81E-04 | 2.19E-02 | 1.17 (1.06, 1.29) | 1.37E-03 | 3.44E-02 |
| 250.1 | Type 1 diabetes | endocrine/metabolic | 3441 | 354481 | 1.15 (1.06, 1.25) | 8.18E-04 | 2.24E-02 | 1.14 (1.05, 1.24) | 2.32E-03 | 4.72E-02 |
| 772 | Symptoms of the muscles | symptoms | 1358 | 385496 | 1.25 (1.10, 1.43) | 8.42E-04 | 2.25E-02 | 1.23 (1.08, 1.41) | 2.25E-03 | 4.72E-02 |
| 711.1 | Pyogenic arthritis | musculoskeletal | 614 | 310817 | 1.39 (1.14, 1.69) | 9.78E-04 | 2.55E-02 | 1.36 (1.11, 1.66) | 2.72E-03 | 5.32E-02 |
| 480 | Pneumonia | respiratory | 12410 | 370035 | 1.08 (1.03, 1.13) | 1.05E-03 | 2.66E-02 | 1.07 (1.02, 1.12) | 5.33E-03 | 8.39E-02 |
| 440.2 | Atherosclerosis of the extremities | circulatory system | 1657 | 369275 | 1.22 (1.08, 1.37) | 1.24E-03 | 3.07E-02 | 1.19 (1.06, 1.35) | 4.61E-03 | 8.21E-02 |
| 585.31 | Renal dialysis | genitourinary | 913 | 354528 | 1.30 (1.11, 1.53) | 1.37E-03 | 3.32E-02 | 1.29 (1.10, 1.53) | 2.33E-03 | 4.72E-02 |
| 276.12 | Hyposmolality and/or hyponatremia | endocrine/metabolic | 8057 | 368087 | 1.09 (1.04, 1.16) | 1.40E-03 | 3.32E-02 | 1.11 (1.05, 1.17) | 3.15E-04 | 1.16E-02 |
| 428.1 | Congestive heart failure (CHF) NOS | circulatory system | 6662 | 375704 | 1.10 (1.04, 1.17) | 1.45E-03 | 3.37E-02 | 1.10 (1.04, 1.17) | 2.16E-03 | 4.65E-02 |
| 211 | Benign neoplasm of other parts of digestive system | neoplasms | 11465 | 369681 | 0.93 (0.89, 0.97) | 1.53E-03 | 3.48E-02 | 0.93 (0.89, 0.97) | 2.16E-03 | 4.65E-02 |
| 41.1 | Staphylococcus infections | infectious diseases | 5398 | 361196 | 1.11 (1.04, 1.19) | 1.63E-03 | 3.59E-02 | 1.12 (1.05, 1.20) | 9.83E-04 | 2.73E-02 |
| 598 | Abnormal findings on examination of urine | genitourinary | 4581 | 385799 | 0.89 (0.83, 0.96) | 1.65E-03 | 3.59E-02 | 0.89 (0.83, 0.96) | 2.11E-03 | 4.65E-02 |
| 443.7 | Peripheral angiopathy in diseases classified elsewhere | circulatory system | 892 | 368510 | 1.29 (1.10, 1.52) | 1.85E-03 | 3.94E-02 | 1.27 (1.08, 1.50) | 4.93E-03 | 8.27E-02 |
| 599 | Other symptoms/disorders or the urinary system | genitourinary | 11899 | 357375 | 0.93 (0.89, 0.97) | 2.11E-03 | 4.42E-02 | 0.92 (0.88, 0.97) | 1.08E-03 | 2.91E-02 |
| 550.1 | Inguinal hernia | digestive | 21881 | 338825 | 0.95 (0.91, 0.98) | 2.20E-03 | 4.50E-02 | 0.94 (0.91, 0.98) | 9.25E-04 | 2.63E-02 |
| 411.1 | Unstable angina (intermediate coronary syndrome) | circulatory system | 6291 | 340927 | 1.10 (1.03, 1.17) | 2.41E-03 | 4.86E-02 | 1.09 (1.03, 1.17) | 6.18E-03 | 8.81E-02 |
| - | Circulatory mortality | - | 16425 | 385799 | 1.11 (1.06, 1.16) | 5.98E-06 | 4.25E-04 | 1.10 (1.05, 1.15) | 5.38E-05 | 3.78E-03 |

Note: Model 1 was adjusted for age, sex, assessment center and the first 10 PCs, model 2 was adjusted for year of birth, sex, place of birth (both north and east coordinates) and the first 10 PCs.

**Supplementary Table 4. Linear and non-linear associations of uric acid levels with risk of overlapping disease outcomes.**

| **Phenotype** | **Description** | **Cases** | **OR (95% CI)** | **Linear P** | **Non-linear P** |
| --- | --- | --- | --- | --- | --- |
| 274.1 | Gout | 8329 | 3.35 (3.18, 3.54) | 0 | 0.004 |
| 557.1 | Celiac disease | 2715 | 2.07 (1.88, 2.27) | 6.18E-53 | 0.001 |
| 401.1 | Essential hypertension | 120577 | 1.09 (1.07, 1.11) | 1.67E-22 | 0.500 |
| 274.11 | Gouty arthropathy | 277 | 4.05 (3.03, 5.40) | 2.58E-21 | 0.521 |
| 411.2 | Myocardial infarction | 21028 | 1.13 (1.09, 1.17) | 8.61E-12 | 0.087 |
| 585.3 | Chronic renal failure [CKD] | 9051 | 1.19 (1.13, 1.25) | 8.64E-11 | 0.779 |
| 244.4 | Hypothyroidism NOS | 23427 | 1.11 (1.07, 1.15) | 8.86E-10 | 0.297 |
| 600 | Hyperplasia of prostate | 22261 | 0.90 (0.87, 0.93) | 5.28E-09 | - |
| 411.4 | Coronary atherosclerosis | 31658 | 1.09 (1.06, 1.12) | 1.35E-08 | 0.834 |
| 411.3 | Angina pectoris | 23174 | 1.10 (1.07, 1.14) | 1.80E-08 | 0.200 |
| 272.11 | Hypercholesterolemia | 56815 | 1.07 (1.04, 1.09) | 4.27E-08 | 0.887 |
| 411.8 | Other chronic ischemic heart disease, unspecified | 26547 | 1.08 (1.05, 1.12) | 6.53E-07 | 0.319 |
| 443.9 | Peripheral vascular disease, unspecified | 5356 | 1.18 (1.11, 1.26) | 9.87E-07 | 0.118 |
| 459.9 | Circulatory disease NEC | 33770 | 1.07 (1.04, 1.10) | 1.76E-06 | 0.734 |
| 580.2 | Nephrotic syndrome without mention of glomerulonephritis | 987 | 1.38 (1.19, 1.61) | 3.71E-05 | 0.725 |
| 717 | Polymyalgia Rheumatica | 3558 | 1.18 (1.09, 1.28) | 6.44E-05 | 0.074 |
| 276.13 | Hyperpotassemia | 3365 | 1.18 (1.09, 1.28) | 1.01E-04 | 0.606 |
| 285 | Other anemias | 23261 | 1.07 (1.03, 1.10) | 1.40E-04 | 0.068 |
| 250.2 | Type 2 diabetes | 31332 | 1.06 (1.03, 1.09) | 1.46E-04 | 0.994 |
| 433.31 | Transient cerebral ischemia | 3771 | 1.16 (1.07, 1.26) | 2.10E-04 | 0.110 |
| 10 | Tuberculosis | 248 | 1.78 (1.31, 2.43) | 2.25E-04 | 0.362 |
| 433.2 | Occlusion of cerebral arteries | 6732 | 1.12 (1.05, 1.19) | 2.31E-04 | 0.865 |
| 278.1 | Obesity | 29927 | 1.06 (1.03, 1.09) | 2.40E-04 | 0.627 |
| 454.1 | Varicose veins of lower extremity | 13141 | 0.92 (0.89, 0.97) | 3.56E-04 | 0.631 |
| 414 | Other forms of chronic heart disease | 7426 | 1.11 (1.05, 1.18) | 3.59E-04 | 0.341 |
| 441.1 | Acute vascular insufficiency of intestine | 701 | 1.38 (1.15, 1.66) | 4.89E-04 | 0.156 |
| 585.1 | Acute renal failure | 19453 | 1.07 (1.03, 1.11) | 4.94E-04 | 0.118 |
| 433.21 | Cerebral artery occlusion, with cerebral infarction | 5150 | 1.13 (1.05, 1.21) | 5.40E-04 | 0.211 |
| 428.2 | Heart failure NOS | 12430 | 1.08 (1.03, 1.13) | 5.72E-04 | 0.737 |
| 711.1 | Pyogenic arthritis | 614 | 1.39 (1.14, 1.69) | 9.78E-04 | 0.382 |
| 480 | Pneumonia | 12410 | 1.08 (1.03, 1.13) | 1.05E-03 | 0.321 |
| 440.2 | Atherosclerosis of the extremities | 1657 | 1.22 (1.08, 1.37) | 1.24E-03 | 0.287 |
| 585.31 | Renal dialysis | 913 | 1.30 (1.11, 1.53) | 1.37E-03 | 0.786 |
| 428.1 | Congestive heart failure (CHF) NOS | 6662 | 1.10 (1.04, 1.17) | 1.45E-03 | 0.493 |
| 211 | Benign neoplasm of other parts of digestive system | 11465 | 0.93 (0.89, 0.97) | 1.53E-03 | 0.038 |
| 41.1 | Staphylococcus infections | 5398 | 1.11 (1.04, 1.19) | 1.63E-03 | 0.425 |
| 598 | Abnormal findings on examination of urine | 4581 | 0.89 (0.83, 0.96) | 1.65E-03 | 0.312 |
| 599 | Other symptoms/disorders or the urinary system | 11899 | 0.93 (0.89, 0.97) | 2.11E-03 | 0.270 |
| 550.1 | Inguinal hernia | 21881 | 0.95 (0.91, 0.98) | 2.20E-03 | 0.700 |
| 411.1 | Unstable angina (intermediate coronary syndrome) | 6291 | 1.10 (1.03, 1.17) | 2.41E-03 | 0.177 |
| - | Circulatory mortality | 16425 | 1.11 (1.06, 1.16) | 5.98E-06 | 0.411 |

“-” in the non-linear P column represents that the model can not be fitted.

**Supplementary Table 5. Associations of uric acid levels with risk of overlapping disease outcomes, stratified by gender.**

| **Phenotype** | **Description** | **Male subgroup** | | | **Female subgroup** | | | **Heterogeneity P value** |
| --- | --- | --- | --- | --- | --- | --- | --- | --- |
|  |  | **Cases** | **OR (95% CI)** | **P value** | **Cases** | **OR (95% CI)** | **P value** |  |
| 274.1 | Gout | 7017 | 3.42 (3.22, 3.63) | 0 | 1310 | 3.01 (2.64, 3.44) | 8.28E-59 | 0.089 |
| 557.1 | Celiac disease | 965 | 2.03 (1.73, 2.37) | 6.10E-19 | 1750 | 2.09 (1.86, 2.35) | 1.20E-35 | 0.757 |
| 401.1 | Essential hypertension | 64225 | 1.08 (1.05, 1.10) | 2.10E-08 | 56400 | 1.11 (1.08, 1.14) | 2.53E-16 | 0.073 |
| 274.11 | Gouty arthropathy | 239 | 4.48 (3.29, 6.12) | 3.07E-21 | 38 | 2.14 (0.97, 4.71) | 5.79E-02 | 0.087 |
| 411.2 | Myocardial infarction | 15311 | 1.15 (1.10, 1.20) | 1.51E-10 | 5717 | 1.09 (1.03, 1.17) | 6.82E-03 | 0.240 |
| 585.3 | Chronic renal failure [CKD] | 5238 | 1.18 (1.10, 1.26) | 3.84E-06 | 3810 | 1.20 (1.11, 1.30) | 4.58E-06 | 0.659 |
| 244.4 | Hypothyroidism NOS | 4863 | 1.11 (1.03, 1.19) | 4.13E-03 | 18600 | 1.11 (1.07, 1.15) | 5.63E-08 | 0.987 |
| 600 | Hyperplasia of prostate | 22261 | 0.90 (0.87, 0.93) | 5.27E-09 | - | - | - | - |
| 411.4 | Coronary atherosclerosis | 23086 | 1.09 (1.05, 1.13) | 1.22E-06 | 8572 | 1.09 (1.03, 1.15) | 2.82E-03 | 0.889 |
| 411.3 | Angina pectoris | 14938 | 1.12 (1.07, 1.17) | 3.64E-07 | 8236 | 1.08 (1.02, 1.14) | 7.89E-03 | 0.315 |
| 272.11 | Hypercholesterolemia | 33099 | 1.07 (1.04, 1.10) | 1.97E-05 | 23716 | 1.06 (1.03, 1.10) | 5.47E-04 | 0.797 |
| 411.8 | Other chronic ischemic heart disease, unspecified | 18539 | 1.10 (1.06, 1.15) | 5.44E-07 | 8008 | 1.04 (0.99, 1.10) | 1.27E-01 | 0.107 |
| 443.9 | Peripheral vascular disease, unspecified | 3658 | 1.18 (1.09, 1.28) | 5.85E-05 | 1698 | 1.18 (1.05, 1.33) | 5.39E-03 | 0.992 |
| 459.9 | Circulatory disease NEC | 18372 | 1.06 (1.02, 1.10) | 2.71E-03 | 15398 | 1.08 (1.04, 1.13) | 1.36E-04 | 0.465 |
| 580.2 | Nephrotic syndrome without mention of glomerulonephritis | 621 | 1.35 (1.12, 1.65) | 2.24E-03 | 366 | 1.44 (1.12, 1.85) | 4.83E-03 | 0.712 |
| 717 | Polymyalgia Rheumatica | 1354 | 1.29 (1.13, 1.47) | 1.91E-04 | 2204 | 1.12 (1.01, 1.24) | 3.31E-02 | 0.106 |
| 276.13 | Hyperpotassemia | 2121 | 1.16 (1.04, 1.29) | 5.72E-03 | 1244 | 1.22 (1.06, 1.40) | 4.88E-03 | 0.586 |
| 285 | Other anemias | 10727 | 1.10 (1.05, 1.15) | 1.79E-04 | 12534 | 1.04 (0.99, 1.09) | 8.70E-02 | 0.109 |
| 250.2 | Type 2 diabetes | 18880 | 1.05 (1.01, 1.09) | 8.49E-03 | 12452 | 1.07 (1.02, 1.12) | 5.28E-03 | 0.653 |
| 433.31 | Transient cerebral ischemia | 2149 | 1.20 (1.08, 1.33) | 8.63E-04 | 1622 | 1.12 (0.99, 1.26) | 6.68E-02 | 0.418 |
| 10 | Tuberculosis | 149 | 2.03 (1.36, 3.01) | 4.80E-04 | 99 | 1.47 (0.90, 2.39) | 1.23E-01 | 0.314 |
| 433.2 | Occlusion of cerebral arteries | 4105 | 1.10 (1.02, 1.19) | 1.23E-02 | 2627 | 1.14 (1.04, 1.26) | 5.59E-03 | 0.556 |
| 278.1 | Obesity | 14154 | 1.08 (1.04, 1.13) | 3.91E-04 | 15773 | 1.04 (0.99, 1.08) | 8.68E-02 | 0.161 |
| 454.1 | Varicose veins of lower extremity | 4598 | 0.89 (0.83, 0.96) | 2.17E-03 | 8543 | 0.94 (0.89, 0.99) | 2.87E-02 | 0.244 |
| 414 | Other forms of chronic heart disease | 5011 | 1.11 (1.04, 1.19) | 3.47E-03 | 2415 | 1.11 (1.01, 1.23) | 3.76E-02 | 0.995 |
| 441.1 | Acute vascular insufficiency of intestine | 359 | 1.45 (1.12, 1.87) | 4.65E-03 | 342 | 1.32 (1.02, 1.72) | 3.66E-02 | 0.630 |
| 585.1 | Acute renal failure | 11889 | 1.05 (1.01, 1.10) | 2.89E-02 | 7564 | 1.09 (1.03, 1.15) | 4.79E-03 | 0.424 |
| 433.21 | Cerebral artery occlusion, with cerebral infarction | 3117 | 1.05 (0.96, 1.15) | 2.59E-01 | 2033 | 1.25 (1.12, 1.40) | 4.24E-05 | 0.014 |
| 428.2 | Heart failure NOS | 8144 | 1.06 (1.00, 1.12) | 4.54E-02 | 4286 | 1.13 (1.04, 1.21) | 1.95E-03 | 0.194 |
| 711.1 | Pyogenic arthritis | 388 | 1.82 (1.43, 2.33) | 1.55E-06 | 226 | 0.87 (0.63, 1.20) | 3.85E-01 | 0.000 |
| 480 | Pneumonia | 7285 | 1.06 (1.00, 1.13) | 4.42E-02 | 5125 | 1.10 (1.03, 1.18) | 6.45E-03 | 0.442 |
| 440.2 | Atherosclerosis of the extremities | 1233 | 1.26 (1.09, 1.44) | 1.30E-03 | 424 | 1.11 (0.88, 1.41) | 3.68E-01 | 0.393 |
| 585.31 | Renal dialysis | 591 | 1.21 (0.99, 1.48) | 6.03E-02 | 322 | 1.47 (1.13, 1.93) | 4.83E-03 | 0.251 |
| 428.1 | Congestive heart failure (CHF) NOS | 4268 | 1.09 (1.01, 1.17) | 2.82E-02 | 2394 | 1.13 (1.02, 1.25) | 1.68E-02 | 0.561 |
| 211 | Benign neoplasm of other parts of digestive system | 4526 | 0.92 (0.86, 0.99) | 3.26E-02 | 6939 | 0.93 (0.88, 0.99) | 1.99E-02 | 0.846 |
| 41.1 | Staphylococcus infections | 3178 | 1.15 (1.06, 1.26) | 1.49E-03 | 2220 | 1.06 (0.96, 1.18) | 2.64E-01 | 0.235 |
| 598 | Abnormal findings on examination of urine | 3047 | 0.85 (0.78, 0.93) | 4.73E-04 | 1534 | 0.97 (0.86, 1.10) | 6.05E-01 | 0.106 |
| 599 | Other symptoms/disorders or the urinary system | 7745 | 0.91 (0.86, 0.96) | 8.17E-04 | 4154 | 0.97 (0.90, 1.05) | 4.99E-01 | 0.144 |
| 550.1 | Inguinal hernia | 20194 | 0.95 (0.92, 0.99) | 7.24E-03 | 1687 | 0.90 (0.80, 1.02) | 9.30E-02 | 0.422 |
| 411.1 | Unstable angina (intermediate coronary syndrome) | 4171 | 1.10 (1.02, 1.19) | 1.53E-02 | 2120 | 1.10 (0.99, 1.23) | 6.49E-02 | 0.938 |
| - | Circulatory mortality | 10995 | 1.08 (1.02, 1.14) | 8.00E-03 | 5430 | 1.17 (1.09, 1.27) | 4.84E-05 | 0.080 |

**Supplementary Table 6. Associations of uric acid levels with risk of kidney diseases, stratified by eGFR levels.**

| **Phenotype** | **Description** | **Population** | **Cases** | **Low-level eGFR** | | **High-level eGFR** | | **Heterogeneity P value** |
| --- | --- | --- | --- | --- | --- | --- | --- | --- |
|  |  |  |  | **OR (95% CI)** | **P value** | **OR (95% CI)** | **P value** |  |
| 580.2 | Nephrotic syndrome without mention of glomerulonephritis | Overall | 987 | 1.42 (1.18, 1.71) | 1.79E-04 | 1.17 (0.86, 1.58) | 0.322 | 0.275 |
| 585.1 | Acute renal failure | Overall | 19453 | 1.08 (1.03, 1.13) | 0.001 | 1.02 (0.96, 1.09) | 0.559 | 0.132 |
| 585.3 | Chronic renal failure [CKD] | Overall | 9051 | 1.16 (1.10, 1.23) | 3.54E-07 | 1.16 (1.01, 1.34) | 0.033 | 0.994 |
| 585.31 | Renal dialysis | Overall | 913 | 1.33 (1.11, 1.58) | 0.002 | 0.89 (0.55, 1.43) | 0.627 | 0.122 |
| 598 | Abnormal findings on examination of urine | Overall | 4581 | 0.93 (0.84, 1.02) | 0.123 | 0.87 (0.77, 0.97) | 0.017 | 0.403 |
| 599 | Other symptoms/disorders or the urinary system | Overall | 11899 | 0.94 (0.88, 1.00) | 0.040 | 0.93 (0.86, 1.00) | 0.047 | 0.852 |
| 580.2 | Nephrotic syndrome without mention of glomerulonephritis | Male | 621 | 1.43 (1.14, 1.79) | 0.002 | 1.03 (0.68, 1.56) | 0.890 | 0.174 |
| 585.1 | Acute renal failure | Male | 11889 | 1.07 (1.01, 1.13) | 0.027 | 1.01 (0.93, 1.10) | 0.836 | 0.273 |
| 585.3 | Chronic renal failure [CKD] | Male | 5238 | 1.14 (1.06, 1.24) | 0.001 | 1.22 (1.02, 1.47) | 0.032 | 0.517 |
| 585.31 | Renal dialysis | Male | 591 | 1.22 (0.98, 1.51) | 0.071 | 0.89 (0.47, 1.69) | 0.725 | 0.366 |
| 598 | Abnormal findings on examination of urine | Male | 3047 | 0.89 (0.80, 1.00) | 0.060 | 0.83 (0.72, 0.97) | 0.016 | 0.469 |
| 599 | Other symptoms/disorders or the urinary system | Male | 7745 | 0.92 (0.86, 1.00) | 0.038 | 0.91 (0.83, 0.99) | 0.039 | 0.749 |
| 580.2 | Nephrotic syndrome without mention of glomerulonephritis | Female | 366 | 1.41 (1.03, 1.93) | 0.031 | 1.36 (0.87, 2.14) | 0.182 | 0.895 |
| 585.1 | Acute renal failure | Female | 7564 | 1.11 (1.03, 1.19) | 0.007 | 1.03 (0.94, 1.14) | 0.508 | 0.285 |
| 585.3 | Chronic renal failure [CKD] | Female | 3813 | 1.19 (1.09, 1.30) | 1.24E-04 | 1.09 (0.88, 1.35) | 0.450 | 0.445 |
| 585.31 | Renal dialysis | Female | 322 | 1.56 (1.16, 2.12) | 0.004 | 0.89 (0.43, 1.82) | 0.748 | 0.155 |
| 598 | Abnormal findings on examination of urine | Female | 1534 | 1.01 (0.85, 1.20) | 0.941 | 0.92 (0.77, 1.11) | 0.401 | 0.507 |
| 599 | Other symptoms/disorders or the urinary system | Female | 4154 | 0.97 (0.87, 1.08) | 0.556 | 0.96 (0.86, 1.08) | 0.515 | 0.944 |

**Supplementary Table 7. Effect estimates for significant disease pairs identified by trajectory analysis.**

| **D1** | **Description** | **D2** | **Description** | **N_both** | **N_D1_D2** | **Beta** | **SE** | **P value** |
| --- | --- | --- | --- | --- | --- | --- | --- | --- |
| 250.2 | Type 2 diabetes | 285 | Other anemias | 2527 | 1688 | 0.778 | 0.032 | 4.29E-129 |
| 250.2 | Type 2 diabetes | 459.9 | Circulatory disease NEC | 3491 | 1925 | 0.624 | 0.026 | 1.42E-124 |
| 250.2 | Type 2 diabetes | 585.1 | Acute renal failure | 3340 | 2354 | 0.864 | 0.029 | 8.46E-189 |
| 250.2 | Type 2 diabetes | Death | Death | 3739 | 3615 | 0.361 | 0.025 | 6.31E-49 |
| 272.11 | Hypercholesterolemia | 285 | Other anemias | 3879 | 2494 | 0.739 | 0.026 | 4.52E-179 |
| 272.11 | Hypercholesterolemia | 411.8 | Other chronic ischemic heart disease, unspecified | 6500 | 3509 | 1.279 | 0.022 | 0 |
| 272.11 | Hypercholesterolemia | 428.2 | Heart failure NOS | 3135 | 2017 | 0.807 | 0.030 | 1.07E-158 |
| 272.11 | Hypercholesterolemia | 459.9 | Circulatory disease NEC | 6454 | 3740 | 0.883 | 0.021 | 0 |
| 272.11 | Hypercholesterolemia | 480 | Pneumonia | 2496 | 1766 | 0.486 | 0.031 | 2.89E-54 |
| 272.11 | Hypercholesterolemia | 585.1 | Acute renal failure | 4567 | 3206 | 0.672 | 0.024 | 1.12E-169 |
| 272.11 | Hypercholesterolemia | 585.3 | Chronic renal failure [CKD] | 2525 | 1726 | 0.839 | 0.034 | 2.08E-135 |
| 272.11 | Hypercholesterolemia | Death | Death | 5098 | 5060 | 0.111 | 0.020 | 3.91E-08 |
| 278.1 | Obesity | 285 | Other anemias | 2501 | 1308 | 0.738 | 0.034 | 8.41E-107 |
| 278.1 | Obesity | 585.1 | Acute renal failure | 3263 | 1968 | 0.791 | 0.031 | 2.16E-139 |
| 278.1 | Obesity | Death | Death | 2630 | 2581 | -0.137 | 0.028 | 1.30E-06 |
| 285 | Other anemias | Death | Death | 4612 | 4589 | 1.370 | 0.026 | 0 |
| 401.1 | Essential hypertension | 411.8 | Other chronic ischemic heart disease, unspecified | 8172 | 4478 | 0.907 | 0.020 | 0 |
| 401.1 | Essential hypertension | 414 | Other forms of chronic heart disease | 2913 | 1873 | 0.790 | 0.033 | 8.12E-128 |
| 401.1 | Essential hypertension | 428.2 | Heart failure NOS | 4288 | 2894 | 0.694 | 0.027 | 9.41E-146 |
| 401.1 | Essential hypertension | 459.9 | Circulatory disease NEC | 9164 | 5750 | 0.719 | 0.018 | 0 |
| 401.1 | Essential hypertension | 480 | Pneumonia | 3656 | 2730 | 0.393 | 0.027 | 4.88E-47 |
| 401.1 | Essential hypertension | 585.1 | Acute renal failure | 6777 | 5102 | 0.671 | 0.021 | 1.06E-218 |
| 401.1 | Essential hypertension | 585.3 | Chronic renal failure [CKD] | 3432 | 2551 | 0.651 | 0.030 | 7.25E-104 |
| 401.1 | Essential hypertension | Death | Death | 8261 | 8033 | 0.125 | 0.017 | 3.03E-13 |
| 411.2 | Myocardial infarction | 411.8 | Other chronic ischemic heart disease, unspecified | 5661 | 2952 | 4.557 | 0.049 | 0 |
| 411.2 | Myocardial infarction | Death | Death | 3374 | 2604 | 0.955 | 0.028 | 6.14E-263 |
| 411.3 | Angina pectoris | 459.9 | Circulatory disease NEC | 2495 | 1352 | 1.067 | 0.032 | 8.63E-249 |
| 411.4 | Coronary atherosclerosis | 411.8 | Other chronic ischemic heart disease, unspecified | 7905 | 4970 | 4.785 | 0.050 | 0 |
| 411.4 | Coronary atherosclerosis | 459.9 | Circulatory disease NEC | 3715 | 1934 | 1.049 | 0.026 | 0 |
| 411.4 | Coronary atherosclerosis | 585.1 | Acute renal failure | 2843 | 1769 | 1.003 | 0.031 | 5.82E-237 |
| 411.4 | Coronary atherosclerosis | Death | Death | 3657 | 2709 | 0.637 | 0.025 | 6.68E-147 |
| 411.8 | Other chronic ischemic heart disease, unspecified | 585.1 | Acute renal failure | 3194 | 1884 | 1.113 | 0.030 | 5.13E-298 |
| 411.8 | Other chronic ischemic heart disease, unspecified | Death | Death | 4549 | 3268 | 0.901 | 0.024 | 0 |
| 428.1 | Congestive heart failure (CHF) NOS | Death | Death | 2668 | 2463 | 1.752 | 0.040 | 0 |
| 428.2 | Heart failure NOS | Death | Death | 3584 | 3055 | 1.305 | 0.030 | 0 |
| 433.2 | Occlusion of cerebral arteries | 459.9 | Circulatory disease NEC | 2702 | 1770 | 2.764 | 0.051 | 0 |
| 459.9 | Circulatory disease NEC | 480 | Pneumonia | 2637 | 1525 | 1.380 | 0.035 | 0 |
| 459.9 | Circulatory disease NEC | 585.1 | Acute renal failure | 4380 | 2345 | 1.548 | 0.028 | 0 |
| 459.9 | Circulatory disease NEC | 585.3 | Chronic renal failure [CKD] | 2380 | 1321 | 1.550 | 0.039 | 0 |
| 459.9 | Circulatory disease NEC | Death | Death | 5281 | 5243 | 0.957 | 0.022 | 0 |
| 480 | Pneumonia | Death | Death | 5174 | 3795 | 2.329 | 0.032 | 0 |
| 585.1 | Acute renal failure | Death | Death | 7233 | 7106 | 1.923 | 0.025 | 0 |
| 585.3 | Chronic renal failure [CKD] | Death | Death | 3064 | 2744 | 1.541 | 0.033 | 0 |

**Supplementary Table 8. Uric acid-lowering drugs and their target genes.**

| **Type of drug** | **Drug** | **Protein** | **Target gene** |
| --- | --- | --- | --- |
| Xanthine oxidase inhibitors | Allopurinol | Xanthine oxidase (XO) | XDH |
|  | Febuxostat |  |  |
| Uricosurics | Probenecid | Urate anion transporter 1 (URAT1) | SLC22A12 |
|  |  | Organic anion transporter 1 (OAT1) | SLC22A6 |
|  |  | Organic anion transporter 3 (OAT3) | SLC22A8 |
|  |  | Organic anion transporter 4 (OAT4) | SLC22A11 |
|  | Sulfinpyrazone | Urate anion transporter 1 (URAT1) | SLC22A12 |
|  |  | Multidrug resistance associate protein 1 (MRP1) | ABCC1 |
|  |  | Multidrug resistance associate protein 2 (MRP2) | ABCC2 |
|  | Benzbromarone | Urate anion transporter 1 (URAT1) | SLC22A12 |
|  |  | Multidrug resistance associate protein 1 (MRP1) | ABCC1 |
| Purine nucleoside phosphorylase inhibitors | Ulodesine | Purine nucleoside phosphorylase (PNP) | PNP |

**Supplementary Table 9. Genetic instruments used to proxy uric acid-lowering drug effects.**

| **Target** | **SNP** | **Chr** | **Position (b37)** | **EA** | **NEA** | **EAF** | **Beta** | **SE** | **P value** | **N** |
| --- | --- | --- | --- | --- | --- | --- | --- | --- | --- | --- |
| *XDH* | rs114811598 | 2 | 31116245 | T | C | 0.02 | 0.032 | 0.015 | 3.78E-02 | 284037 |
| *XDH* | rs17010458 | 2 | 31120614 | T | C | 0.22 | -0.009 | 0.004 | 1.49E-02 | 457115 |
| *XDH* | rs6749465 | 2 | 31154708 | A | G | 0.24 | -0.022 | 0.006 | 8.00E-04 | 332279 |
| *XDH* | rs11894926 | 2 | 31164031 | T | C | 0.64 | -0.007 | 0.003 | 2.60E-02 | 446680 |
| *XDH* | rs113457380 | 2 | 31180650 | A | G | 0.02 | 0.052 | 0.022 | 1.97E-02 | 135719 |
| *XDH* | rs2241428 | 2 | 31213733 | A | G | 0.27 | 0.009 | 0.004 | 1.75E-02 | 447632 |
| *XDH* | rs12999804 | 2 | 31245650 | A | T | 0.59 | -0.007 | 0.003 | 2.30E-02 | 446528 |
| *XDH* | rs10206765 | 2 | 31261859 | A | G | 0.26 | -0.016 | 0.004 | 1.28E-04 | 321906 |
| *XDH* | rs2365200 | 2 | 31316526 | A | G | 0.51 | 0.007 | 0.003 | 2.46E-02 | 446526 |
| *XDH* | rs115072267 | 2 | 31337155 | A | G | 0.05 | -0.024 | 0.008 | 2.93E-03 | 455738 |
| *XDH* | rs17010732 | 2 | 31344119 | T | C | 0.89 | 0.012 | 0.006 | 3.06E-02 | 445575 |
| *XDH* | rs143040205 | 2 | 31372131 | A | T | 0.98 | 0.035 | 0.016 | 3.04E-02 | 274545 |
| *XDH* | rs10188925 | 2 | 31421582 | A | G | 0.33 | 0.010 | 0.004 | 1.06E-02 | 456735 |
| *XDH* | rs137907571 | 2 | 31430098 | A | G | 0.02 | 0.035 | 0.013 | 9.60E-03 | 323133 |
| *XDH* | rs72859375 | 2 | 31439885 | T | C | 0.04 | -0.028 | 0.009 | 2.20E-03 | 456129 |
| *XDH* | rs6711403 | 2 | 31443741 | A | G | 0.81 | 0.012 | 0.005 | 8.50E-03 | 455545 |
| *XDH* | rs56106435 | 2 | 31481340 | T | C | 0.02 | -0.060 | 0.018 | 1.09E-03 | 175694 |
| *XDH* | rs650742 | 2 | 31491468 | T | C | 0.12 | -0.012 | 0.005 | 1.84E-02 | 438610 |
| *XDH* | rs207455 | 2 | 31567929 | A | G | 0.90 | -0.013 | 0.006 | 3.40E-02 | 415160 |
| *XDH* | rs66479838 | 2 | 31576188 | A | G | 0.06 | 0.018 | 0.009 | 4.06E-02 | 423697 |
| *XDH* | rs55676250 | 2 | 31583444 | A | G | 0.10 | -0.015 | 0.007 | 3.38E-02 | 318904 |
| *XDH* | rs2070293 | 2 | 31592895 | T | C | 0.45 | -0.009 | 0.003 | 5.07E-03 | 457674 |
| *XDH* | rs4952086 | 2 | 31596955 | A | G | 0.43 | 0.007 | 0.003 | 3.17E-02 | 446681 |
| *XDH* | rs206836 | 2 | 31711568 | T | G | 0.20 | -0.012 | 0.004 | 2.04E-03 | 457681 |
| *XDH* | rs75535837 | 2 | 31733118 | A | G | 0.06 | -0.020 | 0.007 | 3.88E-03 | 457319 |
| *XDH* | rs6749019 | 2 | 31797811 | A | C | 0.53 | -0.008 | 0.003 | 1.54E-02 | 447631 |
| *XDH* | rs35741321 | 2 | 31802395 | C | G | 0.97 | -0.066 | 0.021 | 1.69E-03 | 97247 |
| *XDH* | rs114893941 | 2 | 31812827 | T | G | 0.93 | -0.030 | 0.012 | 1.79E-02 | 312569 |
| *XDH* | rs188620338 | 2 | 31816242 | T | C | 0.99 | -0.048 | 0.024 | 4.41E-02 | 270618 |
| *XDH* | rs139993640 | 2 | 31843427 | T | C | 0.97 | 0.053 | 0.012 | 7.90E-06 | 328833 |
| *XDH* | rs806592 | 2 | 31933889 | A | C | 0.17 | -0.012 | 0.004 | 4.91E-03 | 456735 |
| *XDH* | rs6743903 | 2 | 31956992 | T | G | 0.11 | 0.028 | 0.007 | 6.29E-05 | 299217 |
| *XDH* | rs187602166 | 2 | 32078890 | A | T | 0.98 | 0.039 | 0.016 | 1.73E-02 | 309633 |
| *XDH* | rs115767562 | 2 | 32118246 | A | G | 0.01 | -0.055 | 0.021 | 9.89E-03 | 241226 |
| *XDH* | rs78422323 | 2 | 32118993 | A | C | 0.02 | -0.032 | 0.015 | 3.77E-02 | 283117 |
| *SLC22A12* | rs7947315 | 11 | 63859142 | T | C | 0.38 | 0.058 | 0.004 | 3.87E-49 | 446660 |
| *SLC22A12* | rs2701543 | 11 | 63862123 | A | G | 0.73 | -0.028 | 0.004 | 5.50E-14 | 446681 |
| *SLC22A12* | rs618850 | 11 | 63865622 | T | C | 0.93 | -0.025 | 0.007 | 2.80E-04 | 456425 |
| *SLC22A12* | rs17158803 | 11 | 63868359 | A | G | 0.14 | 0.041 | 0.007 | 7.59E-09 | 422969 |
| *SLC22A12* | rs646384 | 11 | 63875456 | A | G | 0.77 | 0.057 | 0.004 | 4.33E-52 | 446528 |
| *SLC22A12* | rs34856996 | 11 | 63884561 | A | G | 0.06 | 0.055 | 0.009 | 8.17E-11 | 302267 |
| *SLC22A12* | rs11231706 | 11 | 63911297 | T | C | 0.23 | -0.033 | 0.011 | 2.21E-03 | 231414 |
| *SLC22A12* | rs4980508 | 11 | 63941734 | A | C | 0.53 | -0.013 | 0.003 | 5.51E-05 | 389456 |
| *SLC22A12* | rs193100342 | 11 | 63967282 | A | G | 0.03 | 0.032 | 0.014 | 2.80E-02 | 292810 |
| *SLC22A12* | rs618160 | 11 | 64017417 | A | G | 0.68 | 0.023 | 0.004 | 2.03E-10 | 447633 |
| *SLC22A12* | rs589030 | 11 | 64031798 | C | G | 0.42 | 0.007 | 0.003 | 4.16E-02 | 456660 |
| *SLC22A12* | rs117452869 | 11 | 64032041 | A | G | 0.04 | -0.065 | 0.011 | 3.85E-09 | 304889 |
| *SLC22A12* | rs78624926 | 11 | 64044929 | T | C | 0.07 | -0.042 | 0.009 | 5.26E-06 | 297443 |
| *SLC22A12* | rs144121146 | 11 | 64054602 | T | C | 0.99 | -0.118 | 0.027 | 1.76E-05 | 274947 |
| *SLC22A12* | rs142996052 | 11 | 64077519 | T | C | 0.02 | 0.071 | 0.016 | 8.06E-06 | 326248 |
| *SLC22A12* | rs183744018 | 11 | 64093723 | T | C | 0.40 | -0.041 | 0.006 | 5.12E-13 | 402677 |
| *SLC22A12* | rs61886881 | 11 | 64098664 | T | C | 0.05 | -0.045 | 0.011 | 4.70E-05 | 286147 |
| *SLC22A12* | rs11231761 | 11 | 64114062 | A | G | 0.23 | -0.017 | 0.004 | 1.55E-05 | 447633 |
| *SLC22A12* | rs117514762 | 11 | 64135907 | T | C | 0.03 | 0.044 | 0.015 | 3.53E-03 | 288818 |
| *SLC22A12* | rs78167615 | 11 | 64151297 | T | C | 0.98 | -0.112 | 0.015 | 2.10E-14 | 310002 |
| *SLC22A12* | rs79403239 | 11 | 64155348 | A | G | 0.93 | 0.021 | 0.007 | 5.16E-03 | 321910 |
| *SLC22A12* | rs148173600 | 11 | 64156453 | A | G | 0.03 | -0.044 | 0.013 | 5.65E-04 | 289684 |
| *SLC22A12* | rs563536 | 11 | 64196434 | T | C | 0.71 | 0.031 | 0.004 | 5.11E-18 | 456736 |
| *SLC22A12* | rs656785 | 11 | 64213634 | A | G | 0.11 | 0.036 | 0.006 | 1.10E-09 | 439113 |
| *SLC22A12* | rs7119252 | 11 | 64213897 | T | C | 0.66 | 0.018 | 0.004 | 1.25E-06 | 447631 |
| *SLC22A12* | rs2083709 | 11 | 64219679 | A | G | 0.70 | -0.015 | 0.003 | 1.44E-05 | 456739 |
| *SLC22A12* | rs7934271 | 11 | 64221957 | T | C | 0.42 | -0.022 | 0.004 | 2.56E-10 | 447631 |
| *SLC22A12* | rs113296233 | 11 | 64225362 | T | C | 0.04 | -0.066 | 0.012 | 1.97E-08 | 291432 |
| *SLC22A12* | rs61885673 | 11 | 64229977 | T | C | 0.01 | 0.079 | 0.020 | 8.54E-05 | 259790 |
| *SLC22A12* | rs140548213 | 11 | 64232290 | C | G | 0.98 | 0.066 | 0.023 | 3.81E-03 | 134238 |
| *SLC22A12* | rs2845636 | 11 | 64253610 | C | G | 0.87 | -0.038 | 0.005 | 1.10E-13 | 433261 |
| *SLC22A12* | rs2076853 | 11 | 64278637 | A | G | 0.57 | -0.048 | 0.003 | 1.79E-52 | 456734 |
| *SLC22A12* | rs36083007 | 11 | 64289328 | A | G | 0.97 | -0.054 | 0.014 | 1.75E-04 | 290413 |
| *SLC22A12* | rs149003598 | 11 | 64297177 | C | G | 0.02 | 0.068 | 0.017 | 7.93E-05 | 269157 |
| *SLC22A12* | rs75152214 | 11 | 64311113 | A | G | 0.05 | -0.055 | 0.011 | 2.66E-07 | 284646 |
| *SLC22A12* | rs71456318 | 11 | 64332862 | A | C | 0.51 | 0.083 | 0.004 | 2.81E-110 | 457656 |
| *SLC22A12* | rs12801784 | 11 | 64338236 | A | G | 0.07 | -0.061 | 0.009 | 1.95E-11 | 266583 |
| *SLC22A12* | rs531763 | 11 | 64352063 | A | G | 0.56 | -0.116 | 0.004 | 1.58E-246 | 457675 |
| *SLC22A12* | rs67048806 | 11 | 64358100 | A | G | 0.05 | -0.038 | 0.011 | 7.24E-04 | 259567 |
| *SLC22A12* | rs56265379 | 11 | 64364395 | T | C | 0.74 | -0.108 | 0.005 | 1.11E-107 | 446541 |
| *SLC22A12* | rs76945158 | 11 | 64364959 | T | C | 0.05 | 0.079 | 0.012 | 2.27E-11 | 375549 |
| *SLC22A12* | rs11601236 | 11 | 64366529 | C | G | 0.04 | 0.041 | 0.010 | 3.27E-05 | 320375 |
| *SLC22A12* | rs71579899 | 11 | 64375757 | T | C | 0.03 | 0.053 | 0.011 | 1.31E-06 | 317009 |
| *SLC22A12* | rs68036074 | 11 | 64377381 | T | C | 0.03 | -0.102 | 0.015 | 2.46E-12 | 270677 |
| *SLC22A12* | rs12419264 | 11 | 64423885 | T | C | 0.02 | 0.056 | 0.021 | 8.45E-03 | 249487 |
| *SLC22A12* | rs142945882 | 11 | 64445779 | T | C | 0.98 | -0.076 | 0.016 | 2.43E-06 | 274143 |
| *SLC22A12* | rs75374475 | 11 | 64448194 | T | C | 0.89 | 0.088 | 0.008 | 3.26E-31 | 278470 |
| *SLC22A12* | rs143500870 | 11 | 64463226 | A | G | 0.98 | 0.083 | 0.017 | 7.79E-07 | 275856 |
| *SLC22A12* | rs17189800 | 11 | 64465282 | T | C | 0.06 | -0.056 | 0.009 | 2.10E-09 | 288472 |
| *SLC22A12* | rs681791 | 11 | 64507477 | A | G | 0.05 | 0.059 | 0.014 | 1.85E-05 | 144370 |
| *SLC22A12* | rs28398896 | 11 | 64516308 | A | G | 0.09 | 0.061 | 0.014 | 1.85E-05 | 297309 |
| *SLC22A12* | rs11603748 | 11 | 64539338 | T | C | 0.07 | 0.044 | 0.009 | 1.28E-06 | 293930 |
| *SLC22A12* | rs533447 | 11 | 64561908 | T | C | 0.37 | 0.071 | 0.004 | 3.11E-57 | 431458 |
| *SLC22A12* | rs654440 | 11 | 64572403 | C | G | 0.54 | 0.064 | 0.004 | 4.98E-48 | 278626 |
| *SLC22A12* | rs11231879 | 11 | 64581645 | A | G | 0.72 | -0.087 | 0.007 | 2.04E-41 | 284926 |
| *SLC22A12* | rs73494258 | 11 | 64583079 | A | T | 0.12 | 0.019 | 0.008 | 1.09E-02 | 263885 |
| *SLC22A12* | rs2957154 | 11 | 64584987 | T | C | 0.68 | 0.025 | 0.004 | 3.18E-10 | 333048 |
| *SLC22A12* | rs113614262 | 11 | 64586099 | T | C | 0.08 | -0.033 | 0.012 | 4.75E-03 | 131804 |
| *SLC22A12* | rs647145 | 11 | 64594393 | T | G | 0.75 | -0.057 | 0.004 | 1.27E-42 | 431398 |
| *SLC22A12* | rs71539700 | 11 | 64614053 | T | G | 0.94 | 0.033 | 0.010 | 5.72E-04 | 289708 |
| *SLC22A12* | rs67580880 | 11 | 64618711 | T | C | 0.35 | -0.049 | 0.005 | 2.73E-25 | 439237 |
| *SLC22A12* | rs77241001 | 11 | 64619680 | T | C | 0.05 | -0.024 | 0.010 | 2.34E-02 | 295329 |
| *SLC22A12* | rs8855 | 11 | 64621573 | A | G | 0.52 | -0.018 | 0.004 | 9.04E-06 | 423790 |
| *SLC22A12* | rs139949915 | 11 | 64626236 | T | C | 0.02 | 0.045 | 0.018 | 1.43E-02 | 246854 |
| *SLC22A12* | rs11606204 | 11 | 64656069 | A | G | 0.05 | 0.033 | 0.011 | 3.06E-03 | 264916 |
| *SLC22A12* | rs7949144 | 11 | 64658399 | C | G | 0.05 | 0.026 | 0.010 | 8.66E-03 | 286710 |
| *SLC22A12* | rs11604872 | 11 | 64659376 | A | G | 0.04 | -0.067 | 0.014 | 6.41E-07 | 252001 |
| *SLC22A12* | rs149219294 | 11 | 64696996 | A | C | 0.95 | -0.048 | 0.011 | 1.58E-05 | 263364 |
| *SLC22A12* | rs11231903 | 11 | 64697507 | T | C | 0.32 | -0.011 | 0.004 | 1.45E-02 | 304667 |
| *SLC22A12* | rs117874559 | 11 | 64699889 | A | C | 0.02 | 0.037 | 0.017 | 2.76E-02 | 285007 |
| *SLC22A12* | rs117176516 | 11 | 64731234 | T | C | 0.02 | -0.051 | 0.018 | 4.78E-03 | 290167 |
| *SLC22A12* | rs74365834 | 11 | 64736378 | T | C | 0.94 | -0.050 | 0.009 | 1.38E-08 | 428993 |
| *SLC22A12* | rs74509728 | 11 | 64740099 | A | G | 0.27 | 0.045 | 0.004 | 4.41E-27 | 450525 |
| *SLC22A12* | rs192905055 | 11 | 64747521 | A | G | 0.02 | -0.050 | 0.016 | 2.17E-03 | 286971 |
| *SLC22A12* | rs7115455 | 11 | 64764094 | A | T | 0.79 | -0.026 | 0.005 | 2.17E-08 | 422562 |
| *SLC22A12* | rs599670 | 11 | 64771179 | T | G | 0.15 | -0.063 | 0.005 | 2.46E-34 | 425835 |
| *SLC22A12* | rs111546958 | 11 | 64772887 | T | C | 0.05 | -0.045 | 0.010 | 1.86E-06 | 315100 |
| *SLC22A12* | rs78287937 | 11 | 64773101 | T | G | 0.92 | 0.023 | 0.008 | 4.75E-03 | 287271 |
| *SLC22A12* | rs61736632 | 11 | 64789222 | T | C | 0.02 | 0.080 | 0.017 | 3.50E-06 | 319979 |
| *SLC22A12* | rs471772 | 11 | 64790139 | A | G | 0.47 | 0.009 | 0.004 | 1.94E-02 | 428836 |
| *SLC22A12* | rs145644706 | 11 | 64805168 | T | C | 0.02 | -0.052 | 0.020 | 8.66E-03 | 253539 |
| *SLC22A12* | rs10160811 | 11 | 64808647 | A | C | 0.09 | 0.027 | 0.007 | 1.01E-04 | 359154 |
| *SLC22A12* | rs492799 | 11 | 64835306 | A | C | 0.13 | 0.012 | 0.006 | 4.80E-02 | 297705 |
| *SLC22A12* | rs4930696 | 11 | 64839414 | T | C | 0.02 | 0.085 | 0.018 | 9.80E-07 | 286202 |
| *SLC22A12* | rs3850947 | 11 | 64840865 | T | C | 0.59 | 0.012 | 0.004 | 7.59E-04 | 453159 |
| *SLC22A12* | rs3185853 | 11 | 64845636 | T | C | 0.03 | -0.041 | 0.012 | 4.30E-04 | 313773 |
| *SLC22A12* | rs74483671 | 11 | 64848092 | A | T | 0.10 | 0.042 | 0.006 | 2.06E-12 | 457189 |
| *PNP* | rs7154187 | 14 | 20484229 | C | G | 0.79 | -0.017 | 0.006 | 3.08E-03 | 376107 |
| *PNP* | rs34841696 | 14 | 20499084 | A | G | 0.64 | 0.010 | 0.004 | 1.28E-02 | 366061 |
| *PNP* | rs72669071 | 14 | 20568482 | T | C | 0.92 | -0.014 | 0.007 | 4.95E-02 | 294498 |
| *PNP* | rs73575402 | 14 | 20569182 | T | G | 0.10 | -0.026 | 0.011 | 1.60E-02 | 265754 |
| *PNP* | rs113326123 | 14 | 20603315 | A | T | 0.02 | 0.039 | 0.019 | 3.75E-02 | 181464 |
| *PNP* | rs35675334 | 14 | 20614639 | T | C | 0.10 | -0.014 | 0.007 | 3.84E-02 | 321908 |
| *PNP* | rs1953557 | 14 | 20695878 | A | T | 0.41 | -0.012 | 0.006 | 3.93E-02 | 312791 |
| *PNP* | rs145656052 | 14 | 20702798 | A | G | 0.20 | 0.011 | 0.005 | 2.68E-02 | 363056 |
| *PNP* | rs2318892 | 14 | 20719900 | A | T | 0.32 | -0.009 | 0.004 | 3.64E-02 | 457672 |
| *PNP* | rs11624214 | 14 | 20726275 | T | C | 0.52 | -0.008 | 0.004 | 2.41E-02 | 447631 |
| *PNP* | rs72671226 | 14 | 20741403 | A | G | 0.29 | -0.012 | 0.005 | 2.39E-02 | 430533 |
| *PNP* | rs11628038 | 14 | 20757728 | A | G | 0.04 | 0.019 | 0.009 | 3.96E-02 | 338145 |
| *PNP* | rs140536937 | 14 | 20780249 | A | G | 0.01 | 0.070 | 0.031 | 2.56E-02 | 164864 |
| *PNP* | rs61995511 | 14 | 20784916 | A | G | 0.03 | 0.029 | 0.011 | 7.90E-03 | 317665 |
| *PNP* | rs117782839 | 14 | 20803790 | A | G | 0.04 | -0.022 | 0.010 | 3.04E-02 | 319875 |
| *PNP* | rs139592248 | 14 | 20815254 | T | C | 0.03 | -0.027 | 0.013 | 3.15E-02 | 314859 |
| *PNP* | rs1108613 | 14 | 20848963 | T | C | 0.21 | -0.012 | 0.004 | 3.91E-03 | 456737 |
| *PNP* | rs72671282 | 14 | 20851813 | C | G | 0.02 | 0.046 | 0.018 | 9.90E-03 | 271305 |
| *PNP* | rs7150966 | 14 | 20865364 | A | G | 0.91 | -0.013 | 0.006 | 3.51E-02 | 446182 |
| *PNP* | rs55790286 | 14 | 20871846 | A | G | 0.86 | 0.013 | 0.006 | 2.42E-02 | 424988 |
| *PNP* | rs1760898 | 14 | 20872881 | T | G | 0.29 | 0.009 | 0.004 | 1.66E-02 | 429221 |
| *PNP* | rs9888571 | 14 | 20873309 | A | T | 0.13 | -0.030 | 0.012 | 1.71E-02 | 292750 |
| *PNP* | rs79372750 | 14 | 20877122 | A | C | 0.02 | -0.036 | 0.018 | 4.92E-02 | 266681 |
| *PNP* | rs2678685 | 14 | 20879311 | T | G | 0.45 | -0.013 | 0.003 | 8.63E-05 | 439274 |
| *PNP* | rs1760891 | 14 | 20881424 | T | G | 0.17 | 0.018 | 0.005 | 8.95E-05 | 436731 |
| *PNP* | rs11160578 | 14 | 20886990 | T | G | 0.73 | -0.010 | 0.004 | 1.17E-02 | 447635 |
| *PNP* | rs1713408 | 14 | 20903349 | T | C | 0.03 | 0.030 | 0.015 | 4.06E-02 | 285283 |
| *PNP* | rs55852417 | 14 | 20954727 | T | C | 0.82 | -0.011 | 0.006 | 4.68E-02 | 300647 |
| *PNP* | rs78896152 | 14 | 20972219 | T | C | 0.11 | 0.030 | 0.014 | 2.76E-02 | 181846 |
| *PNP* | rs12372884 | 14 | 20985940 | A | T | 0.39 | 0.009 | 0.004 | 2.50E-02 | 423956 |
| *PNP* | rs72674527 | 14 | 21001020 | A | T | 0.02 | 0.048 | 0.015 | 1.76E-03 | 283769 |
| *PNP* | rs61995572 | 14 | 21007835 | A | C | 0.95 | -0.020 | 0.009 | 2.81E-02 | 319197 |
| *PNP* | rs112011490 | 14 | 21063626 | A | G | 0.05 | 0.020 | 0.010 | 4.56E-02 | 280704 |
| *PNP* | rs12882535 | 14 | 21108297 | T | C | 0.48 | -0.008 | 0.003 | 1.46E-02 | 456196 |
| *PNP* | rs147128413 | 14 | 21114932 | T | C | 0.91 | -0.021 | 0.009 | 2.23E-02 | 183792 |
| *PNP* | rs4981311 | 14 | 21143013 | A | G | 0.15 | 0.010 | 0.005 | 4.90E-02 | 449547 |
| *PNP* | rs17242790 | 14 | 21166227 | A | G | 0.04 | -0.037 | 0.015 | 1.27E-02 | 170847 |
| *PNP* | rs75581316 | 14 | 21205816 | T | C | 0.16 | 0.011 | 0.005 | 2.36E-02 | 440252 |
| *PNP* | rs1888566 | 14 | 21210030 | T | G | 0.50 | -0.008 | 0.003 | 1.28E-02 | 456687 |
| *PNP* | rs147120991 | 14 | 21228536 | A | G | 0.02 | 0.030 | 0.015 | 3.99E-02 | 317576 |
| *PNP* | rs113470494 | 14 | 21246907 | A | C | 0.13 | 0.013 | 0.006 | 2.89E-02 | 433606 |
| *PNP* | rs4982356 | 14 | 21249030 | A | T | 0.51 | -0.007 | 0.003 | 3.93E-02 | 447130 |
| *PNP* | rs34232075 | 14 | 21275759 | C | G | 0.80 | 0.011 | 0.005 | 2.59E-02 | 429811 |
| *PNP* | rs4246988 | 14 | 21308425 | A | G | 0.34 | 0.008 | 0.004 | 4.66E-02 | 368828 |
| *PNP* | rs8011970 | 14 | 21318633 | A | G | 0.18 | -0.013 | 0.006 | 3.05E-02 | 304243 |
| *PNP* | rs11844872 | 14 | 21335191 | A | G | 0.72 | 0.032 | 0.013 | 1.57E-02 | 160039 |
| *PNP* | rs56139183 | 14 | 21340314 | C | G | 0.14 | -0.014 | 0.006 | 2.06E-02 | 290303 |
| *PNP* | rs117271587 | 14 | 21341538 | T | G | 0.02 | 0.037 | 0.018 | 4.05E-02 | 255858 |
| *PNP* | rs6571588 | 14 | 21438849 | A | G | 0.59 | 0.006 | 0.003 | 4.39E-02 | 456735 |
| *PNP* | rs116790800 | 14 | 21439290 | T | C | 0.96 | 0.020 | 0.010 | 4.19E-02 | 431520 |

**Supplementary Table 10. Effect estimates of genetically proxied uric acid-lowering drugs on the risk of associated outcomes identified by PheWAS.**

| **Phenotype** | **Description** | **XOI** | | **SLC22A12 inhibitor** | | **PNP inhibitor** | |
| --- | --- | --- | --- | --- | --- | --- | --- |
|  |  | **OR (95% CI)** | **P value** | **OR (95% CI)** | **P value** | **OR (95% CI)** | **P value** |
| 10 | Tuberculosis | 0.84 (0.69, 1.02) | 0.079 | 0.96 (0.93, 1.00) | 0.032 | 1.00 (0.99, 1.00) | 0.340 |
| 41.1 | Staphylococcus infections | 0.92 (0.58, 1.44) | 0.711 | 1.04 (0.96, 1.12) | 0.317 | 0.99 (0.99, 1.00) | 0.203 |
| 244.4 | Hypothyroidism NOS | 0.82 (0.66, 1.02) | 0.074 | 0.96 (0.93, 1.00) | 0.029 | 1.00 (0.99, 1.00) | 0.564 |
| 250.2 | Type 2 diabetes | 0.80 (0.66, 0.98) | 0.030 | 1.00 (0.97, 1.03) | 0.999 | 1.00 (1.00, 1.01) | 0.378 |
| 272.11 | Hypercholesterolemia | 0.87 (0.75, 1.02) | 0.082 | 0.96 (0.94, 0.99) | 0.004 | 1.00 (1.00, 1.00) | 0.304 |
| 274.1 | Gout | 0.70 (0.52, 0.95) | 0.020 | 0.73 (0.70, 0.77) | 8.31E-34 | 1.00 (1.00, 1.01) | 0.826 |
| 274.11 | Gouty arthropathy | 0.77 (0.28, 2.15) | 0.619 | 0.78 (0.66, 0.93) | 0.006 | 0.98 (0.96, 1.00) | 0.023 |
| 276.13 | Hyperpotassemia | 0.66 (0.37, 1.18) | 0.159 | 0.96 (0.87, 1.05) | 0.369 | 1.00 (0.99, 1.01) | 0.875 |
| 278.1 | Obesity | 0.74 (0.61, 0.90) | 0.003 | 1.03 (1.00, 1.07) | 0.073 | 1.00 (1.00, 1.00) | 0.753 |
| 285 | Other anemias | 0.87 (0.70, 1.09) | 0.227 | 1.02 (0.98, 1.05) | 0.432 | 1.00 (1.00, 1.01) | 0.105 |
| 401.1 | Essential hypertension | 0.89 (0.78, 1.01) | 0.069 | 0.99 (0.97, 1.01) | 0.475 | 1.00 (1.00, 1.00) | 0.578 |
| 411.1 | Unstable angina (intermediate coronary syndrome) | 0.83 (0.54, 1.27) | 0.388 | 1.00 (0.93, 1.08) | 0.988 | 0.99 (0.99, 1.00) | 0.114 |
| 411.2 | Myocardial infarction | 0.82 (0.64, 1.05) | 0.108 | 0.99 (0.95, 1.03) | 0.579 | 1.00 (1.00, 1.01) | 0.639 |
| 411.3 | Angina pectoris | 0.79 (0.63, 1.00) | 0.055 | 0.98 (0.94, 1.02) | 0.330 | 1.00 (0.99, 1.00) | 0.340 |
| 411.4 | Coronary atherosclerosis | 0.82 (0.67, 1.02) | 0.070 | 0.96 (0.93, 1.00) | 0.049 | 1.00 (0.99, 1.00) | 0.243 |
| 411.8 | Other chronic ischemic heart disease, unspecified | 0.91 (0.76, 1.08) | 0.271 | 1.01 (0.98, 1.04) | 0.591 | 1.00 (0.99, 1.00) | 0.109 |
| 414 | Other forms of chronic heart disease | 1.14 (0.78, 1.66) | 0.498 | 1.00 (0.94, 1.07) | 0.885 | 1.00 (0.99, 1.01) | 0.919 |
| 428.1 | Congestive heart failure (CHF) NOS | 0.64 (0.42, 0.99) | 0.043 | 0.98 (0.91, 1.06) | 0.641 | 1.00 (0.99, 1.01) | 0.802 |
| 428.2 | Heart failure NOS | 0.85 (0.63, 1.16) | 0.310 | 0.97 (0.92, 1.02) | 0.236 | 1.00 (0.99, 1.00) | 0.713 |
| 433.2 | Occlusion of cerebral arteries | 0.80 (0.53, 1.20) | 0.276 | 0.93 (0.87, 1.00) | 0.044 | 1.00 (0.99, 1.01) | 0.931 |
| 433.21 | Cerebral artery occlusion, with cerebral infarction | 0.79 (0.51, 1.22) | 0.282 | 0.99 (0.92, 1.07) | 0.839 | 1.00 (0.99, 1.00) | 0.372 |
| 433.31 | Transient cerebral ischemia | 0.80 (0.49, 1.29) | 0.359 | 0.93 (0.85, 1.01) | 0.073 | 1.00 (0.99, 1.01) | 0.521 |
| 440.2 | Atherosclerosis of the extremities | 0.72 (0.31, 1.68) | 0.444 | 1.00 (0.87, 1.16) | 0.956 | 1.02 (1.00, 1.03) | 0.042 |
| 441.1 | Acute vascular insufficiency of intestine | 1.13 (0.32, 3.98) | 0.849 | 0.86 (0.69, 1.06) | 0.155 | 0.99 (0.97, 1.02) | 0.524 |
| 443.9 | Peripheral vascular disease, unspecified | 0.60 (0.38, 0.94) | 0.025 | 1.00 (0.93, 1.08) | 0.901 | 1.00 (1.00, 1.01) | 0.281 |
| 459.9 | Circulatory disease NEC | 0.91 (0.75, 1.11) | 0.348 | 0.99 (0.96, 1.03) | 0.739 | 1.00 (1.00, 1.00) | 0.735 |
| 480 | Pneumonia | 0.89 (0.69, 1.15) | 0.374 | 0.99 (0.94, 1.03) | 0.555 | 1.00 (1.00, 1.01) | 0.457 |
| 557.1 | Celiac disease | 0.76 (0.41, 1.41) | 0.383 | 0.97 (0.87, 1.08) | 0.569 | 1.01 (1.00, 1.02) | 0.233 |
| 580.2 | Nephrotic syndrome without mention of glomerulonephritis | 0.79 (0.30, 2.05) | 0.620 | 1.02 (0.87, 1.21) | 0.774 | 1.00 (0.98, 1.02) | 0.798 |
| 585.1 | Acute renal failure | 0.75 (0.58, 0.97) | 0.027 | 0.99 (0.95, 1.03) | 0.671 | 1.00 (0.99, 1.00) | 0.195 |
| 585.3 | Chronic renal failure [CKD] | 0.80 (0.56, 1.15) | 0.233 | 0.97 (0.91, 1.03) | 0.341 | 1.00 (1.00, 1.01) | 0.425 |
| 585.31 | Renal dialysis | 1.02 (0.33, 3.19) | 0.972 | 1.07 (0.88, 1.30) | 0.489 | 0.99 (0.97, 1.02) | 0.596 |
| 711.1 | Pyogenic arthritis | 0.31 (0.08, 1.13) | 0.076 | 1.18 (0.94, 1.47) | 0.149 | 1.02 (0.99, 1.04) | 0.179 |
| 717 | Polymyalgia Rheumatica | 0.71 (0.42, 1.20) | 0.203 | 0.97 (0.89, 1.06) | 0.551 | 1.01 (1.00, 1.02) | 0.236 |
| - | Circulatory mortality | 0.92 (0.67, 1.26) | 0.598 | 0.97 (0.92, 1.02) | 0.213 | 1.00 (1.00, 1.01) | 0.559 |

**Supplementary Table 11. Genetic instruments used to proxy antihypertensive drug effects.**

| **Type** | **SNP** | **Chr** | **Position (b37)** | **EA** | **NEA** | **EAF** | **Beta** | **SE** | **P value** | **N** | **Gene** |
| --- | --- | --- | --- | --- | --- | --- | --- | --- | --- | --- | --- |
| ACEI | rs4291 | 17 | 61554194 | A | T | 0.62 | -0.284 | 0.031 | 8.65E-20 | 745820 | *ACE* |
| Beta-blockers (BBs) | rs11196549 | 10 | 115707298 | A | G | 0.04 | 0.688 | 0.078 | 1.58E-18 | 738169 | *NHLRC2* |
| Beta-blockers (BBs) | rs460718 | 10 | 115721364 | A | G | 0.33 | -0.276 | 0.032 | 1.36E-17 | 738169 | *NHLRC2* |
| Beta-blockers (BBs) | rs11196597 | 10 | 115788094 | A | G | 0.13 | 0.286 | 0.046 | 4.23E-10 | 737164 | *ADRB1* |
| Beta-blockers (BBs) | rs17875473 | 10 | 115800294 | T | C | 0.09 | 0.328 | 0.055 | 2.66E-09 | 738170 | *ADRB1* |
| Beta-blockers (BBs) | rs1801253 | 10 | 115805056 | C | G | 0.73 | 0.463 | 0.034 | 2.84E-41 | 738169 | *ADRB1* |
| Beta-blockers (BBs) | rs4359161 | 10 | 115826508 | A | G | 0.18 | -0.266 | 0.039 | 9.46E-12 | 738168 | *ADRB1* |
| Calcium channel blockers (CCBs) | rs3821843 | 3 | 53558012 | A | G | 0.68 | 0.337 | 0.034 | 6.56E-24 | 736049 | *CACNA1D* |
| Calcium channel blockers (CCBs) | rs114987861 | 3 | 53605712 | A | G | 0.03 | 0.529 | 0.096 | 3.36E-08 | 737054 | *CACNA1D* |
| Calcium channel blockers (CCBs) | rs113210396 | 3 | 53612327 | T | G | 0.05 | -0.434 | 0.077 | 1.76E-08 | 737164 | *CACNA1D* |
| Calcium channel blockers (CCBs) | rs7340705 | 3 | 53734443 | T | C | 0.67 | -0.243 | 0.032 | 4.87E-14 | 738169 | *CACNA1D* |
| Calcium channel blockers (CCBs) | rs2488136 | 10 | 18334521 | A | G | 0.29 | 0.226 | 0.033 | 1.22E-11 | 738169 | *SLC39A12* |
| Calcium channel blockers (CCBs) | rs1888693 | 10 | 18440444 | A | G | 0.34 | 0.386 | 0.032 | 4.69E-34 | 736050 | *CACNB2* |
| Calcium channel blockers (CCBs) | rs16916914 | 10 | 18457722 | T | C | 0.96 | -0.564 | 0.081 | 2.72E-12 | 737424 | *CACNB2* |
| Calcium channel blockers (CCBs) | rs7076319 | 10 | 18459450 | A | G | 0.73 | -0.321 | 0.034 | 5.07E-21 | 737054 | *CACNB2* |
| Calcium channel blockers (CCBs) | rs61278674 | 10 | 18481737 | A | G | 0.91 | -0.330 | 0.054 | 1.03E-09 | 737163 | *CACNB2* |
| Calcium channel blockers (CCBs) | rs1779209 | 10 | 18514561 | T | C | 0.29 | 0.274 | 0.034 | 4.23E-16 | 729448 | *CACNB2* |
| Calcium channel blockers (CCBs) | rs10828399 | 10 | 18553968 | A | G | 0.52 | -0.195 | 0.030 | 1.10E-10 | 738168 | *CACNB2* |
| Calcium channel blockers (CCBs) | rs10828452 | 10 | 18592450 | A | T | 0.79 | 0.305 | 0.039 | 4.20E-15 | 737164 | *CACNB2* |
| Calcium channel blockers (CCBs) | rs10828542 | 10 | 18627285 | A | G | 0.61 | 0.182 | 0.031 | 5.18E-09 | 738170 | *CACNB2* |
| Calcium channel blockers (CCBs) | rs12780039 | 10 | 18678987 | C | G | 0.12 | 0.285 | 0.047 | 1.26E-09 | 738167 | *CACNB2* |
| Calcium channel blockers (CCBs) | rs112133583 | 10 | 18695681 | T | C | 0.03 | -0.555 | 0.097 | 1.18E-08 | 737169 | *CACNB2* |
| Calcium channel blockers (CCBs) | rs11014170 | 10 | 18710991 | A | G | 0.02 | -0.670 | 0.115 | 5.61E-09 | 732148 | *CACNB2* |
| Calcium channel blockers (CCBs) | rs7923191 | 10 | 18727901 | A | G | 0.79 | -0.369 | 0.038 | 1.10E-22 | 737054 | *CACNB2* |
| Calcium channel blockers (CCBs) | rs12258967 | 10 | 18727959 | C | G | 0.70 | 0.633 | 0.034 | 1.08E-78 | 737165 | *CACNB2* |
| Calcium channel blockers (CCBs) | rs72786098 | 10 | 18729855 | A | G | 0.03 | -0.503 | 0.088 | 1.18E-08 | 737055 | *CACNB2* |
| Calcium channel blockers (CCBs) | rs1998822 | 10 | 18755664 | A | G | 0.72 | -0.196 | 0.034 | 1.15E-08 | 727331 | *CACNB2* |
| Calcium channel blockers (CCBs) | rs4748474 | 10 | 18790727 | A | G | 0.52 | 0.195 | 0.030 | 1.61E-10 | 729908 | *CACNB2* |
| Calcium channel blockers (CCBs) | rs150857355 | 12 | 49209340 | C | G | 0.02 | 0.941 | 0.112 | 5.20E-17 | 731300 | *CACNB3* |
| Calcium channel blockers (CCBs) | rs2239046 | 12 | 2434419 | A | G | 0.68 | 0.208 | 0.032 | 9.58E-11 | 745818 | *CACNA1C* |
| Calcium channel blockers (CCBs) | rs714277 | 12 | 2514270 | T | C | 0.28 | 0.199 | 0.033 | 2.38E-09 | 745820 | *CACNA1C* |

**Supplementary Table 12. Factorial MR estimates for the join effect of combination therapy integrating uric acid-lowering and antihypertensive drugs on the risk of cardiovascular outcomes.**

| **Combination therapy** | **XOI** | | **SLC22A12 inhibitor** | | **PNP inhibitor** | |
| --- | --- | --- | --- | --- | --- | --- |
|  | **OR (95% CI)** | **P value** | **OR (95% CI)** | **P value** | **OR (95% CI)** | **P value** |
| **Coronary atherosclerosis** | | | | | | |
| ACEI | | | | | | |
| Higher UA and higher SBP | 1 [Reference] |  | 1 [Reference] |  | 1 [Reference] |  |
| Lower UA and higher SBP | 0.99 (0.96, 1.02) | 0.426 | 0.96 (0.93, 0.99) | 0.005 | 0.99 (0.95, 1.02) | 0.343 |
| Higher UA and lower SBP | 0.97 (0.94, 1.01) | 0.117 | 0.95 (0.92, 0.98) | 0.003 | 0.97 (0.93, 1.00) | 0.056 |
| Lower UA and lower SBP | 0.96 (0.92, 0.99) | 0.012 | 0.95 (0.92, 0.98) | 0.003 | 0.96 (0.93, 0.99) | 0.017 |
| BB | | | | | | |
| Higher UA and higher SBP | 1 [Reference] |  | 1 [Reference] |  | 1 [Reference] |  |
| Lower UA and higher SBP | 1.00 (0.97, 1.03) | 0.964 | 1.00 (0.96, 1.03) | 0.780 | 1.00 (0.96, 1.03) | 0.838 |
| Higher UA and lower SBP | 0.98 (0.94, 1.01) | 0.144 | 0.98 (0.95, 1.01) | 0.225 | 0.97 (0.94, 1.00) | 0.058 |
| Lower UA and lower SBP | 0.95 (0.92, 0.98) | 0.002 | 0.94 (0.90, 0.97) | 0 | 0.95 (0.92, 0.98) | 0.002 |
| CCB | | | | | | |
| Higher UA and higher SBP | 1 [Reference] |  | 1 [Reference] |  | 1 [Reference] |  |
| Lower UA and higher SBP | 0.98 (0.95, 1.02) | 0.280 | 0.97 (0.93, 1.00) | 0.039 | 0.97 (0.94, 1.01) | 0.119 |
| Higher UA and lower SBP | 0.95 (0.92, 0.98) | 0.002 | 0.94 (0.91, 0.97) | 0 | 0.94 (0.91, 0.97) | 0 |
| Lower UA and lower SBP | 0.94 (0.91, 0.97) | 0 | 0.93 (0.90, 0.96) | 0 | 0.94 (0.91, 0.97) | 0 |
| **Congestive heart failure** | | | | | | |
| ACEI | | | | | | |
| Higher UA and higher SBP | 1 [Reference] |  | 1 [Reference] |  | 1 [Reference] |  |
| Lower UA and higher SBP | 0.97 (0.92, 1.04) | 0.428 | 0.99 (0.93, 1.05) | 0.768 | 1.00 (0.94, 1.06) | 0.966 |
| Higher UA and lower SBP | 0.95 (0.88, 1.01) | 0.116 | 0.93 (0.87, 1.00) | 0.058 | 0.93 (0.86, 0.99) | 0.030 |
| Lower UA and lower SBP | 0.90 (0.84, 0.97) | 0.004 | 0.93 (0.87, 1.00) | 0.050 | 0.94 (0.88, 1.01) | 0.102 |
| BB | | | | | | |
| Higher UA and higher SBP | 1 [Reference] |  | 1 [Reference] |  | 1 [Reference] |  |
| Lower UA and higher SBP | 0.96 (0.89, 1.03) | 0.211 | 1.02 (0.95, 1.09) | 0.596 | 1.01 (0.94, 1.08) | 0.754 |
| Higher UA and lower SBP | 0.97 (0.90, 1.03) | 0.311 | 1.00 (0.93, 1.07) | 0.906 | 0.98 (0.91, 1.04) | 0.473 |
| Lower UA and lower SBP | 0.94 (0.88, 1.01) | 0.072 | 0.96 (0.90, 1.03) | 0.289 | 0.98 (0.91, 1.05) | 0.496 |
| CCB | | | | | | |
| Higher UA and higher SBP | 1 [Reference] |  | 1 [Reference] |  | 1 [Reference] |  |
| Lower UA and higher SBP | 0.98 (0.92, 1.05) | 0.596 | 0.96 (0.90, 1.03) | 0.289 | 1.00 (0.94, 1.07) | 0.983 |
| Higher UA and lower SBP | 0.97 (0.90, 1.04) | 0.357 | 0.92 (0.86, 0.99) | 0.019 | 0.95 (0.88, 1.01) | 0.107 |
| Lower UA and lower SBP | 0.92 (0.86, 0.99) | 0.023 | 0.94 (0.88, 1.01) | 0.085 | 0.96 (0.89, 1.03) | 0.214 |
| **Occlusion of cerebral arteries** | | | | | | |
| ACEI | | | | | | |
| Higher UA and higher SBP | 1 [Reference] |  | 1 [Reference] |  | 1 [Reference] |  |
| Lower UA and higher SBP | 0.95 (0.89, 1.01) | 0.079 | 0.97 (0.91, 1.03) | 0.345 | 1.01 (0.95, 1.07) | 0.816 |
| Higher UA and lower SBP | 0.96 (0.89, 1.02) | 0.182 | 0.97 (0.91, 1.04) | 0.452 | 0.97 (0.91, 1.04) | 0.378 |
| Lower UA and lower SBP | 0.95 (0.89, 1.01) | 0.117 | 0.95 (0.89, 1.02) | 0.144 | 0.99 (0.93, 1.06) | 0.804 |
| BB | | | | | | |
| Higher UA and higher SBP | 1 [Reference] |  | 1 [Reference] |  | 1 [Reference] |  |
| Lower UA and higher SBP | 0.94 (0.88, 1.00) | 0.053 | 0.98 (0.92, 1.05) | 0.616 | 1.00 (0.94, 1.07) | 0.972 |
| Higher UA and lower SBP | 0.99 (0.92, 1.05) | 0.699 | 1.03 (0.97, 1.10) | 0.346 | 1.00 (0.94, 1.07) | 0.947 |
| Lower UA and lower SBP | 0.98 (0.92, 1.05) | 0.610 | 0.99 (0.93, 1.06) | 0.815 | 1.03 (0.96, 1.10) | 0.382 |
| CCB | | | | | | |
| Higher UA and higher SBP | 1 [Reference] |  | 1 [Reference] |  | 1 [Reference] |  |
| Lower UA and higher SBP | 1.01 (0.95, 1.08) | 0.705 | 0.98 (0.92, 1.04) | 0.477 | 1.06 (1.00, 1.13) | 0.069 |
| Higher UA and lower SBP | 1.00 (0.93, 1.06) | 0.934 | 0.96 (0.89, 1.02) | 0.171 | 1.00 (0.93, 1.07) | 0.935 |
| Lower UA and lower SBP | 0.92 (0.86, 0.98) | 0.011 | 0.92 (0.86, 0.99) | 0.018 | 0.96 (0.90, 1.03) | 0.228 |
| **Peripheral vascular disease** | | | | | | |
| ACEI | | | | | | |
| Higher UA and higher SBP | 1 [Reference] |  | 1 [Reference] |  | 1 [Reference] |  |
| Lower UA and higher SBP | 0.96 (0.90, 1.03) | 0.216 | 1.03 (0.96, 1.10) | 0.464 | 1.05 (0.98, 1.12) | 0.168 |
| Higher UA and lower SBP | 1.03 (0.95, 1.10) | 0.489 | 1.04 (0.97, 1.12) | 0.271 | 1.06 (0.99, 1.14) | 0.111 |
| Lower UA and lower SBP | 0.99 (0.92, 1.07) | 0.812 | 1.04 (0.97, 1.12) | 0.240 | 1.05 (0.98, 1.13) | 0.189 |
| BB | | | | | | |
| Higher UA and higher SBP | 1 [Reference] |  | 1 [Reference] |  | 1 [Reference] |  |
| Lower UA and higher SBP | 0.94 (0.87, 1.01) | 0.075 | 1.00 (0.93, 1.07) | 0.962 | 1.03 (0.96, 1.10) | 0.455 |
| Higher UA and lower SBP | 0.95 (0.89, 1.02) | 0.175 | 0.96 (0.89, 1.03) | 0.245 | 0.97 (0.91, 1.05) | 0.491 |
| Lower UA and lower SBP | 0.94 (0.87, 1.01) | 0.075 | 0.99 (0.92, 1.06) | 0.702 | 0.99 (0.92, 1.07) | 0.866 |
| CCB | | | | | | |
| Higher UA and higher SBP | 1 [Reference] |  | 1 [Reference] |  | 1 [Reference] |  |
| Lower UA and higher SBP | 0.96 (0.89, 1.03) | 0.218 | 1.02 (0.95, 1.09) | 0.614 | 1.01 (0.94, 1.08) | 0.890 |
| Higher UA and lower SBP | 0.93 (0.87, 1.00) | 0.051 | 0.94 (0.87, 1.01) | 0.089 | 0.92 (0.86, 0.99) | 0.027 |
| Lower UA and lower SBP | 0.90 (0.84, 0.97) | 0.004 | 0.95 (0.88, 1.02) | 0.159 | 0.96 (0.89, 1.03) | 0.257 |
