# Supplementary material for: A phenome-wide association and factorial Mendelian randomization study on the repurposing of uric acid-lowering drugs for cardiovascular outcomes
Source: Eur J Epidemiol. 2024 Jul 11;39(8):869–80. doi: 10.1007/s10654-024-01138-0 (PMC11410910; doi:10.1007/s10654-024-01138-0)
Supplement: Supplementary file 2 — Supplementary Material 2 [file 10654_2024_1138_MOESM2_ESM.docx]

**Supplementary Methods**

1. **Mapping national medical registry records to phecodes**

There are several steps we applied for mapping national medical registry records to phecodes:

1. combine records (i.e., ICD codes) from three types of national medical registries (i.e., in-patient hospital episode, cancer registry, and death registry records) and exclude duplicates (if an individual has multiple records of the same disease, only the initial diagnosis record is retained);
2. map ICD codes to phecodes based on the mapping files downloaded from a publicly available website: PheWAS catalog (https://phewascatalog.org/); as the UK Biobank contains both ICD9 and ICD10 codes, both mapping files (phecode_icd9_map_unrolled.csv and Phecode_map_v1_2_icd10_beta.csv) were used.
3. **Mapping primary care records to phecodes**

**Primary care data**

Within the UK healthcare setting, individuals seeking advice or treatment for a health concern normally first meet with a family physician (known as a General Practitioner, or GP) or a nurse (for example, a Nurse Practitioner) at their local general practice. GPs can refer patients who require more specialized treatment (or further tests) to hospital or other community-based services. There is a wealth of information available within primary care records. The UK Biobank has been liaising with various data suppliers and other intermediaries (including the main primary care computer system suppliers in England) to obtain primary care data for UK Biobank participants, all of whom have provided written consent for linkage to their health-related records. To date, coded data have been obtained for approximately 45% of the UK Biobank cohort (~230,000 participants) and are now available as part of this interim release. This dataset contains coded clinical events including consultations, diagnoses, procedures and laboratory tests, prescribed medications including prescription date, drug code and, where available, drug name and quantity, and a range of administrative codes (e.g. referrals to specialist hospital clinics).

There are two versions of read codes used in primary care since 1985: version 2 (Read V2) and version 3 (CTV3 or Read V3). In general, Read V2 was introduced in the early 1990s and formed the basis for clinical coding in UK primary care. It consists of alphanumeric codes and covers a wide range of medical terms, diagnoses, symptoms, procedures, medications, and other clinical concepts. CTV3, an extension of Read V2, was developed to enhance and expand the coding capabilities of Read V2. CTV3 includes additional codes to capture new clinical concepts, emerging conditions, and advancements in medical knowledge. It provides a more comprehensive and detailed representation of clinical information. The UK Biobank primary care data incorporates both Read V2 and CTV3 codes to capture a broad range of healthcare information from participants' primary care records.

**Mapping read codes to phecodes**

The primary care data consist of three data tables including gp_registration.txt, gp_scripts.txt, gp_clinical.txt, which can be downloaded from the UK Biobank.^1^ The data table gp_clinical.txt contains clinical event data for 230,106 participants and has eight fields including participant identifier, data provider, data of event, three free text “value” fields, Read V2 and CTV3 codes. Based on the data provided, we applied a systematic data-driven approach to map primary care records to phecodes. Here are the main steps:

1. remove Read V2/CTV3 codes related to drug prescription events, occupations, examinations, symptoms, injuries, diagnostic and laboratory procedures from gp_clinical.txt file;
2. map the remaining Read V2/CTV3 codes to ICD-9 or ICD-10 (recommend) using the UK Biobank cross-mapping tables provided in the Resource 592;
3. for Read V2/CTV3 codes that no mapping was provided by the cross-mapping file, use UK Biobank coding19.txt to manually convert Read descriptions to ICD-9 or ICD-10 (recommend) based on the Fuzzy mapping algorithm.^2, 3^ In this study, a similarity score of 0.8 was used as a cut-off threshold to exclude less relevant records, followed by a manual double-check of the remaining records;
4. map ICD-9 or ICD-10 (recommend) codes to phecodes using the mapping file provided by the PheWAS catalog website (Phecode_map_v1_2_icd10_beta.csv).

**Reference**

1. UK Biobank Primary Care Linked Data. Available: http://biobank.ndph.ox.ac.uk/showcase/showcase/docs/primary_care_data.pdf
2. Rancho Term Mapping Solution (Fuzzy Tool). 2021. Available: <https://ranchobiosciences.com/wpcontent/uploads/2021/11/Rancho-Fuzzy-Tool-for-Term-Mapping.pdf>
3. Fuzzy string matching with trigram and trigraphs. Available: https://www.postgresonline.com/journal/archives/169-Fuzzy-string-matching-with-Trigram-and-Trigraphs.html
